# ^*^OH Adsorption‐Mediated Electrochemical Oxidation of 5‐Hydroxymethylfurfural to Selective 2,5‐Furandicarboxylic Acid at pH 12

**DOI:** 10.1002/advs.202518349

**Published:** 2025-12-12

**Authors:** Eunchong Lee, Jinwoo Hwang, Suhwan Yoo, Juhyung Choi, Hyun Ji An, Sang Heon Han, Taerin Kim, Jeong Woo Han, Yun Jeong Hwang

**Affiliations:** ^1^ Department of Chemistry College of Natural Sciences Seoul National University Seoul 08826 Republic of Korea; ^2^ Department of Materials Science and Engineering Research Institute of Advanced Materials Seoul National University Seoul 08826 Republic of Korea

**Keywords:** 2,5‐furandicarboxylic acid (FDCA), 5‐hydroxymethylfurfural (HMF), interface‐rich Cu‐Ni catalyst, mildly alkaline media, water structure

## Abstract

Strongly alkaline electrolytes are typically used for electrochemical 5‐hydroxymethylfurfural oxidation (HMFOR) to selective 2,5‐furandicarboxylic acid (FDCA) production, despite HMF degradation. Although HMF remains stable in weakly alkaline media, slow kinetics due to limited OH^−^ concentration hinder active FDCA formation. Herein, unlike the CuO or NiOOH catalyst, significantly improved direct aldehyde oxidation is demonstrated via the utilization of adsorbed OH (^*^OH) on the CuO/NiOOH interface surface, enabling complete conversion to FDCA even at mild pH. *Operando* measurements reveal that the CuO/NiOOH interface serves as a synergistic active site: Cu sites enhance ^*^OH adsorption and utilization, while Ni sites promote HMF dehydrogenation through rapid Ni(OH)_2_/NiOOH redox cycling. The synergistic effect promotes highly selective FDCA production while preventing premature desorption of intermediates, which leads to outperforming activity compared with individual CuO and Ni(OH)_2_. The interface‐enriched CuO@NiOOH catalyst delivers >95% FDCA yield and Faradaic efficiency, and maintains long‐term stability for 32.8 h under large‐volume operation, owing to the enhanced HMF stability at pH 12. Water structure investigation further supports that the electrolyte microenvironment significantly affects HMFOR kinetics alongside the intrinsic activity of the interface. These insights provide a strategy for designing catalysts capable of efficient biomass‐to‐FDCA conversion across a broader pH range.

## Graphical abstract

Under the weakly alkaline condition, at pH 12, the ^*^OH adsorption–mediated mechanism of direct aldehyde oxidation is demonstrated to complete HMF oxidation to FDCA, obtaining >95% selectivity and yield. In OH^−^‐deficient media, high FDCA selectivity is achieved on the CuO/NiOOH interface, a synergistic active site, where Cu enhances ^*^OH utilization, and Ni facilitates rapid redox‐driven dehydrogenation.

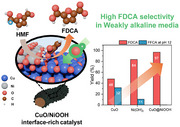

## 1 Introduction

Electrochemical valorization of biomass‐derived carbon sources offers a promising pathway toward sustainable carbon cycles by replacing petroleum‐based carbon sources in the chemical industry.^[^ ^1^, ^2^ ^]^ The electrochemical oxidation of 5‐hydroxymethylfurfural (HMF), a key biomass‐derived platform chemical, enables the production of 2,5‐furandicarboxylic acid (FDCA) by utilizing the non‐noble metals under benign pressure and temperature conditions.^[^ ^3^, ^4^, ^5^, ^6^ ^]^ As an alternative to terephthalic acid, FDCA can be used to synthesize poly(ethylene furoate) (PEF), which exhibits superior mechanical properties comparable to those of poly(ethylene terephthalate) (PET).^[^ ^7^ ^]^ Additionally, the electrochemical HMF oxidation reaction (HMFOR) has a lower overpotential (0.113 V vs NHE) than the oxygen evolution reaction (OER, 1.23 V vs NHE), making FDCA production a more energy‐efficient upcycling strategy when paired with the hydrogen evolution reaction (HER) or CO_2_ reduction reaction.^[^ ^8^, ^9^, ^10^ ^]^

The electrochemical oxidation of alcohol and aldehyde groups has been extensively studied under strongly alkaline conditions (≈pH 14), particularly with Ni‐based catalysts.^[^ ^11^, ^12^, ^13^ ^]^ In these systems, alcohol oxidation is commonly explained by a dehydrogenation mechanism involving hydrogen atom transfer from the α‐carbon of the alcohol to the µ‐O site in active Ni(OH)O domains.^[^ ^14^, ^15^, ^16^ ^]^ Meanwhile, the OH^−^ ions in alkaline conditions act as reactants for HMFOR and facilitate the formation of the active NiOOH phase under anodic potential.^[^ ^17^, ^18^, ^19^ ^]^ However, after 15 h in 1 m KOH, the remaining HMF concentration drops below 40% (as discussed in the Section 2),^[^ ^20^ ^]^ due to undesired polymerization of HMF into humin under strong alkalinity. Furthermore, OH^−^ consumption during HMFOR leads to a local pH decrease near the anode and electrolyte junction.^[^ ^21^ ^]^

Thus, it is essential to investigate HMFOR activity in lower pH electrolyte conditions. However, intermediate aldehydes such as 5‐formyl‐2‐furancarboxylic acid (FFCA) tend to accumulate, and the current density for HMFOR consequently remains low.^[^ ^22^, ^23^ ^]^ This underscores the importance of studying aldehyde oxidation to FDCA. The disproportionation of aldehydes into alcohols and carboxylic acids is accelerated via the Cannizzaro reaction, and geminal diolate formation from aldehydes is more readily promoted by OH^−^. Unlike in strongly alkaline media, the Cannizzaro reaction and geminal diolate formation—both favorable pathways for converting aldehydes to carboxylic acids—may be suppressed under weakly alkaline conditions.^[^ ^24^, ^25^, ^26^, ^27^ ^]^ Therefore, the catalyst should be designed with active sites capable of oxidizing both alcohols and aldehydes.^[^ ^28^, ^29^ ^]^ Bimetallic heterostructures or co‐doping with other elements introduce a diversity of active sites for multi‐step reactions,^[^ ^17^, ^30^, ^31^ ^]^ by tuning the electronic and structural properties.^[^ ^32^, ^33^ ^]^ Furthermore, the electrocatalytic surface must also be tailored to the pH conditions, as aldehyde oxidation in weakly alkaline electrolytes would appear to rely more heavily on adsorbed OH species (^*^OH).^[^ ^34^, ^35^ ^]^ Cu‐based catalysts have been employed for HMFOR due to their inherently low activity for the OER, which suppresses the formation of OER intermediates such as O^*^ and ^*^OOH from ^*^OH.^[^ ^28^, ^36^, ^37^ ^]^ However, mechanistic studies as well as active sites have not been well understood when Cu was included in the electrocatalyst surfaces.

Herein, an interface‐rich CuO@NiOOH electrocatalyst with a CuO core and NiOOH shell was developed for efficient HMFOR under weaker alkaline conditions than pH 14 (pH 12). Unlike CuO and Ni(OH)_2_, which showed reduced FDCA yields, CuO@NiOOH achieved high FDCA yield and Faradaic efficiency across 5–50 mm HMF. A synergistic oxidation mechanism was observed at the interface. The Cu sites enhanced OH affinity to promote aldehyde oxidation, while NiOOH sites accelerated dehydrogenation with favorable H adsorption and reversible Ni(OH)_2_/NiOOH cycling. The selective FDCA production is achieved by favorable direct aldehyde oxidation through carbonyl radical formation on the CuO/NiOOH rather than the geminal‐diolate mechanism, which is more sensitive to OH^−^ concentration. These findings, which account for both catalyst composition and pH‐dependent oxidation reaction pathways, offer valuable insight into biomass oxidation under low‐pH conditions and across diverse electrolyte environments.

## 2 Results and Discussion

### 2.1 HMF Stability in Alkaline Media

**Figure 1:**
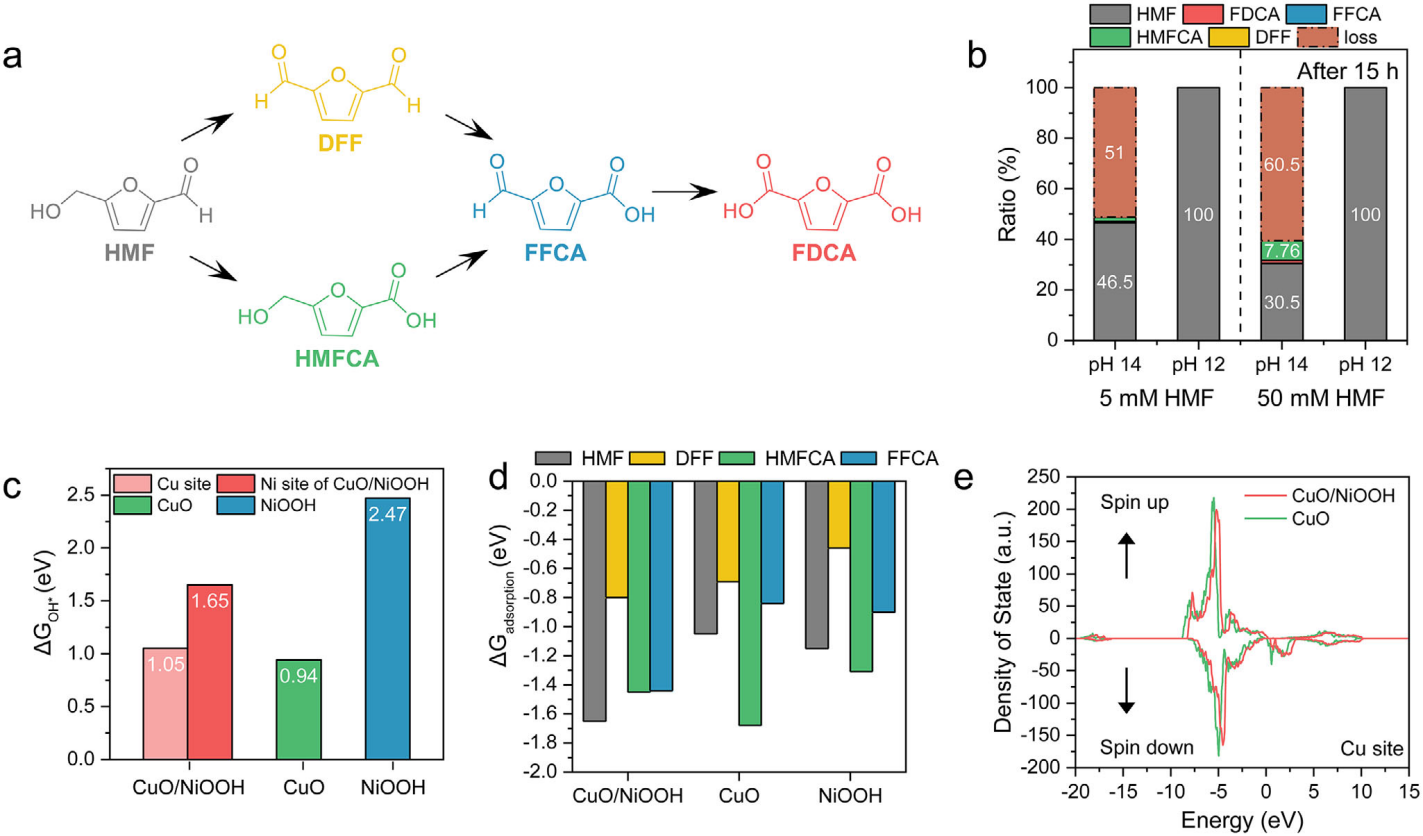
a) Proposed HMFOR pathways, b) Tracking of the organic molecules ratio derived from HMF after 15 h, depending on the different pH conditions and initial HMF concentrations. 1 m KOH and 0.5 m phosphate buffer were used to investigate the pH 14 and pH 12 conditions, respectively. c) Plot of OH adsorption (^*^OH) energy as a function of the surface model (CuO/NiOOH, CuO, NiOOH). d) Plot of organic molecules (HMF, DFF, HMFCA, FFCA) adsorption energy as a function of the surface model (CuO/NiOOH, CuO, NiOOH). e) PDOS calculations for CuO and Cu sites in CuO/NiOOH.

The initial oxidation of HMF, which contains both aldehyde and alcohol functional groups on the furan ring,^[^ ^3^ ^]^ is proposed to proceed via alcohol or aldehyde oxidation to form 2,5‐furandicarboxaldehyde (DFF) and 5‐(hydroxymethyl)furan‐2‐carboxylic acid (HMFCA), respectively (**Figure** **1**a). Both DFF and HMFCA can be further converted to FFCA, and subsequent aldehyde oxidation of FFCA yields FDCA. In addition to oxidation, HMF can decompose or undergo polymerization into humin under strongly alkaline conditions, such as in 1 m KOH, resulting in carbon loss.^[^ ^20^, ^24^ ^]^

To evaluate this, the amount of remaining HMF was compared at pH 12 (0.5 m phosphate buffer, PB) and pH 14 (1 m KOH) at open circuit potential after 15 h (Figure 1b). An initial concentration of 5 mm HMF showed significant carbon loss at pH 14, with the HMF concentration decreasing to 46.5%. Only trace amounts of intermediate molecules were detected, and DFF and FFCA—both containing aldehyde groups—were present at levels below 0.2%, indicating that the Cannizzaro reaction of HMF occurred under strongly alkaline conditions even at room temperature.^[^ ^25^ ^]^ When the HMF concentration was increased to 50 mm, carbon loss rose further to 60.5%, and the HMFCA ratio increased significantly to 7.76%, compared to 1.71% at the 5 mm HMF condition. In contrast, at pH 12, both 5 and 50 mm well‐preserved HMF, confirming that OH^−^‐mediated side reactions are effectively suppressed in mildly alkaline electrolytes. These results demonstrate that pH plays a critical role in concentrated HMFOR electrolysis due to the increased risk of HMF degradation under strongly alkaline conditions. Also, we measured nuclear magnetic resonance spectroscopy (NMR) to investigate the effect of OH^−^ on HMF degradation over 6 h under two different pH conditions. While pH 12 maintained the initial features well, pH 14 exhibited the geminal diol formation as well as formic acid, derived from HMF degradation via a ring‐opening mechanism. This supports that the OH^−^‐ mediated side reactions are effectively suppressed at pH 12 (see Figures S1 and S2, Supporting Information for details).

The pH of the electrolyte influences both product selectivity and current density in HMFOR, as OH^−^ ions participate in catalyst activation, and the oxidation kinetics of alcohol and aldehyde groups differ.^[^ ^38^, ^39^ ^]^ Previous studies have shown that although the HMFCA pathway is favored under high pH conditions (pH ≥ 13), lower pH conditions suppress aldehyde oxidation, suggesting that the DFF pathway may dominate.^[^ ^39^ ^]^ Because the Cannizzaro reaction proceeds via a geminal‐diolate intermediate, geminal diol‐mediated aldehyde oxidation is expected to be more sensitive to pH. Aldehyde oxidation can proceed through two pH‐dependent pathways: geminal diolate‐based oxidation involving external OH^−^, or direct aldehyde oxidation facilitated by adsorbed OH. At pH 12, where the OH^−^ concentration is lower, OH adsorption on catalyst active sites is expected to play a more critical role in HMFOR, highlighting the need to develop electrocatalysts suitable for mildly alkaline conditions.

### 2.2 Theoretical Aspects of Adsorption Behavior on the Surface of Catalysts

To assess oxidation active sites, the adsorption energies of ^*^OH and key intermediate molecules were calculated on CuO and NiOOH using density functional theory (DFT) simulations. The oxidized surfaces of CuO (111), NiOOH (001), and the CuO/NiOOH interface were compared (oxidized surfaces of Cu and Ni, and their interface) to evaluate potential synergistic catalytic effects (Figure S3, Supporting Information). Notably, NiOOH exhibited ^*^OH binding at the µ‐O site with an adsorption energy of +2.47 eV—significantly higher than those on CuO and the CuO/NiOOH interface (Figure 1c). The most stable OH adsorption sites were identified as the Cu–Cu bridge site on CuO and the interfacial oxygen site between Cu and Ni atoms on the CuO/NiOOH interface, with Gibbs free energies of +0.94 and +1.05 eV, respectively. These results demonstrate that Cu‐containing surfaces are more favorable for OH adsorption.

In addition, the adsorption energies of HMF, DFF, HMFCA, and FFCA were compared relative to their gas‐phase energies (Figures 1d; S4, Supporting Information). Among the catalysts, except for HMFCA, all adsorbates exhibited the strongest binding affinities on the CuO/NiOOH interface compared to CuO and NiOOH individually. This is attributed to additional interactions provided by the interfacial sites, as well as modifications in the electronic structure of the Cu sites. Projected density of states (PDOS) calculations for CuO and the Cu sites in CuO/NiOOH show that the d‐band center of Cu in CuO/NiOOH shifts toward the Fermi level by 0.423 eV relative to CuO. This shift enhances bonding strength by raising the energy levels of the adsorbate antibonding states, making them less populated (Figure 1e). In contrast, the Ni sites in CuO/NiOOH do not show a significant shift in the d‐band center, indicating that their electronic properties remain similar to those in NiOOH (Figure S5, Supporting Information).

Based on the theoretical results, CuO/NiOOH is expected to enhance the binding energies of both OH^−^ and organic molecules involved in HMFOR. The ability to attract OH^−^ arises from OH adsorption on the Cu sites, while the strong stabilization of reaction intermediates is attributed to electronic modulation of CuO, influenced by its geometric position at the CuO/NiOOH interface. These combined effects make CuO/NiOOH a potentially more active catalyst than either NiOOH or CuO alone, a synergic effect.

### 2.3 Electrochemical HMFOR Investigation at pH 12

**Figure 2:**
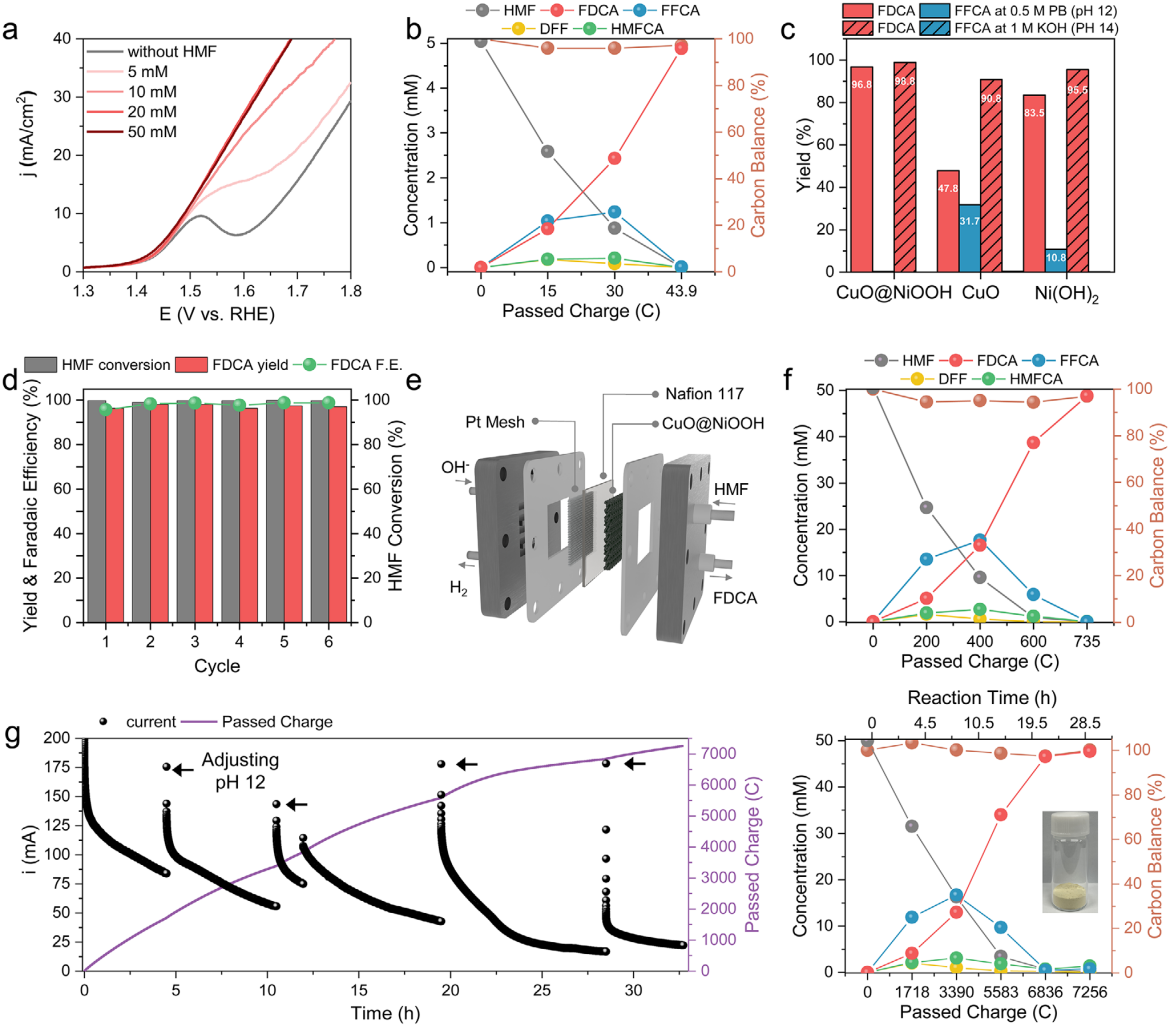
Electrochemical HMFOR performance. a) LSV curves of CuO@NiOOH catalyst depending on the concentration of HMF at pH 12 (0.5 m PB) at a scan rate of 5 mV sec^−1^. b) Concentration changes of HMF and its products versus passed charge during HMFOR with 5 mm HMF at 1.45 V vs RHE using CuO@NiOOH catalyst at pH 12. c) Yield of FDCA and FFCA depending on the catalysts (CuO@NiOOH, CuO, and Ni(OH)_2_) at different electrolyte conditions. HMFOR was conducted via CA at 1.45 V vs RHE at pH 12 (0.5 m PB) and at 1.39 V vs RHE at pH 14 (1 m KOH) with 5 mm HMF. d) HMF conversion, FDCA yield, and FE results for six successive cycles of HMFOR. e) Scheme of MEA design. f) Concentration changes of HMF and its products versus passed charge during HMFOR with 50 mm HMF at 2.0 V using CuO@NiOOH catalyst at pH 12 via MEA electrolyzer. g) CA curve during HMFOR under 50 mm HMF of 250 mL (total 1.58 g of HMF) at 2.0 V vs RHE at pH 12. KOH was added to adjust the pH to 12. h) Concentration of changes of HMF and its products versus passed charges, with a photograph of the FDCA product obtained after long‐term electrolysis.

Motivated by the theoretical results, an interface‐rich CuO/NiOOH electrocatalyst, denoted as CuO@NiOOH, was prepared, and its HMFOR performance was evaluated at pH 12 (0.5 m PB). The detailed synthesis procedure and material characterization used to confirm the interaction between Cu and Ni are discussed later.^[^ ^40^, ^41^ ^]^ Linear sweep voltammetry (LSV) of CuO@NiOOH was conducted across HMF concentrations ranging from 0 to 50 mm (**Figure** **2**a). In the absence of HMF, a typical pre‐oxidation anodic peak corresponding to the Ni(OH)_2_ to NiOOH transition appeared near 1.5 V vs RHE, followed by an increase in the OER. As the HMF concentration increased, this anodic peak became indistinguishable due to the increased HMFOR current densities. In contrast, both CuO and Ni(OH)_2_ exhibited noticeably lower anodic currents, particularly near 1.5 V vs RHE, compared to CuO@NiOOH (Figure S6, Supporting Information). Furthermore, in the case of Ni(OH)_2_, at 50 mm HMF, a diffusion‐limited current region was observed at pH 12, while showing improved mass transport in 1 m KOH (Figure S7, Supporting Information). These results indicate the poor ^*^OH adsorption on Ni(OH)_2_ at pH 12.

The HMFOR performance of CuO@NiOOH was further evaluated at pH 12 using 5 mm HMF by quantifying the HMF‐derived products through high‐performance liquid chromatography (HPLC) (Figures 2b; S8–S10, Supporting Information). As the charge increases, the predominant intermediate, FFCA, disappears, and HMF conversion exceeds 99.9% at 1.45 V vs RHE. CuO@NiOOH exhibited the highest FDCA production rate compared to the control catalysts (Figure S12a, Supporting Information). Simultaneously, high FDCA selectivity was achieved, with an FDCA yield of 96.9% and a Faradaic efficiency (FE) of 96.5% at the terminal charge (43.9C), corresponding closely to the theoretical full oxidation of 5 mm HMF to FDCA. In contrast, the control catalysts, CuO and Ni(OH)_2_, showed lower FDCA yields (CuO: 47.8%; Ni(OH)_2_: 83.5% at 1.45 V vs RHE) (Figures 2c; S10b,c, Supporting Information). Moreover, FFCA yields remained at 31.7% and 10.8% for CuO and Ni(OH)_2_, respectively, indicating incomplete conversion to FDCA even after longer reaction times (i.e., 1650 and 240 min, respectively). A similar trend was observed at 1.5 V vs RHE. The CuO@NiOOH achieves an FDCA yield of 98.6% and FE of 99.2% (Figure S11, Supporting Information). To the best of our knowledge, this is the highest performance at pH 12. Meanwhile, FFCA residues persisted with the control catalysts (CuO: 18.3%; Ni(OH)_2_: 4.5%) (Figure S11b,c, Supporting Information). These findings suggest that the conversion step from FFCA to FDCA is hindered at pH 12. In addition, the FDCA production rate of CuO@NiOOH was also dominated at 1.5 V vs RHE, indicating that not only the high selectivity of FDCA but also the fast kinetics of HMFOR were achieved at pH 12 (Figure S12b, Supporting Information).

To investigate the effect of pH, the catalytic activities were compared in 1 m KOH (Figure 2c). Under these strongly alkaline conditions, all three catalysts achieved FDCA yields above 90% at 1.39 V vs RHE. CuO@NiOOH reached a FDCA yield of 98.8% with a FE of 98.0% (Figure S13, Supporting Information), and FFCA was barely detected in 1 m KOH. These results align with theoretical predictions that CuO@NiOOH, with its stronger affinities for ^*^OH and FFCA, enhances the conversion to FDCA, particularly under weaker alkaline conditions, pH 12.

To confirm the recyclability of catalyst performance, six consecutive cycle tests were conducted at 1.45 V vs RHE using the same CuO@NiOOH catalyst (Figure 2d). FDCA yields remained above 96% with high FE (≥ 95.7%), and similar current densities were maintained throughout successive electrolysis cycles (Figure S14, Supporting Information). Reactivity at pH 12 under concentrated 50 mm HMF was further validated using a membrane electrode assembly (MEA) electrolyzer, paired with the HER at the cathode (Figure 2e). In the presence of 50 mm HMF, higher current densities were observed at the same cell potential due to enhanced HMFOR activity compared to the OER (Figure S15a, Supporting Information). At 2.0 V, the final FDCA yield and FE reached 96.5% and 96.2%, respectively (Figures 2f; S15b,c, Supporting Information).

As the HMF is stable at pH 12, the large volume of electrolysis is also applicable for long‐term operations. Thus, HMFOR was conducted under the concentration of 50 mm HMF in 0.5 m PB with a larger volume of 250 mL (total 1.58 g of HMF), operating the MEA electrolyzer at 2.0 V vs RHE. The initially high current of nearly 150 mA was obtained, whose current decreased over the reaction time due to the consumption of HMR (Figure 2g). The 7256C, which is close to the theoretical charge required for the complete oxidation of HMF to FDCA, was achieved at 32.8 h. As charges passed, the FDCA was accumulated, and the selective FDCA production was also achieved under gram‐scale HMFOR, with FDCA yield of 95.3% and FE of 95.1% (Figures 2h; S16a, Supporting Information). Furthermore, the NMR spectra of the obtained powder after electrolysis show only the features of FDCA, supporting the high selectivity of FDCA production (Figure S16b, Supporting Information). Thus, these results demonstrate the feasibility of the HMFOR system under large‐volume conditions without noticeable HMF degradation.

High yields and efficiencies were also achieved across phosphate buffer concentrations of 0.25, 0.50, and 0.75 m (Figure S17, Supporting Information). Additionally, the HMFOR was measured in other electrolytes near pH 12, such as 0.75 m borate buffer and 1 m K_2_CO_3_ (Figure S18, Supporting Information). The overall yield and FE of FDCA were achieved above 94%, indicating that the highly selective FDCA production was due to the catalyst effect of CuO@NiOOH rather than the anion (phosphate effect). CuO@NiOOH demonstrated particularly superior HMFOR to FDCA at pH 12 over a wide range of HMF concentrations, surpassing previously reported catalysts in both OH^−^‐rich and OH^−^‐deficient media (Table S1, Supporting Information).

### 2.4 Material Characterization of CuO@NiOOH Catalyst

**Figure 3:**
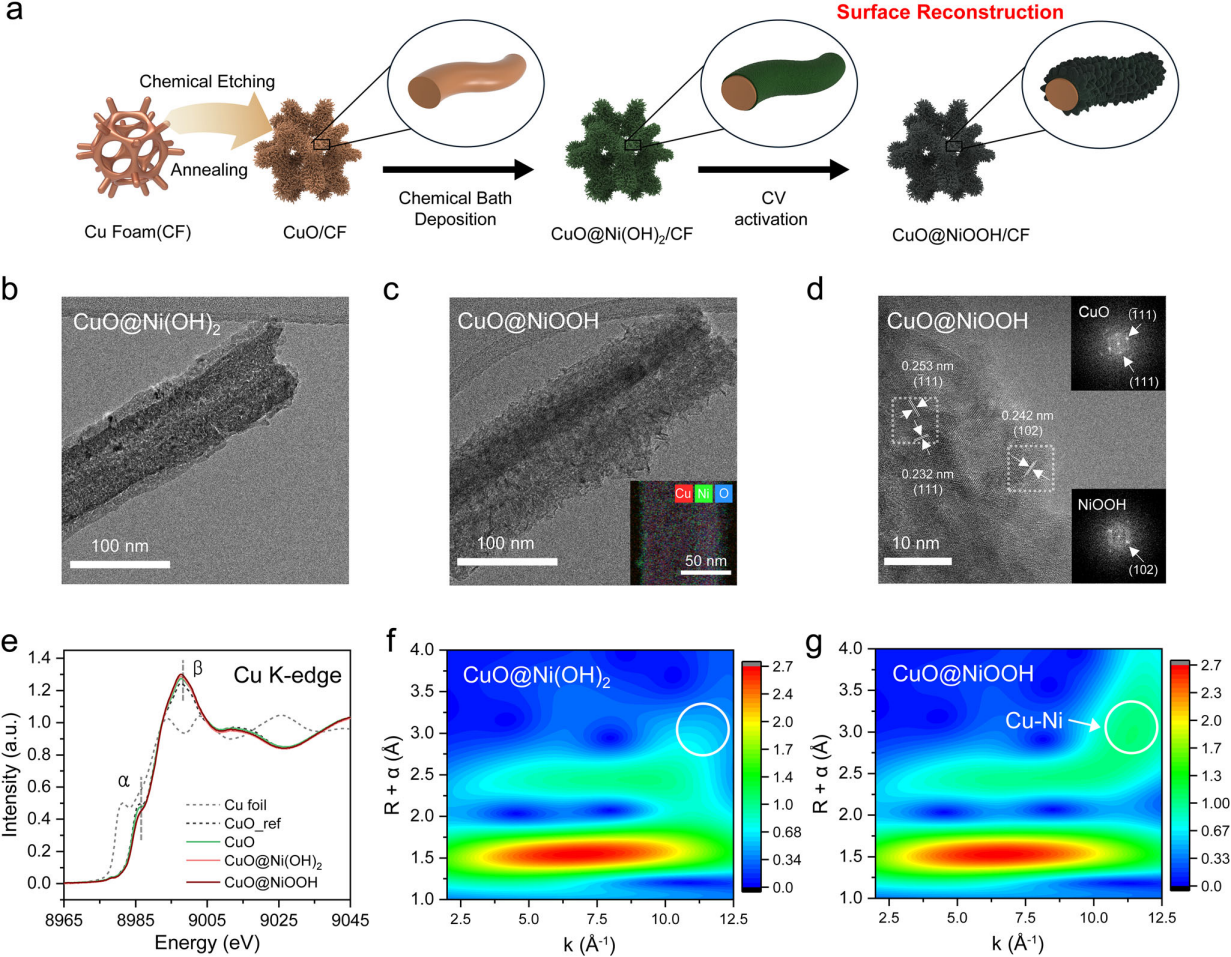
Material Characterization. a) Scheme of Synthesis for CuO@NiOOH. TEM images of b) CuO@Ni(OH)_2_, c) CuO@NiOOH. d) HR‐TEM image of CuO@NiOOH. e) Cu K‐edge XANES of CuO, CuO@Ni(OH)_2_, and CuO@NiOOH. WT‐EXAFS of f) CuO@Ni(OH)_2_, and g) CuO@NiOOH.

To identify its active sites, the CuO@NiOOH catalyst was systematically characterized. The CuO@NiOOH catalysts were prepared according to the outlined procedure (**Figure** **3**a). X‐ray diffraction (XRD) patterns and scanning electron microscopy (SEM) images confirmed the monoclinic CuO nanowire (NW) arrays on Cu foam (CF) (Figures S19 and S20, Supporting Information), and transmission electron microscopy (TEM) revealed ≈100 nm in their widths (Figure S21, Supporting Information). Subsequently, Ni(OH)_2_ was deposited onto the CuO/CF via chemical bath deposition (denoted as CuO@Ni(OH)_2_), and the NW morphology was preserved (Figures 3b; S22a,b, Supporting Information). Although Ni(OH)_2_ features were not significantly obtained in the XRD (Figure S19, Supporting Information), energy dispersive spectroscopy (EDS) of CuO@Ni(OH)_2_ revealed that ≈5 nm thin Ni nanostructures were concentrated on the CuO surface (Figure S23, Supporting Information), and the Ni K‐edge X‐ray absorption near‐edge structure (XANES) spectrum closely resembled that of the Ni(OH)_2_ reference (Figure S24, Supporting Information), suggesting a core‐shell structure with CuO/Ni(OH)_2_ interfaces. Electrochemical cyclic voltammetry (CV) was performed on the CuO@Ni(OH)_2_ precatalyst between 0.77 and 1.97 V vs RHE. The increasing Ni(OH)_2_/NiOOH redox peaks indicated surface reconstruction and exposure of active Ni sites (Figure S25, Supporting Information). After CV activation, the resulting CuO@NiOOH retained its 1D NW morphology (Figure S22c,d, Supporting Information) with rougher sidewalls (Figure 3b,c) and crystalline CuO phase (Figure S19, Supporting Information). Noticeably, XPS analysis showed a high Ni‐to‐Cu ratio, a Ni‐rich surface (Figure S26, Supporting Information), and the lattice fringes in HR‐TEM images revealed the coexistence of NiOOH nanoparticles with monoclinic CuO (Figure 3d). Collectively, these results suggest that the interfacial CuO/NiOOH sites became more exposed through reconstruction. Although there are structural deviations between our nanocatalyst and the flat model of the DFT simulation, the formation of the interface between CuO and NiOOH is confirmed on the nanowire surface.

Next, Cu K‐edge X‐ray absorption spectroscopy (XAS) was performed on CuO@Ni(OH)_2_ an CuO@NiOOH to gain deeper insight into their bonding properties (Figure 3e–g). The XANES spectra of CuO, CuO@Ni(OH)_2_, and CuO@NiOOH showed characteristic CuO features, consistent with TEM and XRD. Compared to pure CuO, the composites exhibited diminished α peaks and slightly enhanced β peaks (Figure 3e). Since these peaks correspond to Cu 1s (involving O shakedown or Cu transitions) excitations to Cu 4p–O 2p antibonding orbitals, the subtle changes indicate that the Cu local coordination is slightly altered by interactions with Ni(OH)_2_ or NiOOH.^[^ ^42^, ^43^ ^]^ Additionally, the Cu–O and Cu–Cu coordination peaks were compared using extended X‐ray absorption fine structure (EXAFS) analysis (Figures S27, Supporting Information). In particular, Morlet wavelet‐transformed EXAFS (WT‐EXAFS) revealed a distinct Cu–O lobe at (6.5 Å^−1^, 1.5 Å) corresponding to the first coordination shell, as well as a Cu–O lobe at (3.7 Å^−1^, 2.4 Å) and two Cu–Cu lobes at (6.5 Å^−1^, 2.4 Å) and (10.4 Å^−1^, 2.7 Å) associated with the second shell in both CuO and CuO@Ni(OH)_2_ (Figure S28, Supporting Information; Figure 3f).^[^ ^44^ ^]^ Notably, a new lobe at (11.6 Å^−1^, 3.0 Å) emerged in CuO@NiOOH (Figure 3g). Given the observed phase transformation to NiOOH on the surface during CV activation, this lobe is attributed to the Cu─O─Ni bonds at the CuO/NiOOH interface, indicating enhanced interfacial coupling.^[^ ^45^, ^46^, ^47^ ^]^ Moreover, Cu 2p XPS analysis of CuO@NiOOH revealed more increased Cu^2+^ oxidation state (Figure S29a, Supporting Information), compared to CuO backbone (Figure S29b, Supporting Information).^[^ ^48^ ^]^ In contrast, the Ni^3+^/Ni^2+^ ratio in CuO@Ni(OH)_2_ decreased (Figure S30, Supporting Information).^[^ ^49^ ^]^ Considering the Cu K‐edge hard XANES results, which show similar edge energies for CuO@Ni(OH)_2_ and CuO@NiOOH, these findings suggest that electronic modulation occurs near the surface. It probably involves electron transfer from Cu to Ni species as a result of CuO/NiOOH interface formation.

In contrast to CuO@NiOOH, Ni(OH)_2_ exhibited no significant changes after CV activation. A Ni(OH)_2_ control catalyst was also prepared, and the XRD patterns (Figure S31, Supporting Information) and Ni K‐edge XANES (Figure S24, Supporting Information) confirmed the presence of the β‐Ni(OH)_2_ phase, and the SEM image revealed a flake‐like morphology (Figure S32, Supporting Information). Moreover, the overall material characteristics remained consistent after HMFOR (Figures S33–S37, Supporting Information).

In addition, post‐reaction characterization of the CuO@NiOOH catalyst after the long‐term stability test was performed (Figure 2g,h). The nanowire structure was well preserved, and the characteristic CuO/NiOOH interfacial features remained intact, confirming the robust structural stability of the CuO@NiOOH catalyst (Figures S38 and S39, Supporting Information).

### 2.5 Changes in Interfacial Active Sites During HMFOR

**Figure 4:**
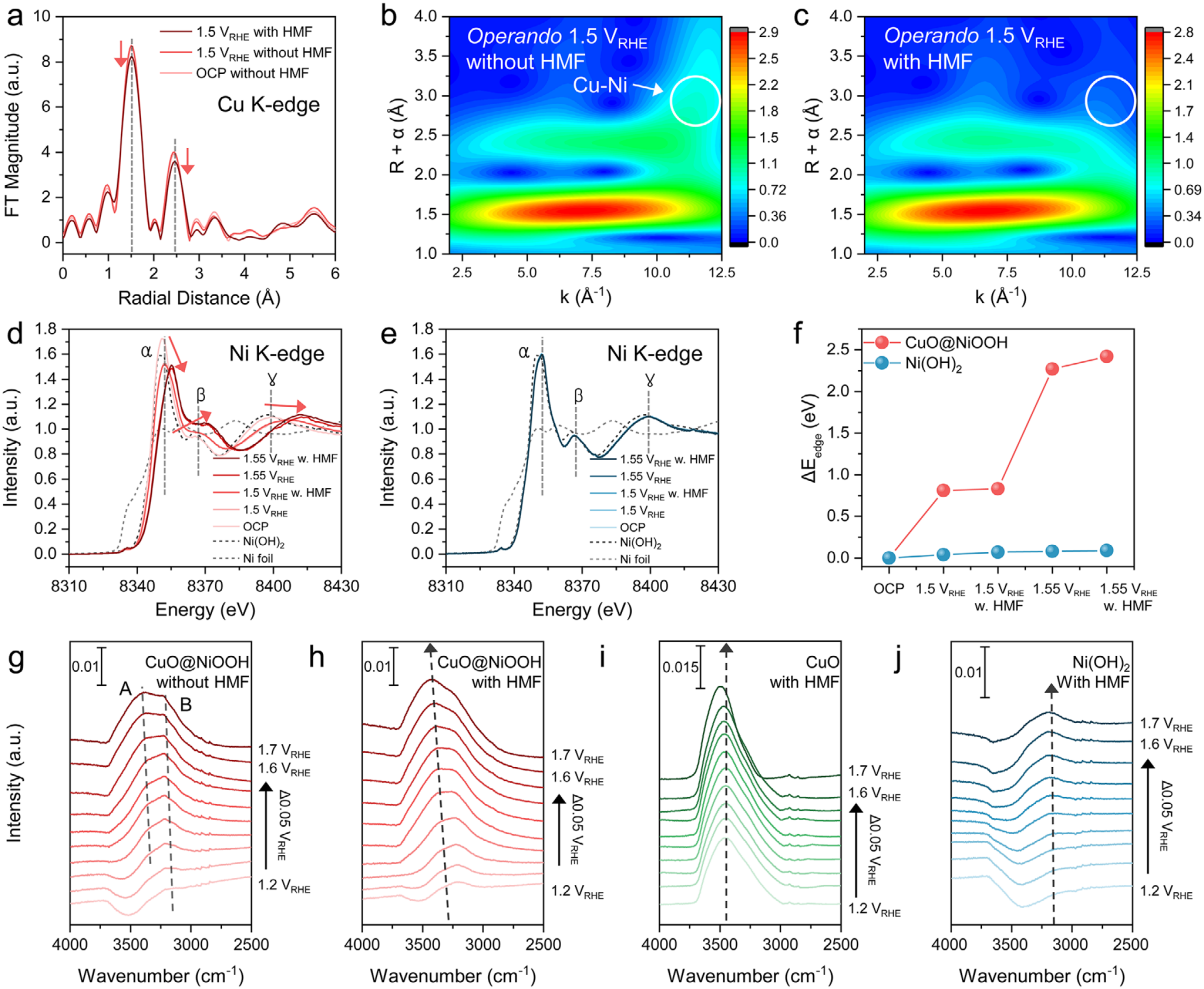
*Operando* spectroscopic results. a) EXAFS of Cu K‐edge for in situ*/operando* electrolysis using CuO@NiOOH at pH 12. *Operando* WT‐EXAFS of Cu K‐edge using CuO@NiOOH during CA at 1.5 V vs RHE b) without HMF, and c) with 5 mm HMF. in situ*/operando* XANES of Ni K‐edge at pH 12 without/with 5 mm HMF using d) CuO@NiOOH, and e) Ni(OH)_2_. f) Plot of ΔE_edge_ vs *operando* condition for CuO@NiOOH, and Ni(OH)_2_ based on the Ni K‐edge XANES. ΔE_edge_ is the difference between the edge energy of the OCP condition and the applied potential condition. g) *Operando* ATR‐SEIRAS of CuO@NiOOH at pH 12 without HMF during applied potential. *Operando* ATR‐SEIRAS for HMFOR with 5 mm HMF at pH 12 using h) CuO@NiOOH, i) CuO, and j) Ni(OH)_2_.

**Figure 5:**
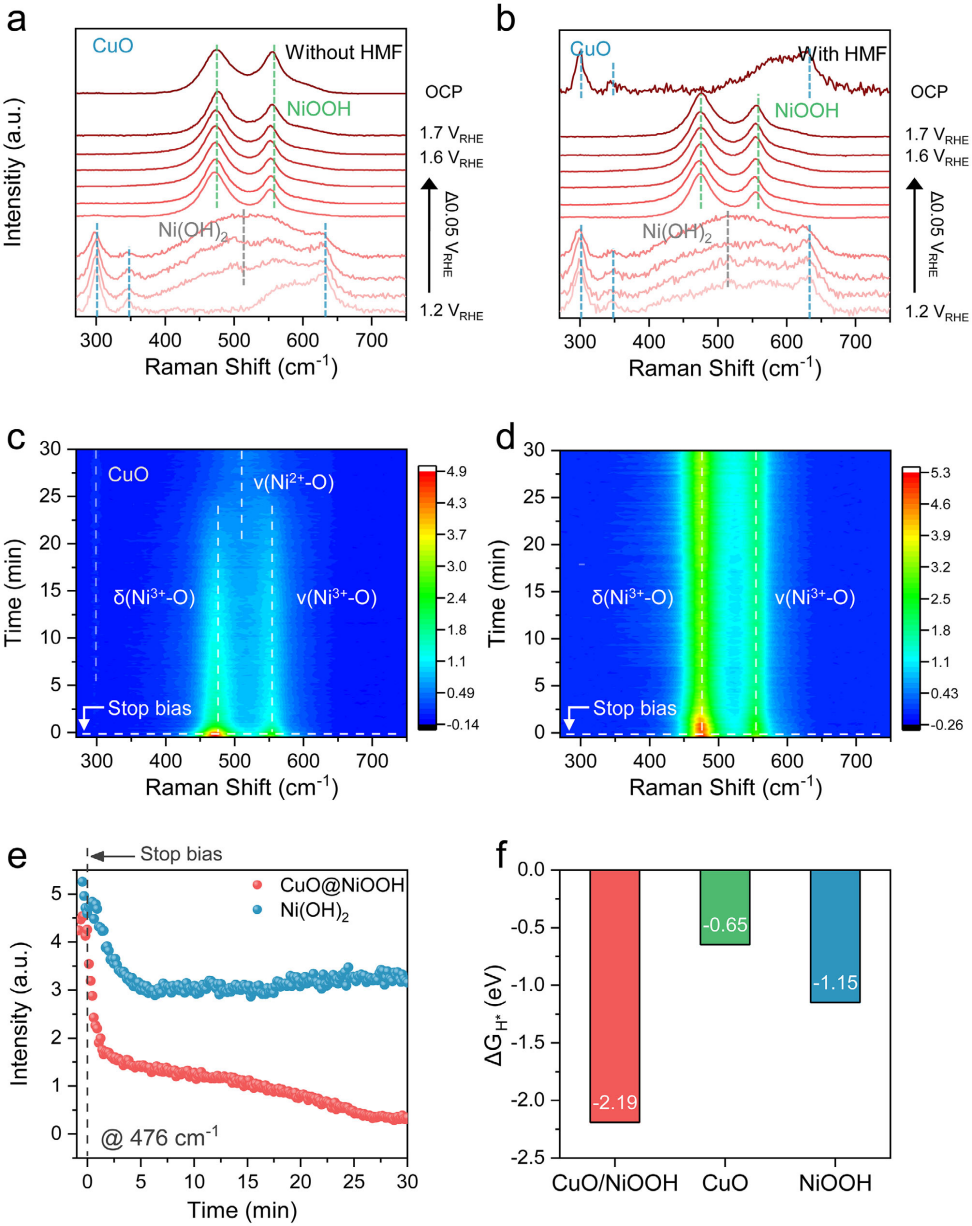
*Operando* Raman spectroscopy of CuO@NiOOH, a) without HMF and b) with 50 mm HMF conditions at pH 12. TRS results at OCP condition after stopping bias at 1.45 V vs RHE with 50 mm HMF using c) CuO@NiOOH, and d) Ni(OH)_2_. e) Plot of intensities of the peak at 476 cm^−1^ versus time during TRS for CuO@NiOOH and Ni(OH)_2_. f) Plot of H adsorption (H^*^) energy as a function of the surface model (CuO/NiOOH, CuO, NiOOH).

The electronic and structural states of Cu and Ni were analyzed using *operando* spectroscopies (Figures 4 and 5). In situ/*operando* XAS at the Cu K‐edge was conducted at open circuit potential (OCP) and under an applied potential of 1.5 V vs RHE (denoted as V_RHE_). *Operando* XANES showed that the observed oxidation state of Cu in CuO@NiOOH remained similar between OCP and 1.5 V_RHE_, both with and without HMF conditions (Figure S40, Supporting Information), and EXAFS confirmed the characteristic peaks associated with CuO (**Figure** **4**a). However, at 1.5 V_RHE_ in the presence of HMF, the intensity of CuO features decreased. In contrast, no such reduction in Cu features was observed in the EXAFS analysis of bare CuO (Figure S41, Supporting Information). WT‐EXAFS of CuO@NiOOH showed distinct CuO lobes and a Cu–Ni lobe (highlighted with a white circle) at both OCP and 1.5 V_RHE_ without 5 mm HMF (Figure S42, Supporting Information; Figure 4b). Notably, upon introducing 5 mm HMF at 1.5 V_RHE_, the Cu–(O)–Ni lobe disappeared (Figure 4c). This disappearance suggests that the Cu─O─Ni bonding at the CuO/NiOOH interface serves as the dynamic active site and is weakened during HMFOR. This finding aligns with DFT calculations, which show enhanced adsorption of OH and HMF at the CuO/NiOOH interface compared to CuO or NiOOH alone. Specifically, OH, HMF adsorption, or H adaptation of the interface from organic molecules via dehydrogenation would contribute to a local structural change, leading to an increased distance between CuO and NiOOH at the interface. In contrast, WT‐EXAFS analysis of CuO showed no change, indicating that its structure remained stable during HMFOR (Figure S43, Supporting Information).

In addition, the Ni K‐edge XAS analysis was performed to investigate the behavior of the Ni(OH)_2_/Ni(OH)O domains during HMFOR (Figure 4d). The Ni K‐edge XANES revealed that CuO@NiOOH exhibited characteristic features of the Ni(OH)_2_ phase at OCP. Upon applying an anodic potential, the α peak shifted from 8351 eV to a higher energy of 8355 eV at 1.55 V vs RHE, accompanied by a decrease in intensity. Simultaneously, the β and γ peaks shifted from 8366 and 8398 eV to 8371 and 8412 eV, respectively, as the potential increased from OCP to 1.55 V vs RHE. These XANES changes indicate the evolution of NiOOH through oxidation of Ni(OH)_2_.^[^ ^50^ ^]^ In contrast, the XANES of the Ni(OH)_2_ control catalyst consistently showed features of the Ni(OH)_2_ phase under all conditions (Figure 4e), with a similar edge energy shift (ΔE_edge_) but, CuO@NiOOH exhibited a significantly larger ΔE_edge_ of up to 2.42 eV, indicating a higher Ni oxidation state under anodic potential (Figures 4f; S44, Supporting Information). Linear combination fitting (LCF) further confirmed that the conversion of Ni(OH)_2_ to NiOOH was more favorable in CuO@NiOOH under anodic bias, as evidenced by the increased NiOOH/Ni(OH)_2_ ratio (see Figures S45–S48 and Table S2, Supporting Information for details).^[^ ^51^ ^]^

According to DFT simulations, the ^*^OH affinity of CuO@NiOOH was higher than that of Ni(OH)_2_, facilitating the conversion of Ni(OH)_2_ to NiOOH because the OH^−^ is involved in the following reaction: Ni(OH)_2_ + OH^−^ → NiOOH + H_2_O. TEM images revealed the formation of a CuO/NiOOH interface on the surface of CuO with a small grain size, proposing the promoted oxidation of Ni(OH)_2_ to NiOOH by ^*^OH adsorption, even under low pH conditions. Notably, NiOOH formation precedes the dehydrogenation step of organic molecules during HMFOR, and the rapid reversibility of the Ni(OH)_2_/NiOOH phase in CuO@NiOOH may contribute to efficient HMFOR.

In addition to the electronic changes on the catalyst surface, *operando* attenuated total reflectance surface‐enhanced infrared absorption spectroscopy (ATR‐SEIRAS) was employed to monitor the chemical species and microenvironment near the electrode–electrolyte interface during HMFOR (Figure 4g–j). For CuO@NiOOH, two distinct features were observed; 3600–3000 cm^−1^ (*v*(O–H) stretching) and 1700–1600 cm^−1^ (H_2_O bending), reflecting the interfacial water structure (Figure S49a,b, Supporting Information).^[^ ^52^ ^]^ As the applied potential increased from 1.2 to 1.7 V_RHE_, the *v*(O–H) peaks intensified and shifted to higher wavenumbers (blue shift) (Figure 4g,h). A higher *ν*(O–H) wavenumber indicates weaker intermolecular interactions of OH^−^ (or H_2_O); thus, *ν*(O–H) features provide insight into the water structure near the catalyst–electrolyte interface, which can influence hydrogen/proton transfer kinetics.^[^ ^53^ ^]^ Above 1.55 V_RHE_, the A peak became dominant. An enhanced A peak suggests a higher proportion of weakly or free‐bound *ν*(O–H) compared to strongly bound *ν*(O–H) (B peak) in the water network. As applying higher potential where OER is active (Figure 4g), the catalyst surface consumes OH^−^ and produces O_2_ and H_2_O, thereby modifying the water network at the electrode–electrolyte interface to favor higher *ν*(O–H) wavenumbers.^[^ ^54^ ^]^

Interestingly, the A peak was more prominent in the presence of 5 mm HMF (Figure 4h), due to the H_2_O released during the conversion of Ni(OH)_2_ to NiOOH, which originates from the dehydrogenation of HMF during HMFOR and OH^−^ consumption via aldehyde oxidation to carboxylic acid. The enhanced ^*^OH affinity of CuO@NiOOH is expected to promote ^*^OH consumption during HMFOR and more effectively modulate the interfacial water network compared to CuO and Ni(OH)_2_ catalysts (Figure 4i,j).^[^ ^17^, ^55^ ^]^ The ATR‐SEIRAS spectra of CuO and Ni(OH)_2_ also exhibited distinct features in the *ν*(O–H) stretching and H_2_O bending modes (Figures S50 and S51, Supporting Information). For CuO, the *v*(O–H) band appeared with strong intensity starting at 1.2 V_RHE_, but its wavenumber remained unchanged with increasing potential. These results indicate that while CuO has an effective affinity for ^*^OH, the utilization of ^*^OH can be limited by the low adsorption energy of HMF. In contrast to CuO and CuO@NiOOH, Ni(OH)_2_ showed only a slight increase in *v*(O–H) intensity beyond 1.45 V_RHE_, consistent with its weaker ^*^OH affinity compared to Cu‐containing catalysts.

When the reaction intermediates of HMFOR were monitored in the range of 1600–1200 cm^−1^, a distinct peak attributed to the carboxyl group of FDCA and FFCA appeared near 1355 cm^−1^ only in the presence of HMF (Figure S49d, Supporting Information), supporting HMFOR activity on the catalyst surface.^[^ ^56^ ^]^ CuO and Ni(OH)_2_ also exhibited peaks at 1355 cm^−1^, corresponding to the carboxyl group, but only from 1.5 V_RHE_ (Figures S50b and S51b, Supporting Information), which is a more anodic potential than that of CuO@NiOOH (1.3 V_RHE_). These SEIRAS features further indicate that the CuO@NiOOH surface provides more favorable adsorption of HMF and its intermediates.

Additionally, to track the structural conversion of the Ni sites between Ni(OH)_2_/NiOOH and their contribution to the HMFOR activity, we performed *operando* Raman spectroscopy analysis. The H‐adsorption energies were also compared to understand the different structural reversibility of NiOOH to Ni(OH)_2_ (**Figure** **5**). First, the Raman spectra were obtained from 1.2 to 1.7 V to include the potential of Ni(OH)_2_/NiOOH oxidation, and HMFOR.

At 1.2 V without HMF, the peaks were observed at 298, 345, and 632 cm^−1^, associated with the A_g_ and two B_g_ modes of CuO, respectively (Figure 5a).^[^ ^57^ ^]^ As the applied potential increased, a broad peak evolved at 510 cm^−1^, attributed to the distorted Ni(OH)_2_ (ν(Ni^2+^‐O)).^[^ ^58^ ^]^ Beyond 1.4 V, distinctive NiOOH features were observed at 476 and 555 cm^−1^, associated with E_g_ (δ(Ni^3+^‐O)) and A_1g_ (ν(Ni^3+^‐O)), consistent with *operando* XAS results.^[^ ^19^, ^58^, ^59^ ^]^ With 50 mm of HMF, similar Raman spectra were observed between 1.2 and 1.7 V_RHE_ (Figure 5b). Notably, in the presence of HMF, the NiOOH peaks diminished sharply, and CuO features reappeared at OCP, supporting the dehydrogenation process of HMFOR reduces NiOOH back to Ni(OH)_2_.^[^ ^60^ ^]^ In contrast, the Ni(OH)_2_ control catalyst exhibited the evolution of two NiOOH peaks at potentials as low as 1.2 V, persisting them at the OCP in the presence of 50 mm HMF (Figure S52, Supporting Information). This surface‐sensitive Raman analysis shows that the NiOOH formed on the surface of the bare Ni(OH)_2_ catalyst has poor reversibility from NiOOH to Ni(OH)_2_ via indirect oxidation compared with CuO@NiOOH.

We conducted time‐resolved Raman spectroscopy (TRS) to investigate the dehydrogenation activity during the indirect oxidation of HMF.^[^ ^61^, ^62^ ^]^ First, CA at 1.45 V_RHE_ was conducted for 30 min with 50 mm HMF to induce the transition from Ni(OH)_2_ to NiOOH, and then Raman spectra were captured at OCP at 10 s intervals. During the TRS, the NiOOH peaks of the CuO@NiOOH catalyst decreased more dramatically than those of Ni(OH)_2_ (Figure 5c,d). Specifically, the peaks at 476 and 554 cm^−1^ from NiOOH almost disappeared after 20 min (Figure S53a, Supporting Information). In contrast, the Ni(OH)_2_ catalyst maintains the prominent features of NiOOH for more than 30 min (Figures 5d; S53b, Supporting Information). Figure 5e compares the intensity changes of the most featured 476 cm^−1^ peak, illustrating that for the Ni(OH)_2_ catalyst, the feature was relatively stable over 30 min, whereas for the CuO@NiOOH catalyst, it dropped sharply within 2 min and nearly disappeared.

The rapid phase transition of NiOOH can be attributed to the high adsorption of HMF and H (Figures 1d and 5c) at the CuO/NiOOH interface. Furthermore, the facile ^*^OH consumption on the CuO@NiOOH catalyst (Figure 1c) and the oxidative phase transition from Ni(OH)_2_ to NiOOH would also play a role in the promotion of the reversibility of Ni(OH)_2_/NiOOH and HMFOR kinetics.

To understand the distinct properties of the phase transition between NiOOH/Ni(OH)_2_, we calculated Gibbs free energies of H adsorption (ΔG_H*_) on the CuO/NiOOH interface, CuO, and NiOOH surface, respectively (Figure 5f). The CuO/NiOOH interface surface exhibited much lower ΔG_H*_, −2.19 eV, compared to NiOOH (−1.15 eV) or CuO (−0.65 eV), implying a synergistic effect on stabilizing H adsorption. Furthermore, through a comparison of the H adsorption between the Cu and Ni sites at the CuO/NiOOH interface (Figure S54, Supporting Information), it can be seen that Ni sites (−2.19 eV) have a higher affinity than the Cu sites (−1.42 eV). Favorable H adsorption on the Ni sites of the CuO/NiOOH interface is consistent with the fast phase transition from NiOOH to Ni(OH)_2_ observed for the CuO@NiOOH catalyst, indicating that the Ni sites act as hydrogen acceptors during the dehydrogenation of HMFOR.

### 2.6 Enhanced Active Factor of HMFOR at pH 12

**Figure 6:**
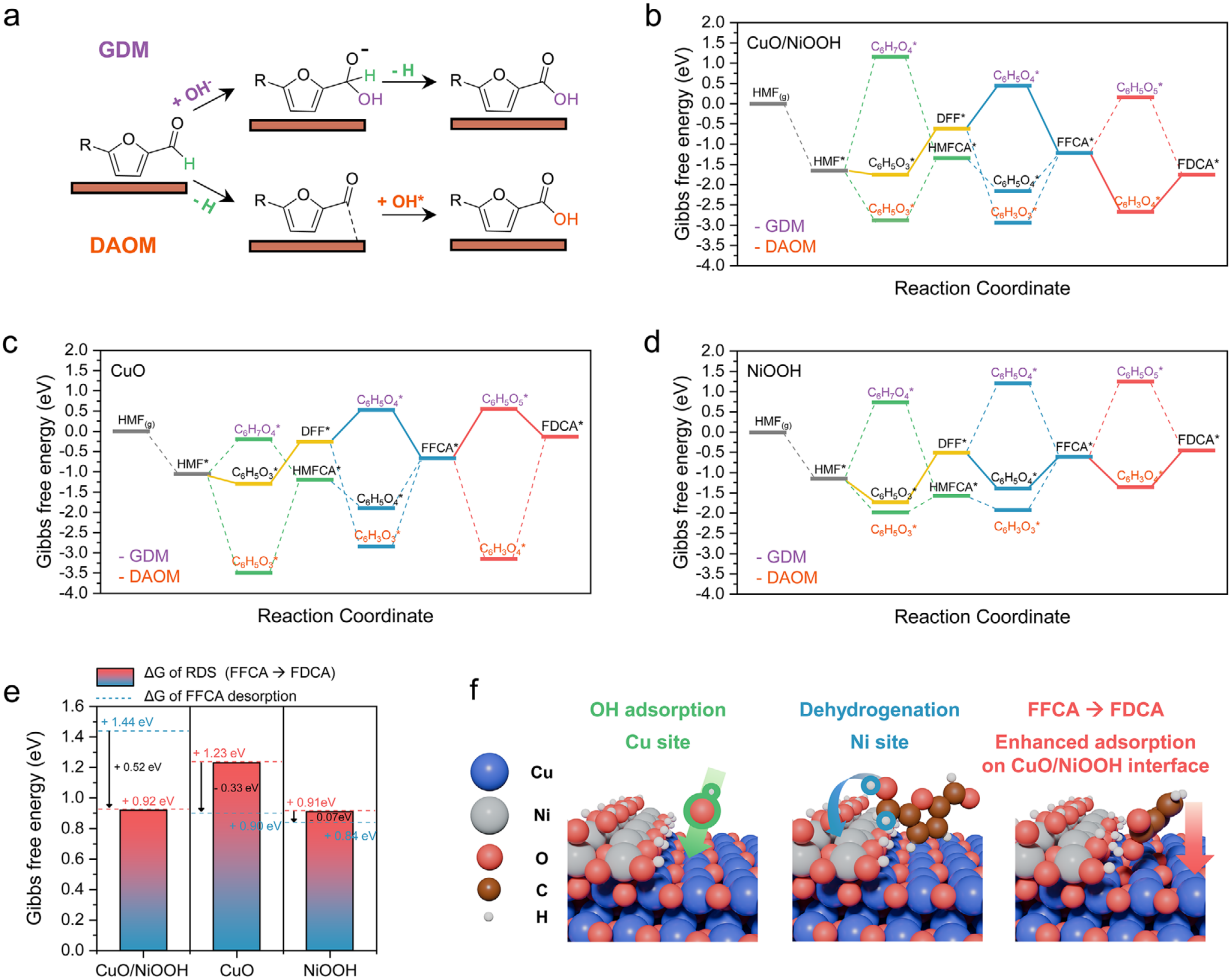
Theoretical analysis of HMF oxidation. a) Schematic illustration of the HMF oxidation reaction pathway. b) Gibbs free energy diagram of HMF oxidation on CuO@NiOOH. c) Gibbs free energy diagram of HMF oxidation on CuO. d) Gibbs free energy diagram of HMF oxidation on NiOOH. e) Gibbs free energies of FFCA desorption and the potential‐determining step (PDS) of FFCA to FDCA formation over CuO/NiOOH, CuO, and NiOOH. f) Schematic illustration of the role of Cu and Ni sites in CuO/NiOOH during the HMF oxidation reaction.

To investigate the HMFOR mechanism of CuO@NiOOH, DFT calculations were performed, considering ^*^OH, H^*^, and intermediate species. Two distinct aldehyde oxidation mechanisms were evaluated: one involving the formation of a geminal‐diolate ion (geminal‐diolate mechanism, GDM), and the other a direct aldehyde oxidation mechanism (DAOM), which produces carbon radicals stabilized by neighboring lattice oxygen (**Figure** **6**a).^[^ ^56^, ^63^, ^64^ ^]^ Figure 6b–d present the free energy diagrams for both GDM and DAOM pathways in HMF oxidation over CuO/NiOOH, depicting the interfacial site of CuO@NiOOH, NiOOH (001), and CuO (111). The most favorable reaction pathway is indicated by a solid line, and the less favorable pathway by a dashed line.

CuO/NiOOH and NiOOH preferentially followed the pathway leading to DFF and subsequently to FFCA, rather than the route involving HMFCA to FFCA. For CuO/NiOOH, the highest endergonic elementary step in the HMF → DFF → FFCA → FDCA pathway is the conversion of HMF to DFF, with an energy barrier of 1.13 eV (Figure 6b). The most energy‐demanding step involving HMFCA is the conversion of HMF to HMFCA, which has an energy barrier of 1.54 eV (Table S3, Supporting Information). In comparison to CuO/NiOOH, NiOOH exhibits a lower barrier for the formation of HMFCA from HMF, with an energy barrier of 0.41 eV via DAOM (Figure 6d; Table S3, Supporting Information), but the DFF route remains the most promising because the free energy change associated with the conversion of HMFCA to FFCA presents the largest barrier (1.31 eV). On the other hand, CuO exhibits the most energy‐demanding step within the entire reaction pathway, which is the conversion of FFCA → FDCA via GDM, with an energy barrier of 1.23 eV, thereby hindering the formation of FDCA (Figure 6c; Table S3, Supporting Information). Moreover, CuO follows only the GDM mechanism rather than the DAOM mechanism throughout the reaction pathway because of the excessive binding strength between the carbon radical and the lattice oxygen. This strong interaction increases the energy barrier for OH^−^ to attack the carbon while simultaneously breaking the bond between the carbon radical and the lattice oxygen. Consequently, the increased energy barrier for breaking this strong bond ensured that GDM consistently exhibited lower energy barriers than DAOM in CuO. In contrast, both CuO/NiOOH and NiOOH demonstrate relatively lower barriers for the FFCA → FDCA conversion, with values of 0.92 and 0.91 eV, respectively. Moreover, DAOM is the preferred mechanism for this specific conversion in both catalysts because it optimally balances the binding strength between the carbon radical and lattice oxygen.

Next, the change in free energy associated with the highest barrier in the FDCA conversion for CuO/NiOOH, CuO, and NiOOH was compared with the free energy of FFCA desorption (Figure 6e) to further understand the product distribution of each catalyst during HMFOR obtained in Figure 2c. The difference between the desorption free energy of FFCA and the highest energy barrier for FDCA formation served as a representative value, indicating whether the catalyst favors early desorption as an intermediate product or the progression to complete oxidation. The calculated desorption free energies of FFCA on CuO/NiOOH, CuO, and NiOOH are 1.44, 0.90, and 0.84 eV, respectively, indicating the strongest binding affinity of FFCA on CuO/NiOOH. The calculated differences between the desorption free energy of FFCA and the highest energy barrier for the FFCA → FDCA conversion are +0.52, −0.33, and −0.07 eV for CuO/NiOOH, CuO, and NiOOH, respectively, indicating that the desorption of early‐stage FFCA intermediates is more feasible on CuO or NiOOH. Therefore, the predicted product selectivity followed the trend of decreasing FFCA selectivity and increasing FDCA selectivity in the order CuO/NiOOH > NiOOH > CuO, and these results are well matched with the experimental tendencies.

Overall, in high pH environments, geminal diolate formation is dominated due to the abundant OH^−^. Thus, both GDM and DAOM proceeded actively. As the pH decreased, the formation of geminal diolates, facilitated by bulk OH^−^ became unfavorable, consequently suppressing the overall oxidation reaction. As a result, significantly reduced reactivity of CuO was observed from 1 m KOH to pH 12 (Figure 2c). In contrast, in the presence of Ni, the oxidation reaction rate and selectivity toward FDCA were less suppressed, which can be attributed to the oxidation proceeding via DAOM under low‐pH conditions. However, because adsorbed OH is involved in oxidation, the low OH adsorption ability of the Ni(OH)_2_ catalyst decreases HMFOR at pH 12. For CuO@NiOOH, FFCA desorption is effectively suppressed owing to its high adsorption energy (Figure 6e), and the enhanced OH adsorption promotes the oxidation of FFCA to FDCA at the CuO/NiOOH interface (Figure 6f).

## 3 Conclusion

This study highlights the importance of investigating electrocatalysts under weakly alkaline conditions to overcome HMF decomposition issues in strongly alkaline media. However, limited OH^−^ concentrations affect the mechanism and restrict HMFOR performance. To address this, an interfacial CuO/NiOOH catalyst (CuO@NiOOH) was designed, through a direct aldehyde oxidation pathway, which achieves an FDCA yield and FE exceeding 95% at pH 12, for the first time. By contrast, while CuO and Ni(OH)_2_ catalysts delivered >90% yields in 1 m KOH, their performance dropped significantly to 47.8% and 83.5%, respectively, at pH 12. By utilizing the enhanced stability of HMF at pH 12, a large‐volume HMFOR electrolysis was operated stably for 32.8 h, resulting in the selective production of FDCA. The CuO/NiOOH interface plays a synergistic role in selective FDCA production by accelerating OH adsorption at the Cu site and enhancing ^*^OH utilization, thereby sustaining HMFOR under low OH^−^ availability. In situ studies revealed that CuO/NiOOH interfacial sites dynamically evolve with HMFOR, while the Ni site exhibits strong hydrogen affinity that promotes efficient oxidation through reversible Ni(OH)_2_/NiOOH cycling. This synergy ensures an optimal balance between FFCA desorption and the potential‐determining step toward FDCA. Overall, this work provides a foundation for designing catalysts enabling selective biomass valorization across broad pH ranges.

## Conflict of Interest

The authors declare no conflict of interest.

## Supporting information



Supporting Information

## Data Availability

The data that support the findings of this study are available from the corresponding author upon reasonable request.

## References

[1] P. Gallezot , Chem. Soc. Rev. 2012, 41, 1538. 21909591 10.1039/c1cs15147a

[2] H. Luo , J. Barrio , N. Sunny , A. Li , L. Steier , N. Shah , I. E. L. Stephens , M. M. Titirici , Adv. Energy Mater. 2021, 11, 2101180.

[3] K. R. Vuyyuru , P. Strasser , Catal. Today 2012, 195, 144.

[4] F. Olivito , V. Algieri , M. A. Tallarida , A. Jiritano , P. Costanzo , L. Maiuolo , A. De Nino , Green Chem. 2023, 25, 1679. 10.3390/polym15071785 PMC10098679 37050399

[5] X. Wan , C. Zhou , J. Chen , W. Deng , Q. Zhang , Y. Yang , Y. Wang , ACS Catal. 2014, 4, 2175.

[6] J. M. Molinaro , J. Swartzentruber , V. W. Ledger , Z. T. Fredericks , D. M. Alonso , S. G. Wettstein , EES Catal. 2025, 3, 595.

[7] E. de Jong , H. R. A. Visser , A. S. Dias , C. Harvey , G. M. Gruter , Polymers 2022, 14, 943. 35267764 10.3390/polym14050943 PMC8912366

[8] Y. Kwon , K. J. P. Schouten , J. C. van der Waal , E. de Jong , M. T. Koper , ACS Catal. 2016, 6, 6704.

[9] Y. Xie , Z. Zhou , N. Yang , G. Zhao , Adv. Funct. Mater. 2021, 31, 2102886.

[10] J. Na , B. Seo , J. Kim , C. W. Lee , H. Lee , Y. J. Hwang , B. K. Min , D. K. Lee , H. S. Oh , U. Lee , Nat. Commun. 2019, 10, 5193. 31729357 10.1038/s41467-019-12744-y PMC6858374

[11] B. Zhou , Y. Li , Y. Zou , W. Chen , W. Zhou , M. Song , Y. Wu , Y. Lu , J. Liu , Y. Wang , S. Wang , Angew. Chem., Int. Ed. 2021, 60, 22908. 10.1002/anie.202109211 34405508

[12] C. Liu , X. R. Shi , K. Yue , P. Wang , K. Zhan , X. Wang , B. Y. Xia , Y. Yan , Adv. Mater. 2023, 35, 2211177. 10.1002/adma.202211177 36606317

[13] B. J. Taitt , D.‐H. Nam , K.‐S. Choi , ACS Catal. 2018, 9, 660.

[14] M. Fleischmann , K. Korinek , D. Pletcher , J. Electroanal. Chem. Interfacial Electrochem. 1971, 31, 39.

[15] W. Chen , C. Xie , Y. Wang , Y. Zou , C.‐L. Dong , Y.‐C. Huang , Z. Xiao , Z. Wei , S. Du , C. Chen , B. Zhou , J. Ma , S. Wang , Chem 2020, 6, 2974.

[16] M. T. Bender , Y. C. Lam , S. Hammes‐Schiffer , K. S. Choi , J. Am. Chem. Soc. 2020, 142, 21538. 33320654 10.1021/jacs.0c10924

[17] P. Zhou , X. Lv , S. Tao , J. Wu , H. Wang , X. Wei , T. Wang , B. Zhou , Y. Lu , T. Frauenheim , X. Fu , S. Wang , Y. Zou , Adv. Mater. 2022, 34, 2204089. 10.1002/adma.202204089 36036562

[18] D. Xiao , X. Bao , D. Dai , Y. Gao , S. Si , Z. Wang , Y. Liu , P. Wang , Z. Zheng , H. Cheng , Y. Dai , B. Huang , Adv. Mater. 2023, 35, 2304133. 10.1002/adma.202304133 37474109

[19] O. Diaz‐Morales , D. Ferrus‐Suspedra , M. T. M. Koper , Chem. Sci. 2016, 7, 2639. 28660036 10.1039/c5sc04486c PMC5477031

[20] D. A. Kolykhalov , A. N. Golysheva , K. S. Erokhin , B. Y. Karlinskii , V. P. Ananikov , ChemSusChem 2025, 18, 202401849. 10.1002/cssc.202401849 39436768

[21] H. Chen , Y. Li , Z. Huang , L. Wang , C. Li , J. He , J. Mater. Chem. A 2025, 13, 8171.

[22] R. Latsuzbaia , R. Bisselink , A. Anastasopol , H. Van der Meer , R. Van Heck , M. S. Yagüe , M. Zijlstra , M. Roelands , M. Crockatt , E. Goetheer , J. Appl. Electrochem. 2018, 48, 611.

[23] C. Lei , Z. Chen , T. Jiang , S. Wang , W. Du , S. Cha , Y. Hao , R. Wang , X. Cao , M. Gong , Angew. Chem., Int. Ed. 2024, 63, 202319642. 10.1002/anie.202319642 38554014

[24] M. L. Krebs , A. Bodach , C. Wang , F. Schüth , Green Chem. 2023, 25, 1797.

[25] Y. Ramli , V. Chaerusani , Z. Yang , Z. Feng , S. Karnjanakom , Q. Zhao , S. Li , Y. Li , A. Abudula , G. Guan , J. Environ. Chem. Eng. 2024, 12, 113666.

[26] L. Xu , Z. Huang , M. Yang , J. Wu , W. Chen , Y. Wu , Y. Pan , Y. Lu , Y. Zou , S. Wang , Angew. Chem., Int. Ed. 2022, 61, 202210123. 10.1002/anie.202210123 36073150

[27] G. Fu , X. Kang , Y. Zhang , Y. Guo , Z. Li , J. Liu , L. Wang , J. Zhang , X. Z. Fu , J. L. Luo , Nat. Commun. 2023, 14, 8395. 38110431 10.1038/s41467-023-43704-2 PMC10728175

[28] J. Woo , B. C. Moon , U. Lee , H.‐S. Oh , K. H. Chae , Y. Jun , B. K. Min , D. K. Lee , ACS Catal. 2022, 12, 4078.

[29] L. Sobota , C. J. Bondue , P. Hosseini , C. Kaiser , M. Spallek , K. Tschulik , ChemElectroChem 2024, 11, 202300151.

[30] S. Li , S. Wang , Y. Wang , J. He , K. Li , Y. Xu , M. Wang , S. Zhao , X. Li , X. Zhong , Adv. Funct. Mater. 2023, 33, 2214488.

[31] Q. Zhou , J. Wang , G. Jin , H. Liu , C. Wang , J. Mater. Chem. A 2024, 12, 12475.

[32] G. Ren , B. Liu , L. Liu , M. Hu , J. Zhu , X. Xu , P. Jing , J. Wu , J. Zhang , Inorg. Chem. 2023, 62, 12534. 37490478 10.1021/acs.inorgchem.3c01774

[33] W. H. Lie , Y. Yang , J. A. Yuwono , C. Tsounis , M. Zubair , J. Wright , L. Thomsen , P. Kumar , N. Bedford , J. Mater. Chem. A 2023, 11, 5527.

[34] R. Ge , Y. Wang , Z. Li , M. Xu , S. M. Xu , H. Zhou , K. Ji , F. Chen , J. Zhou , H. Duan , Angew. Chem., Int. Ed. 2022, 134, 202200211. 10.1002/anie.202200211 35170172

[35] P. Zhou , X. Liu , Z. Chen , C. Tang , X. Zhao , J. Zheng , R. Ge , H. Duan , Adv. Funct. Mater. 2025, 35, 2502081.

[36] D.‐H. Nam , B. J. Taitt , K.‐S. Choi , ACS Catal. 2018, 8, 1197.

[37] D. Chen , Y. Ding , X. Cao , L. Wang , H. Lee , G. Lin , W. Li , G. Ding , L. Sun , Angew. Chem., Int. Ed. 2023, 62, 202309478. 10.1002/anie.202309478 37486710

[38] S. Barwe , J. Weidner , S. Cychy , D. M. Morales , S. Dieckhofer , D. Hiltrop , J. Masa , M. Muhler , W. Schuhmann , Angew. Chem., Int. Ed. 2018, 57, 11460. 10.1002/anie.201806298 29985550

[39] A. Prajapati , N. Govindarajan , W. Sun , J. Huang , H. Bemana , J. T. Feaster , S. A. Akhade , N. Kornienko , C. Hahn , ACS Catal. 2024, 14, 10122. 10.1021/acsami.4c13187 39689259

[40] X. Wen , W. Zhang , S. Yang , Langmuir 2003, 19, 5898.

[41] X. Xia , J. Tu , Y. Zhang , X. Wang , C. Gu , X.‐b. Zhao , H. J. Fan , ACS Nano 2012, 6, 5531. 22545560 10.1021/nn301454q

[42] L. S. Kau , D. J. Spira‐Solomon , J. E. Penner‐Hahn , K. O. Hodgson , E. I. Solomon , J. Am. Chem. Soc. 1987, 109, 6433.

[43] W. Sun , Y. Song , X.‐Q. Gong , L.‐m. Cao , J. Yang , Chem. Sci. 2015, 6, 4993. 30155005 10.1039/c5sc01251a PMC6088437

[44] V. L. Sushkevich , O. V. Safonova , D. Palagin , M. A. Newton , J. A. van Bokhoven , Chem. Sci. 2020, 11, 5299. 34122988 10.1039/d0sc01472a PMC8159279

[45] P. Wu , S. Tan , J. Moon , Z. Yan , V. Fung , N. Li , S. Z. Yang , Y. Cheng , C. W. Abney , Z. Wu , A. Savara , A. M. Momen , D. E. Jiang , D. Su , H. Li , W. Zhu , S. Dai , H. Zhu , Nat. Commun. 2020, 11, 3042. 32546680 10.1038/s41467-020-16674-y PMC7297808

[46] W. F. Xiong , D. H. Si , H. F. Li , X. Song , T. Wang , Y. B. Huang , T. F. Liu , T. Zhang , R. Cao , J. Am. Chem. Soc. 2024, 146, 289. 38135454 10.1021/jacs.3c08867

[47] L. Chen , Z. Yang , Q. Hu , C. Yan , Y. Yao , Y. Bao , Z. Pei , T. Mu , Z. Xue , Angew. Chem., Int. Ed. 2025, 137, 202511868. 10.1002/anie.202511868 40852806

[48] M. C. Biesinger , Surf. Interface Anal. 2017, 49, 1325.

[49] M. C. Biesinger , B. P. Payne , L. W. M. Lau , A. Gerson , R. S. C. Smart , Surf. Interface Anal. 2009, 41, 324.

[50] S. Zhao , C. Tan , C.‐T. He , P. An , F. Xie , S. Jiang , Y. Zhu , K.‐H. Wu , B. Zhang , H. Li , J. Zhang , Y. Chen , S. Liu , J. Dong , Z. Tang , Nat. Energy 2020, 5, 881.

[51] P. Acharya , J. Hong , R. Manso , A. S. Hoffman , L. Kekedy‐Nagy , J. Chen , S. R. Bare , L. F. Greenlee , J. Phys. Chem. C 2023, 127, 11891.

[52] M. Dunwell , Y. Yan , B. Xu , Surf. Sci. 2016, 650, 51.

[53] H. Yun , S. Yoo , J. Son , J. H. Kim , J. Wu , K. Jiang , H. Shin , Y. J. Hwang , Chem 2025, 11, 102461.

[54] C. Lin , J.‐L. Li , X. Li , S. Yang , W. Luo , Y. Zhang , S.‐H. Kim , D.‐H. Kim , S. S. Shinde , Y.‐F. Li , Z.‐P. Liu , Z. Jiang , J.‐H. Lee , Nat. Catal. 2021, 4, 1012.

[55] Z. Yang , L. Chen , Y. Yin , C. Wei , Z. Xue , T. Mu , Energy Environ. Sci. 2024, 17, 8801.

[56] J. Woo , J. Choi , J. Choi , M. Y. Lee , E. Kim , S. Yun , S. Yoo , E. Lee , U. Lee , D. H. Won , Adv. Funct. Mater. 2025, 35, 2413951.

[57] J. F. Xu , W. Ji , Z. X. Shen , W. S. Li , S. H. Tang , X. R. Ye , D. Z. Jia , X. Q. Xin , J. Raman Spectrosc. 1999, 30, 413.

[58] M. W. Louie , A. T. Bell , J. Am. Chem. Soc. 2013, 135, 12329. 23859025 10.1021/ja405351s

[59] P. Hermet , L. Gourrier , J.‐L. Bantignies , D. Ravot , T. Michel , S. Deabate , P. Boulet , F. Henn , Phys. Rev. B 2011, 84, 235211.

[60] P. Zhang , X. Sheng , X. Chen , Z. Fang , J. Jiang , M. Wang , F. Li , L. Fan , Y. Ren , B. Zhang , Angew. Chem., Int. Ed. 2019, 131, 9253. 10.1002/anie.201903936 PMC6617801 31025774

[61] J. Choi , S. Yoo , P. M. Nguyen , E. Lee , H. Shin , Y. J. Hwang , ACS Catal. 2025, 15, 6906.

[62] Z. Yang , S. Wang , C. Wei , L. Chen , Z. Xue , T. Mu , Energy Environ. Sci. 2024, 17, 1603.

[63] C. J. Bondue , M. Spallek , L. Sobota , K. Tschulik , ChemSusChem 2023, 16, 202300685. 10.1002/cssc.202300685 37477393

[64] L. Chico‐Mesa , A. Rodes , R. M. Aran‐Ais , E. Herrero , Nat. Commun. 2025, 16, 3349. 40204756 10.1038/s41467-025-58696-4 PMC11982258

