# Supplementary material for: *OH Adsorption‐Mediated Electrochemical Oxidation of 5‐Hydroxymethylfurfural to Selective 2,5‐Furandicarboxylic Acid at pH 12
Source: Adv Sci (Weinh). 2025 Dec 12;13(10):e18349. doi: 10.1002/advs.202518349 (PMC12915199; doi:10.1002/advs.202518349)
Supplement: Supplementary file 1 — Supporting Information [file ADVS-13-e18349-s001.pdf]

Supporting Information

*OH Adsorption-Mediated Electrochemical Oxidation of 5-Hydroxymethylfurfural to Selective 2,5-Furandicarboxylic Acid at pH 12

Eunchong Lee+ [a], Jinwoo Hwang+ [b], Suhwan Yoo[a], Juhyung Choi[a], Hyun Ji An[a], Sang Heon Han[a], Taerin Kim[a], Jeong Woo Han*[b], Yun Jeong Hwang*[a]

[a]Department of Chemistry, College of Natural Sciences, Seoul National University (SNU), Seoul 08826, Republic of Korea

[b]Department of Materials Science and Engineering, Research Institute of Advanced Materials, Seoul National University (SNU), Seoul 08826, Republic of Korea

[+] These authors contributed equally to this work.

*Correspondence: jwhan98@snu.ac.kr (J. W. H.), yjhwnag1@snu.ac.kr (Y. J. H.)

Experimental Section

Chemicals. 2,5-Furandicarboxaldehyde (DFF, 97%), 2,5-furandicarboxylic acid (FDCA, 97%), 5-(hydroxymethyl)furfural (HMF, 99%), ammonium fluoride (NH_4OH, ≥99.99%), ammonium chloride (NH_4Cl, 99.998%), ammonium persulfate ($NH_4S_2O_8$, ≥98%), boric acid (ReagentPlus®, ≥99.5%), copper(II) oxide (CuO), deuterium oxide (D_2O, 99.9 atom % D), dimethyl sulfoxide (ACS reagent, ≥99.9%), hydrofluoric acid (HF, 48 wt.% in H_2O, ≥99.99%), Nafion™ perfluorinated resin solution (5 wt.% in mixture of lower aliphatic alcohols and water, contains 45% water), nickel(II) sulfate hexahydrate ($NiSO_4 \cdot 6H_2O$, 98%), nickel(II) hydroxide ($Ni(OH)_2$), nickel(II) nitrate hexahydrate ($Ni(NO_3)_2 \cdot 6H_2O$, 98.5%), phosphoric acid (H_3PO_4, ≥85 wt.% in H_2O), potassium carbonate (K_2CO_3, ACS reagent, , ≥99.0%), potassium persulfate ($K_2S_2O_8$, ≥99%), sodium hydroxide (NaOH, 97%), sodium sulfite (Na_2SO_3, ≥98%), sodium tetrachloroaurate(III) dihydrate ($NaAuCl_4 \cdot 2H_2O$, 99%), sodium thiosulfate pentahydrate ($Na_2SO_3 \cdot 5H_2O$, ≥99.5%), sulfuric acid (H_2SO_4, 99.999%), and urea (99.0-100.5%) were purchased from Sigma-Aldrich. 5-(Hydroxy)furan-2-carboxylic acid (HMFCA, >98%), and 5-formyl-2-furancarboxylic acid (FFCA, >98%) were purchased from TCI. Ammonium hydroxide (ammonia water, NH_4OH, 25-30%) was purchased from DAEJUNG. Potassium hydroxide (KOH, 95%) was purchased from SAMCHUN.

Catalyst Synthesis. Cu foam (CF) was pretreated by sonicating 1 M HCl, acetone, and ethanol, respectively, for 10 min. Then, the pretreated CF was dried by N_2 gas and in a vacuum condition for 1 h. Using pretreated CF as a substrate, CuO@NiOOH/CF was synthesized. To be specific, the pretreated CF was immersed in the aqueous solution, a mixture of 1.10 M NaOH and 83 mM $(NH_4)_2S_2O_8$ for 9 min. [1] After as-prepared sample was washed with deionized (DI) water and dried with N_2 gas, it was annealed at 180 °C for 2 h with a ramping speed of 2 °C/min in a

muffle furnace, to form CuO/CF. [2] Then, $Ni(OH)_2$ was chemically deposited on the CuO/CF using the precursor solution of 0.85 M $NiSO_4 \cdot 6H_2O$ and 0.17 M $K_2S_2O_8$. [3] The CuO/CF was immersed in 8.5 mL of the precursor solution for 1 min, and an ammonia solution of 2.5 mL was injected additionally and stayed for 2 min. [4] By washing the water and ethanol, the CuO@$Ni(OH)_2$/CF was dried in an ambient condition.

The surface reconstruction of the CuO@$Ni(OH)_2$/CF was achieved through electrochemical CV activation. LSV was performed from the OCP to 1.9 V vs. RHE 3 times (the detailed condition will be mentioned in the electrochemical measurement section). Then, the CV was conducted from 1.97 V vs. RHE to 0.77 V vs. RHE at a scan rate of 30 mV/s. The CV treatment was repeated 50 times until the redox current density was saturated (**Figure S25**).

A control catalyst, $Ni(OH)_2$ on the nickel foam(NF), was synthesized via hydrothermal reaction. [5] The clean NF was enclosed in a teflon-lined hydrothermal autoclave with the precursor solution, included the 0.375 M $Ni(NO)_3 \cdot 6H_2O$, 0.144 M urea, and 0.042 M NH_4F. After 6 h at 120 °C, as-prepared $Ni(OH)_2$/NF was washed with DI water and ethanol, and dried in the air at 60 °C for 3 h and in vacuum for 3 h, respectively.

Materials Characterization. Field emission scanning electron microscopy (FE-SEM) were performed to investigate the morphology of the catalyst using Apreo 2 S Hivac (Thermo Fisher Scientific). Crystal information was obtained via X-ray diffraction (XRD) patterns through D8 advance (Bruker) diffractometer with Cu K_α radiation. Spherical aberration-corrected scanning transmission electron microscopy (Cs-STEM) using JEM-ARM200F (JEOL) introduced high-resolution images with lattice fringe analysis and energy dispersive spectroscopy (EDS) mapping of the catalysts. X-ray photoelectron spectroscopy (XPS) was performed to analyze the electronic state of the surface by K-Alpha + XPS system (Thermo Fisher Scientific) with a

monocrhromated Al K_α X-ray source. X-ray adsorption spectroscopy (XAS) was conducted at 8C (Nano XAFS) beamline at a storage ring of 3.0 GeV by Si (111) double crystal monochromator in Pohang accelerator laboratory. The spectra were collected based on a transmittance and fluorescence mode. The analysis of acquired data was performed using Athena and Arthemis software.

Electrochemical Measurements. Electrochemical experiments were performed using a VSP-3e potentiostat (Biologic). Two compartmented H-cells separated by a proton exchange membrane (PEM, Naftion 211) were utilized in electrochemical measurements except the CV activation process (one-pot cell). The synthesized catalysts (CuO, CuO@$Ni(OH)_2$, $Ni(OH)_2$) of 2 × 1 cm^2 (a geometrical area was set as 4 cm^2 considered by both the front and back sides) were applied as the working electrode. Both a working electrode and a Hg/HgO (1 M KOH) reference electrode were located in an anolyte section. A Pt mesh in a cathode compartment was used as a counter electrode. A phosphate buffer (PB) solution was prepared by adding KOH about 2.0 -2.5 equivalent amounts to 0.5 M (0.25 M and 0.75 M) phosphoric acid solution according to the phosphate titration curve. [6] A precise KOH addition was adjusted by pH meter measurement until it reached pH 12, which was used for the electrolyte. [7] Similarly, 0.75 M borate buffer solution was prepared following the same protocol used for PB. 15 mL of pH 12 electrolyte was filled in each compartment of the H-cell, respectively. The proper amounts of the HMF was added to the anolyte when its concentration was noted. If not noted in the results, iR compensation was not conducted to prevent a distorted current peak. The applied potential versus the reversible hydrogen electrode (RHE) was converted, following equation below.

$$E(\text{vs. RHE}) = E(\text{vs. Hg/HgO}) + 0.098\ V + 0.05916\ V \times pH$$

For the HMFOR using a membrane electrode assembly (MEA) electrolyzer, 50 mM HMF was

included in the 0.5 M PB (pH 12) electrolyte, and the synthesized CuO@NiOOH/CF catalyst was used as the working electrode in the anode side with a geometrical area of 5 cm^2. The Pt mesh was utilized as the counter electrode, and Nafion 117 PEM was assembled between the anode and cathode parts. Both catholyte and anolyte were circulated during HMFOR by a peristaltic pump with a flow rate of 20 mL min^{-1}.

Product Analysis. A quantifications of reactant, intermediate, and product molecules involved in HMFOR were analyzed by high-performance liquid chromatography (HPLC, SPD-M40 (Shimadzu)). [8] A mixture separation was performed via Aminex HPX-87H column (BIO-RAD) at 35 °C. 5 mM H_2SO_4 as a mobile phase was used with 0.5 mL min^{-1} flow rate. An eluent was prepared by diluting the obtained electrolyte for 60 μL with 900 μL of 5 mM H_2SO_4. A detection of the organic molecules was performed using a photodiode array (PDA) detector at optimized wavelength showing maximized intensities at 284, 264, 283, 259, and 289 nm for HMF, FDCA, FFCA, HMFCA, and DFF, respectively. To quantify the concentration of organic molecules, The calibration curves were obtained based on the variation of the concentration of different molecules (HMF, FDCA, FFCA, HMFCA, DFF), respectively (**Figure S8**). HMF conversion, yield of products, Faradaic efficiency (FE), and carbon balance values were calculated based on the following equations.

$$\text{HMF conversion (\%)} = \frac{\text{mol of HMF at charge passed}}{\text{mole of initial HMF}} \times 100\ (\%)$$

$$\text{Yield of products (\%)} = \frac{\text{mol of product at charge passed}}{\text{mole of initial HMF}} \times 100\ (\%)$$

$$\text{Faradaic efficiency (FE) (\%)} = \frac{\text{mol of product at charge passed} \times F \times n}{\text{total charge passed}} \times 100\ (\%)$$

$$\text{Carbon balance (\%)} = 100 - (\text{HMF conversion} - \textstyle\sum \text{yield of products})$$

Where F is the Faradaic constant, 96,485 C mol^{-1}. n is the number of electrons released during the HMF oxidation following equations.

$$C_6H_6O_3 \text{ (HMF)} + 2\ OH^- \rightarrow C_6H_6O_4 \text{ (HMFCA)} + 2\ e^- + H_2O$$

$$C_6H_6O_3 \text{ (HMF)} + 2\ OH^- \rightarrow C_6H_4O_3 \text{ (DFF)} + 2\ e^- + 2\ H_2O$$

$$C_6H_6O_3 \text{ (HMF)} + 4\ OH^- \rightarrow C_6H_4O_4 \text{ (FFCA)} + 4\ e^- + 3\ H_2O$$

$$C_6H_6O_3 \text{ (HMF)} + 6\ OH^- \rightarrow C_6H_4O_5 \text{ (FDCA)} + 6\ e^- + 4\ H_2O$$

^{1}H nuclear magnetic resonance (NMR) spectroscopy was utilized to investigate the HMF degradation effect at different pH conditions. 450 μL of 50 mM HMF liquid sample in 1 M KOH (or 0.5 M PB) was mixed with 50 μL of D_2O containing 50 mM DMSO as an internal standard. The NMR spectra were obtained using a prepared NMR sample on a Varian 500 MHz spectrometer. To obtain the product powder, the pH of the anolyte was decreased to near 3 by adding 3 M HCl after long-term HMFOR, followed by centrifugation.

***Operando* Raman Spectroscopy Measurement.** Raman spectroscopy was conducted using an InVia Qontor (Renishaw) Raman microscope. [8] A 532 nm laser was utilized with 5% neutral density (ND) filter. A signal acquisition was performed for 20 s with accumulation twice. *Operando* Raman spectroscopy was obtained using a custom-made electrochemical cell with a 63x water-immersion objective lens (Leica). The custom-made electrochemical was comprised of the working electrode as the synthesized catalyst, the counter electrode as the Pt wire, and the reference electrode of Hg/HgO (1 M KOH) based on the three-electrode system. The 0.5 M PB (pH 12) was used for the electrolyte, and HMF was added when it is noted. The multi-step chronoamperometry was performed from low to high applied potential to observe the phase transition from $Ni(OH)_2$ to NiOOH behavior using the potentiostat of Vertex. 5A (IVium).

A time-resolved Raman spectroscopy (TRS) was conducted based on the same component of *Operando* Raman spectra measurement except for the acquisition time of 3 s with the accumulation of 3. After applying the potential at 1.45 V vs. RHE for 30 min with 50 mM HMF condition, Raman spectra were captured at intervals of 10 s.

***Operando* XAS Measurement**. An *Operando* XAS was performed in the 8C (Nano XAFS) beamline same as *ex-situ* characterization. A custom-made cell designed for *in situ* XAS was used to investigate the electronic state and structural information under applied potential. [9] The working electrode was prepared by the following procedure. The synthesized catalysts were collected, and loaded on carbon paper (CP, AvCarb) via spray coating. The ink was prepared by mixing 1 mg catalyst, 1 mL of ethanol (or a mixture of 800 μL ethanol and 200 μL water), and 20 μL of Nafion solution. The prepared catalyst on CP as a working electrode was attached to the Kapton tape window in the electrochemical cell to detect the X-ray absorption signal. The Pt mesh and Hg/HgO were used as counter, and reference electrodes, respectively. The spectra were obtained based on the fluorescence mode using a passivated implanted planar silicon (PIPS) detector and Si drift detector (SDD) which was utilized to observe a small amount of metal element for Ni K-edge in $CuO@Ni(OH)_2$.

***Operando* ATR-SEIRAS** A thin Au film was deposited on the Si crystal (20 mm in diameter, VeeMAX) with the previously reported method to obtained surface enhanced infrared absorption spectroscopy. [10] Catalysts were loaded with a spray-coating method on an Au-deposited Si crystal. Catalyst ink was prepared by sonicating for 30 min with a composition of 1 mg of the catalyst, 20 μL of Nafion solution, and 1 mL of ethanol. Catalyst/Au-Si (WE) was assembled into a custom-made cell, and it was transferred to the Fourier transform infrared

spectroscopy (FT-IR, VERTEX 80v, Bruker), equipped with a mercury cadmium telluride (MCT) detector and an attenuated total reflection (ATR) accessory (VeeMAX III, PIKE Technologies). A Pt wire and a Hg/HgO reference (saturated 1 M KOH) were utilized for the counter and reference electrode, respectively. All spectroscopic measurements were conducted with a 4 cm^{-1} spectral resolution and 16 scans. The reference spectrum was obtained at OCP, and all spectra were expressed in absorbance, $-\log(R/R_0)$.

Computational details Spin-polarized DFT calculations were performed using Vienna Ab initio Simulation Package (VASP, version 6.3.2). [11-12] Generalized gradient approximation with Perdew-Burke-Ernzerhof functional (GGA-PBE) was used as the exchange correlation functional. [13] For correction of weak van der Waals interaction, the DFT-D3 method with Becke-Jonson damping was adopted. [14-15] The DFT+U method based on Dudarev's scheme was employed with U_{eff} (U-J correction) value of 8.0 eV [16] and 5.5 eV [17] for Cu and Ni, respectively. The Brillouin zone was sampled with a 3 × 3 ×1 k-point mesh [18] for slab models with 400 eV cut-off energy. To prevent interactions between the periodic images in z-direction, a 15 Å vacuum layer was introduced. The CuO (111) slab model has four layers, where the bottom two layers were fixed, while the NiOOH (001) slab model has a single layer with full relaxation. [17] The relaxation thresholds for all structures, including the bulk structures, were set to 0.03 eV/Å for force convergence and 10^{-5} eV energy convergence.

The adsorption energy (E_{ads}) was obtained by following equation of (1) :

$$E_{ads} = E_{adsorbate/surface} - E_{surface} - E_{adsorbate} \quad (1)$$

, where $E_{adsorbate/surface}$ is the total energy of surface models with adsorbates on its surface, $E_{surface}$ is the total energy of bare slab models, and $E_{adsorbate}$ is the gas-phase energy of adsorbates.

The d-band center of metal site was obtained by following equation of (2) :

$$\frac{\int_{-\infty}^{E_f} E\rho_d(E)\, dE}{\int_{-\infty}^{E_f} \rho_d(E) dE} \tag{2}$$

, where E_f is the energy of Fermi level and ρ_d is the projected density of states of metal site of d-band.

As shown in supplementary **Figure S3a-b**, the slab model of CuO (111) was constructed with a unit cell of 11.5408 Å in the x-direction and 12.0024 Å in the y-direction, while NiOOH (001) was modelled with a unit cell size of 11.7746 Å in both x- and y- direction. To obtain a CuO@NiOOH model with a maximum Cu/Ni interface, the NiOOH (001) surface was adsorbed onto the CuO (111) surface at the site where the adsorption of NiOOH exhibits the lowest energy. Moreover, a half-sized NiOOH (001) surface (x = 5.8873 Å, y = 11.7746 Å) was adsorbed onto the CuO (111) surface along either the x- or y-direction of the CuO (111) surface to expose both CuO (111) and NiOOH (001) sites in the CuO@NiOOH model. The lattice mismatch between NiOOH y-direction and the CuO x-direction of the substrate is 1.99 %, resulting in compressive strain on NiOOH, while the mismatch between the NiOOH y-direction and the CuO y-direction is 1.93%, leading to tensile strain on NiOOH. Among the optimized CuO@NiOOH structures, the adsorption of NiOOH along the CuO y-direction is 2.55 eV more stable than along the CuO x-direction, indicating that NiOOH under tensile strain is more stable than under compressive strain.

The Gibbs free energy (ΔG) of HMF oxidation on CuO (111), NiOOH(001), and CuO@NiOOH was calculated relative to gaseous HMF, H_2O, and H_2. For example, the Gibbs free energy of $\Delta G_{HMF\rightarrow HMFCA}$ is calculated as equation of (3):

$$\Delta G_{HMF\rightarrow HMFCA} = \Delta G_{rxn} - \Delta G_{gas} \tag{3}$$

$$\Delta G_{rxn} = (E + ZPE – TS)_{HMFCA*} - (E + ZPE – TS)_{HMF*}$$

$$\Delta G_{gas} = (E + ZPE - TS)HMF_{(g)} - (E +ZPE -TS)H_{2(g)} + (E +ZPE -TS)H_2O_{(g)}$$

, where E is the total energy, ZPE is the zero-point energy, T and S are the environment temperature and entropy, respectively. The ZPE was determined from the vibrational frequencies with a harmonic quantum oscillator, which were obtained through second-order finite difference numerical differentiation of forces using a step-size of 0.015 Å. To account for the electrochemical environment, computational hydrogen electrode (CHE) [19] was employed with T = 298 K and U_{RHE} = 0.0 V. By using the computational hydrogen electrode method, calculation of the Gibbs free energy change of the elementary step includes the half-cell potential of the reaction $OH^- + 1/2H_2 \rightarrow H_2O + e^-$. Hence, the change of Gibbs free energy during the proton-electron transfer step can be further corrected using equation (4):

$$\Delta G = \Delta E - \Delta ZPE - T\Delta S + eU_{RHE} \qquad (4)$$

, where ΔE is the total energy difference, ΔZPE is the difference in zero-point energy, T is the environment temperature, ΔS is the change of entropy, and eU_{RHE} is the elementary charge multiplied by applied potential.

Supporting Information

NMR Spectroscopy of HMF depending on the different pH conditions.

To investigate these OH^--mediated reactions, we observed the NMR spectra of 50 mM HMF depending on the different pH conditions with DMSO as an internal standard (**Figure S1**). NMR peaks at 9.22, 7.31, 6.44, and 4.47 ppm were confirmed to be protons derived from the HMF (a, b, c, d), respectively (red line) at pH 12 (0.5 M PB). In the case of 1 M KOH, the overall NMR peaks of HMF were observed at a lower chemical shift, because HMF interacts with a more negative charge from the abundant electrons of OH-, resulting in an enhanced shielding effect (blue line). Furthermore, the peak, the proton of the aldehyde position of HMF, showed a more increased shielding effect with broader peak shapes compared to the other proton peaks. These results indicate that the aldehyde of HMF interacts more with OH^-, forming geminal-diolate (or diol), and fast kinetics between aldehyde and geminal-diolate mediated by OH^- induce the broader peak shapes. [20]

To investigate the HMF degradation behavior further, we also observed the NMR spectra over time (**Figure S2**). At pH 12, HMF remains at a similar peak position and intensity over time (**Figure S2a**). However, the overall intensity of HMF peaks decreased in 1 M KOH with the appearance of new peaks (**Figure S2b**). The aldehyde peaks became broader due to interaction of OH^-, and the various peaks derived from the OH^--mediated side reaction were observed near 7.0-6.0 ppm. Interestingly, the peaks featured at 6.06 ppm, assigned to the geminal diol, increased because the OH^--mediated geminal diol formation is promoted under strongly alkaline conditions. [21] These species further react with other furan ring compounds, accelerating the disproportionation by Canizzaro reaction or humin polymerization, which is well matched with our results as shown in **Figure 1b**. Furthermore, the peak at 8.23 ppm, associated with formic acid, increased over time as by-products derived from HMF degradation

via the ring-opening mechanism. Therefore, the HMF degradation by OH^- can be successfully suppressed at pH 12.

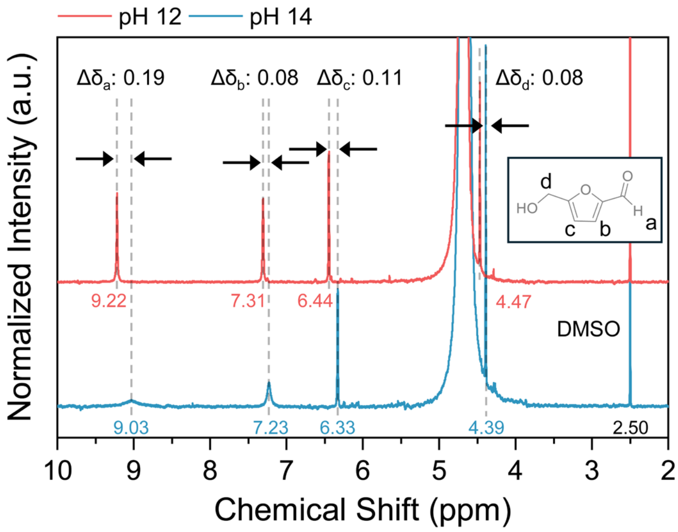

Figure S1 NMR spectra of 50 mM HMF with DMSO as an internal standard at pH 12 (0.5 M PB, red line) and pH 14 (1 M KOH, blue line).

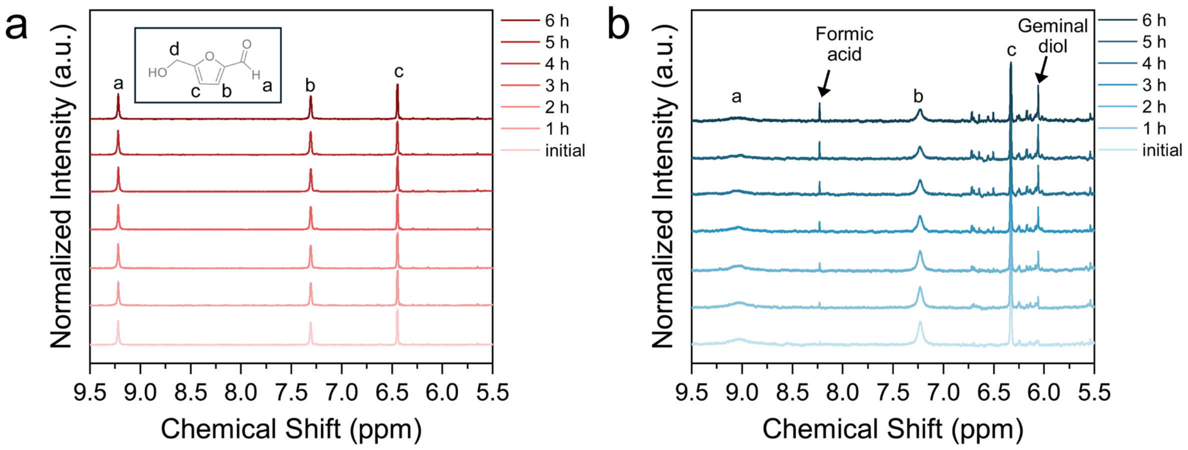

Figure S2 NMR spectra of 50 mM HMF depending on the passed time at (a) pH 12 and (b) pH 14.

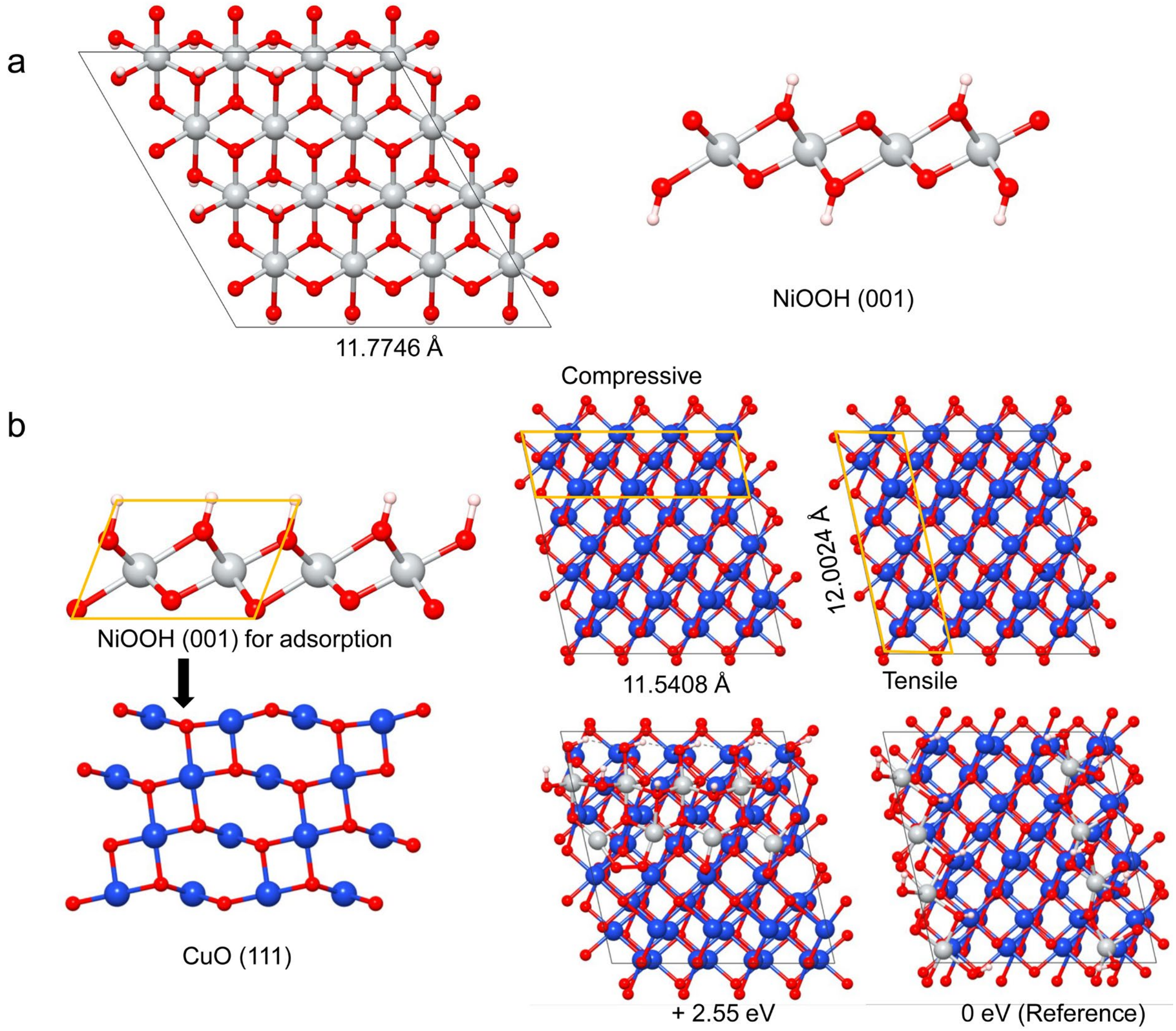

Figure S3 (a) Slab model of NiOOH (001) with location of hydrogen atoms with chain-saw-like placement. (b) Comparison of NiOOH (001) adsorption over CuO (111) under tensile and compressive strain. The modeling scheme illustrates the adsorption configurations, and the relative energy values indicate the thermodynamic stability of each case, highlighting which case is more thermodynamically stable.

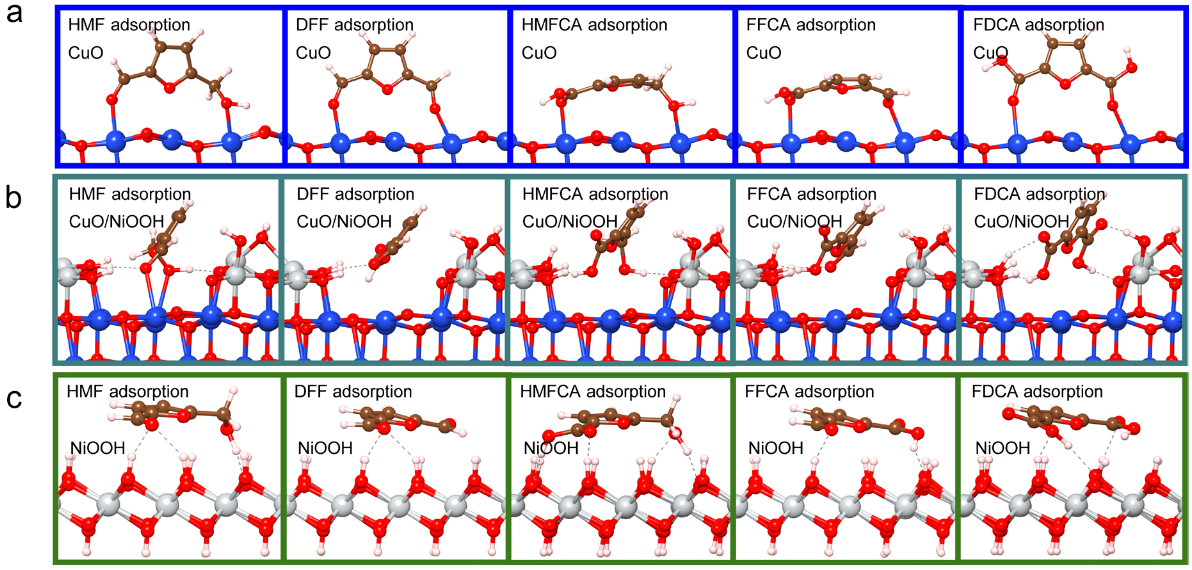

Figure S4 (a) The most stable adsorption configuration of HMF, DFF, HMFCA, FFCA and FDCA on Cu (111). (b) The most stable adsorption configuration of HMF, DFF, HMFCA, FFCA and FDCA on CuO/NiOOH. (c) The most stable adsorption configuration of HMF, DFF, HMFCA, FFCA and FDCA on NiOOH (001).

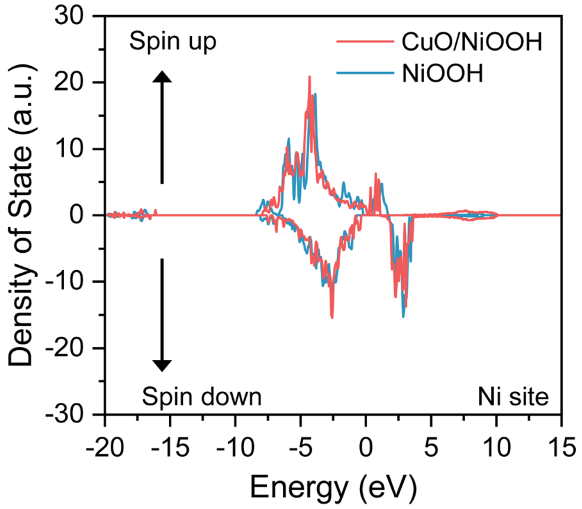

Figure S5 PDOS calculations for NiOOH and Ni sites in CuO/NiOOH.

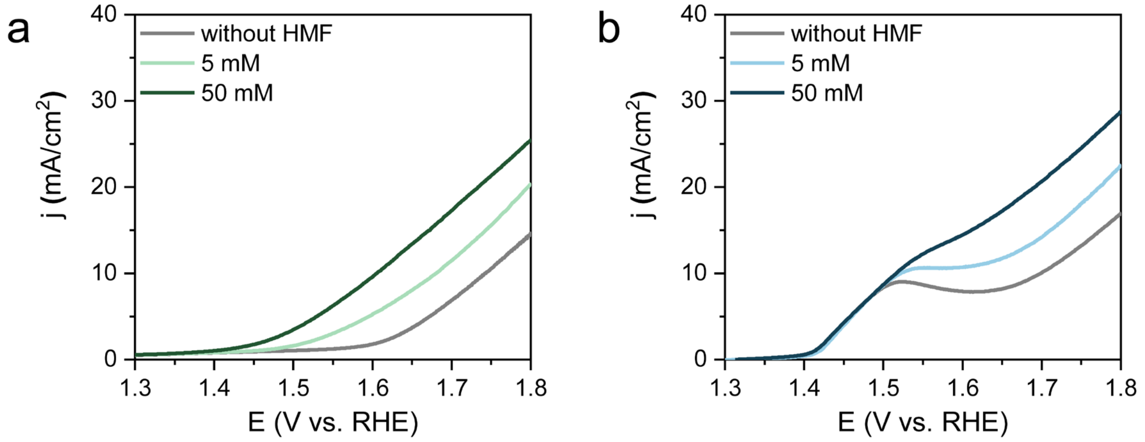

Figure S6 LSV curves of (a) CuO and (b) $Ni(OH)_2$ depending on the concentration of HMF (without HMF, 5 mM, 50 mM HMF) at pH 12 electrolyte (0.5 M PB) at a scan rate of 5 mV sec^{-1}.

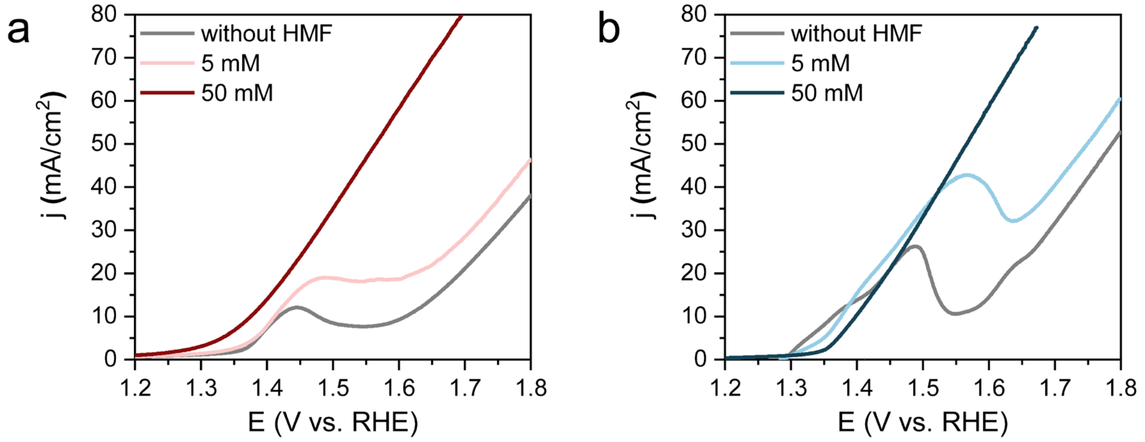

Figure S7 LSV curves of (a) CuO@NiOOH and (b) $Ni(OH)_2$ depending on the concentration of HMF (without HMF, 5 mM, 50 mM HMF) in 1 M KOH at a scan rate of 5 mV sec^{-1}.

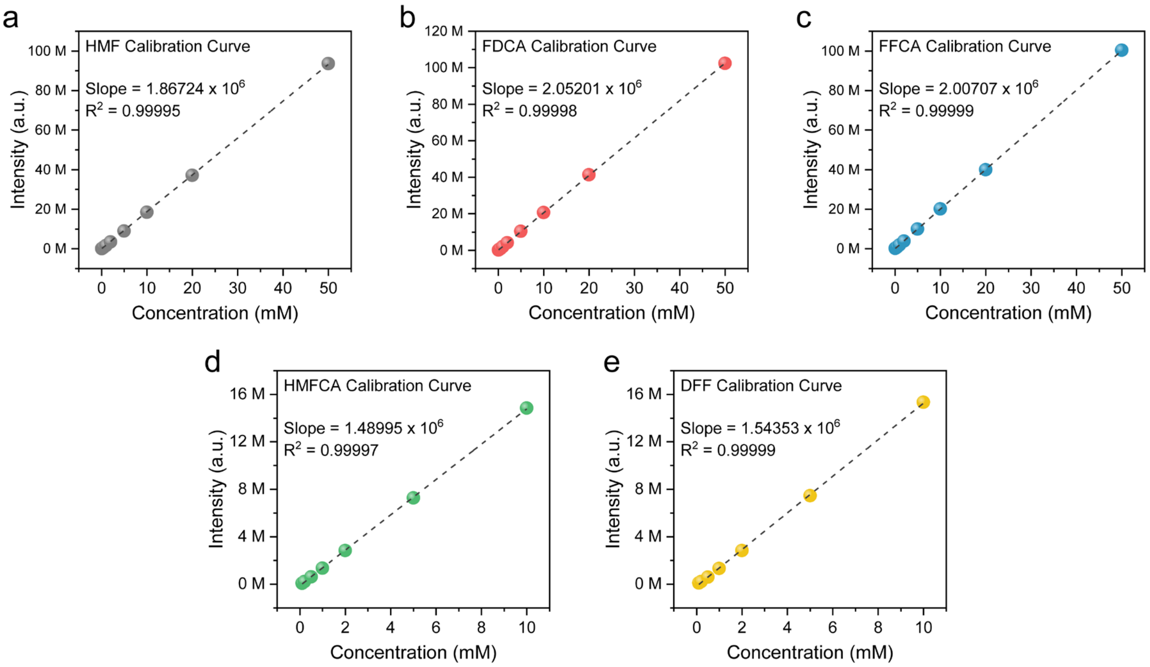

Figure S8 HPLC calibration curves depending on the concentration of (a) HMF, (b) FDCA, (c) FFCA, (d) HMFCA, and (e) DFF. The suffix M is 10^6.

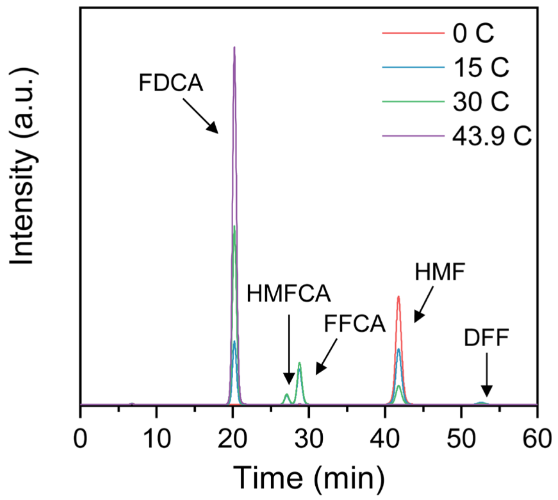

Figure S9 Chromatogram of HPLC for HMF and its products depending on the passed charge during HMFOR using CuO@NiOOH with 5 mM HMF at pH 12 at 1.45 V vs. RHE. The retention times of FDCA, HMFCA, FFCA, HMF, and DFF were 20.2 min, 27.1 min, 28.8 min, 41.8 min, and 52.6 min, respectively.

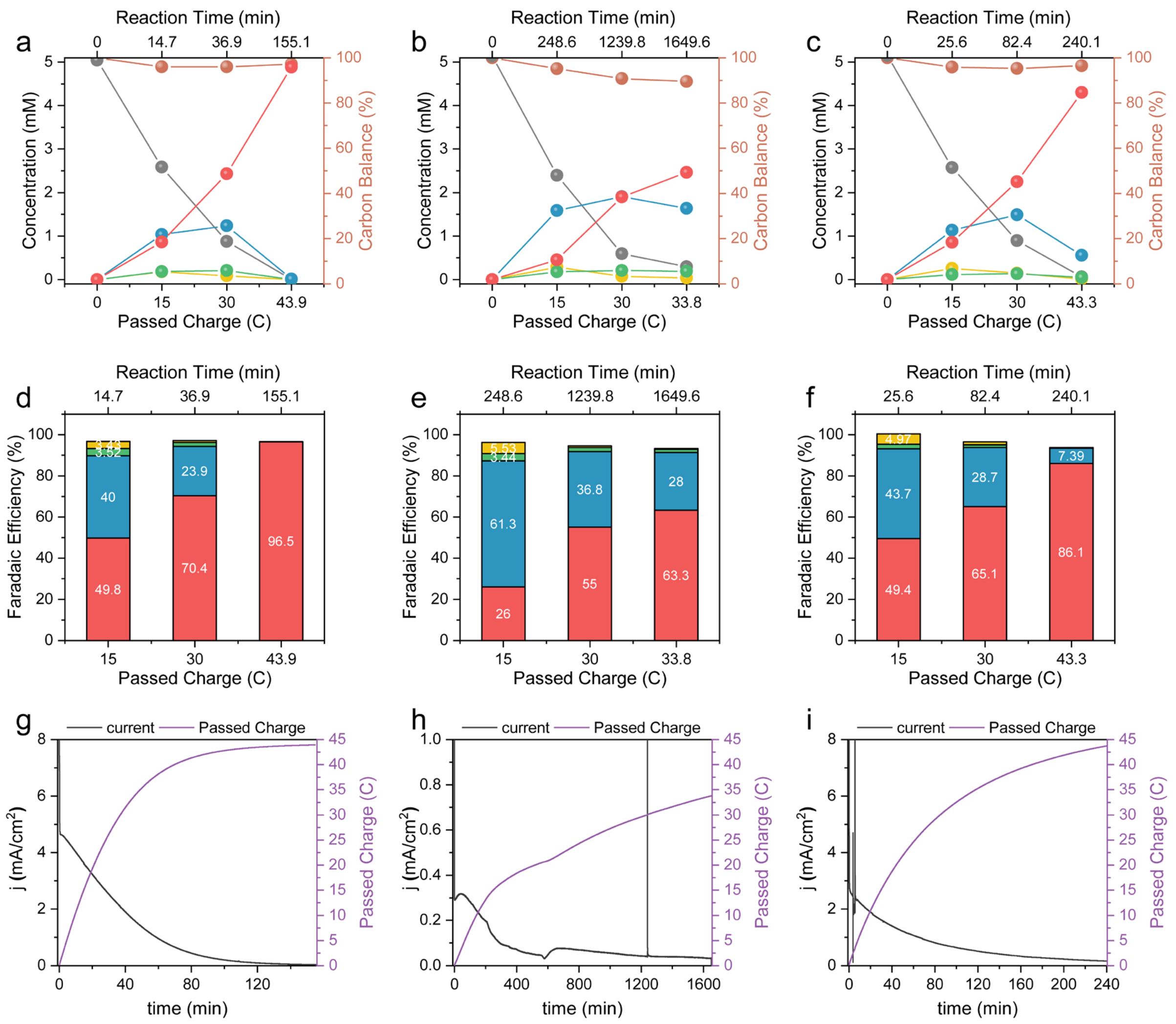

Figure S10 HMFOR electrolysis under 5 mM HMF at pH 12 via CA at 1.45 V vs. RHE. Concentration changes of HMF and its products versus passed charges for (a) CuO@NiOOH, (b) CuO, and (c) $Ni(OH)_2$. Faradaic efficiency versus passed charges for (d) CuO@NiOOH, (e) CuO, and (f) $Ni(OH)_2$. CA curves recorded during HMFOR for (g) CuO@NiOOH, (h) CuO, and (i) $Ni(OH)_2$. Each organic compound is indicated by a specific color: HMF (gray), FDCA (red), FFCA (blue), HMFCA (green), and DFF (yellow).

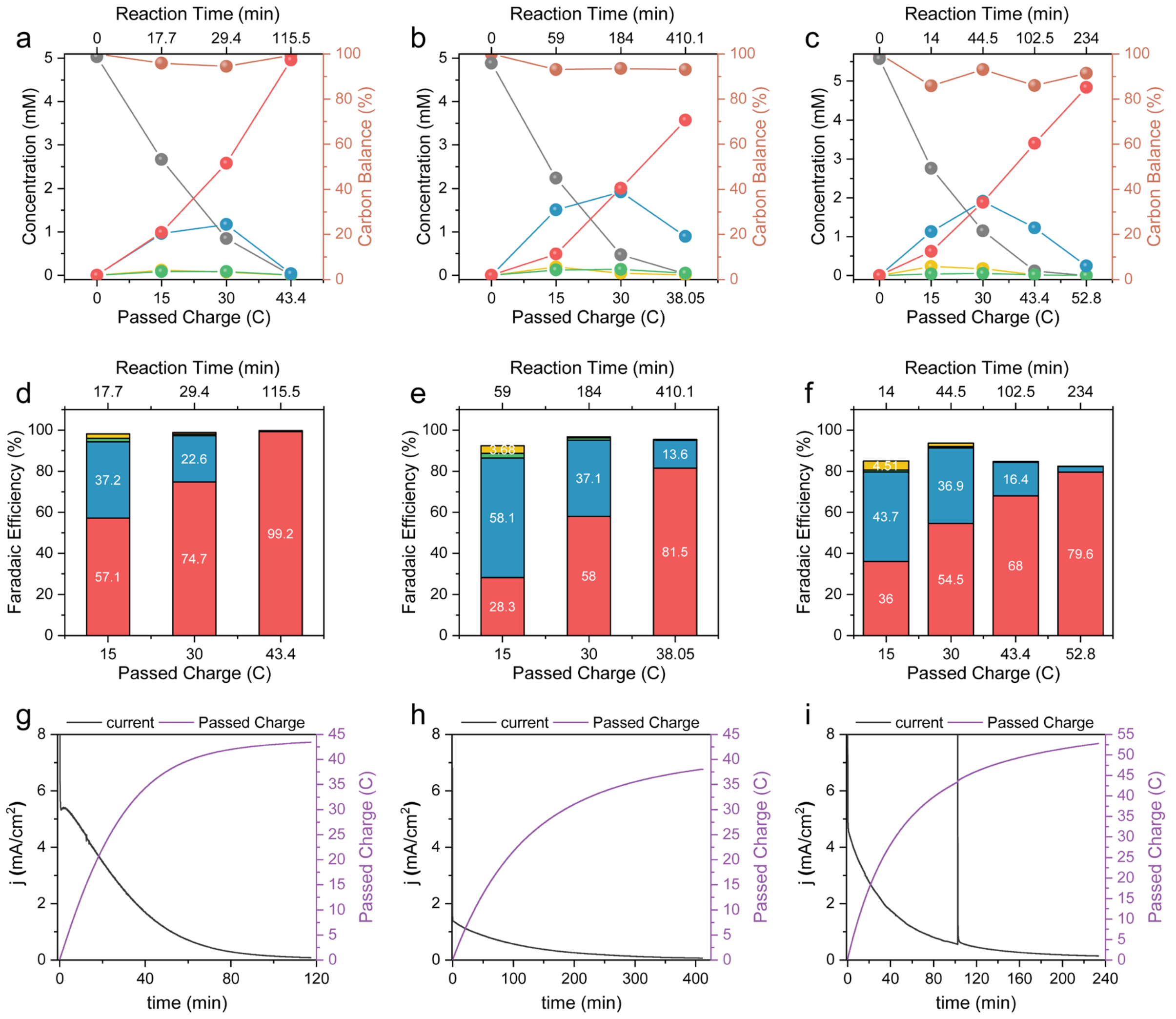

Figure S11 HMFOR electrolysis under 5 mM HMF at pH 12 via CA at 1.50 V vs. RHE. Concentration changes of HMF and its products versus passed charges for (a) CuO@NiOOH, (b) CuO, and (c) $Ni(OH)_2$. Faradaic efficiency versus passed charges for (d) CuO@NiOOH, (e) CuO, and (f) $Ni(OH)_2$. CA curves recorded during HMFOR for (g) CuO@NiOOH, (h) CuO, and (i) $Ni(OH)_2$. Each organic compound is indicated by a specific color: HMF (gray), FDCA (red), FFCA (blue), HMFCA (green), and DFF (yellow).

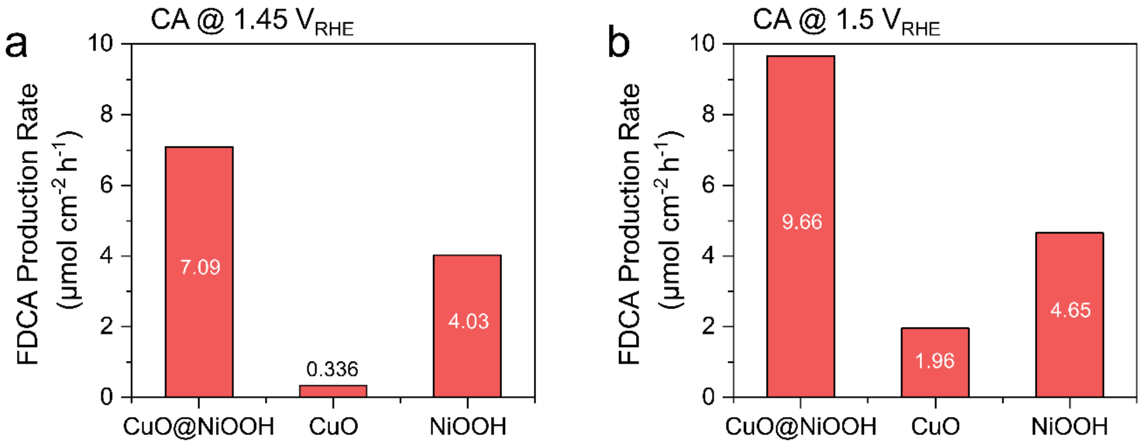

Figure S12 FDCA production rate depending on the catalysts of CuO@NiOOH, CuO, and $Ni(OH)_2$ at (a) 1.45 V vs. RHE and (b) 1.5 V vs. RHE under 5 mM HMF at pH 12 (0.5 M PB).

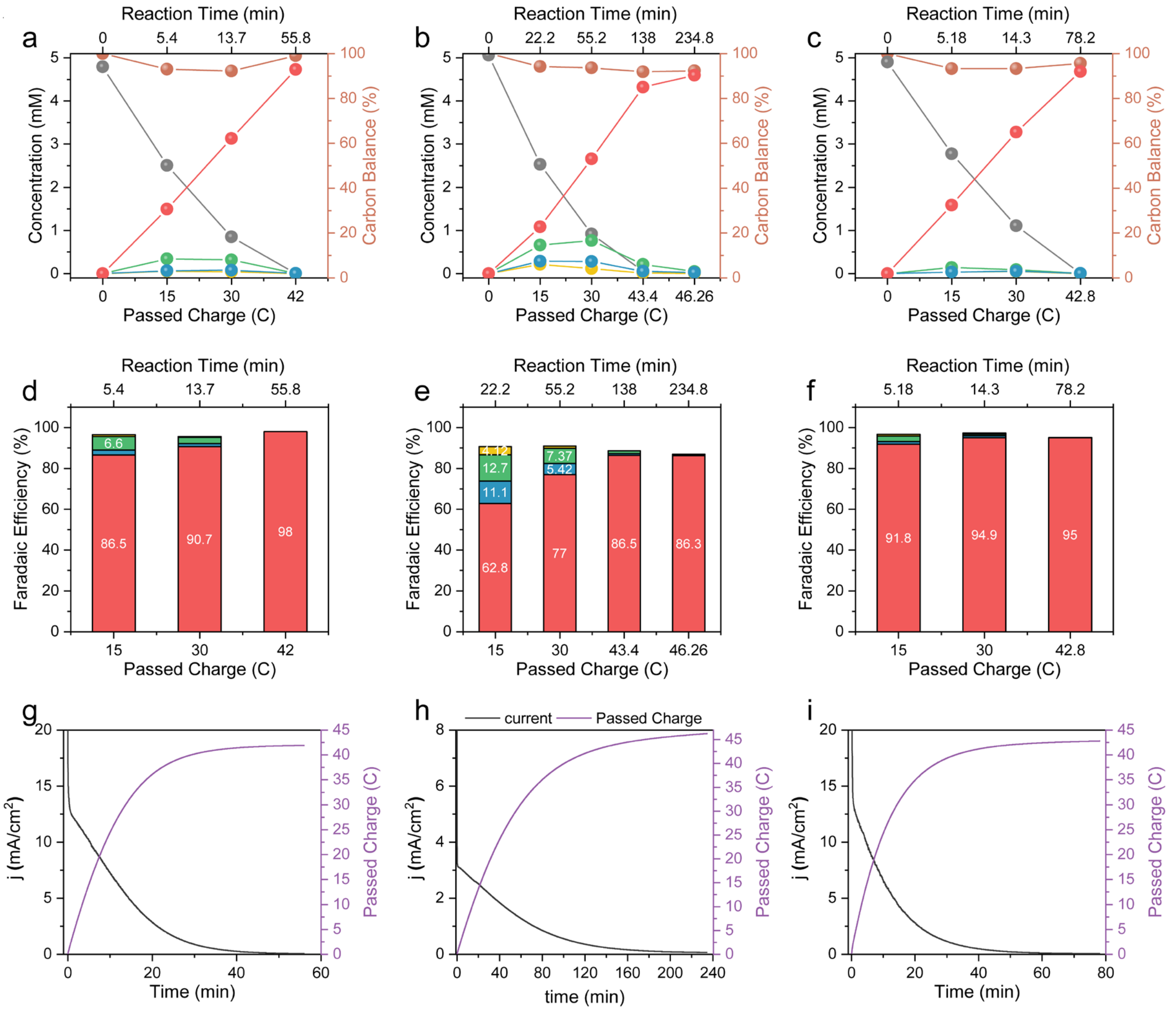

Figure S13 HMFOR electrolysis under 5 mM HMF in 1 M KOH via CA at 1.39 V vs. RHE. Concentration changes of HMF and its products versus passed charges for (a) CuO@NiOOH, (b) CuO, and (c) $Ni(OH)_2$. Faradaic efficiency versus passed charges for (d) CuO@NiOOH, (e) CuO, and (f) $Ni(OH)_2$. CA curves recorded during HMFOR for (g) CuO@NiOOH, (h) CuO, and (i) $Ni(OH)_2$. Each organic compound is indicated by a specific color: HMF (gray), FDCA (red), FFCA (blue), HMFCA (green), and DFF (yellow).

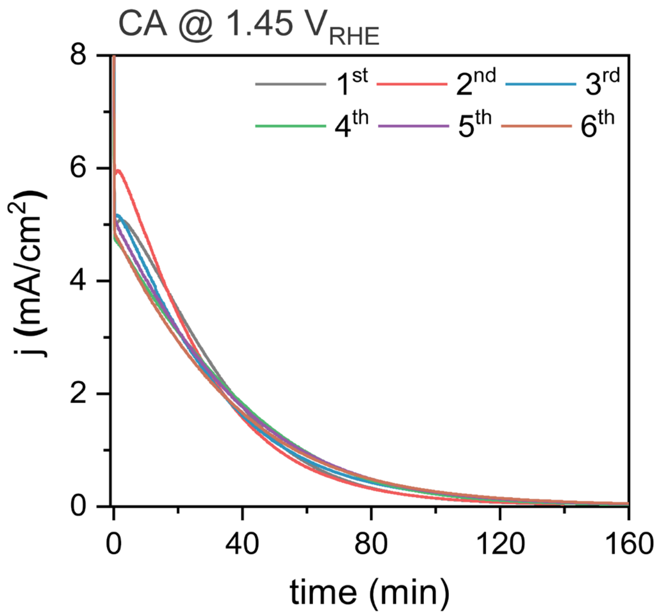

Figure S14 CA curves for successive cycles of HMFOR using CuO@NiOOH with 5 mM HMF at pH 12 at 1.45 V vs. RHE.

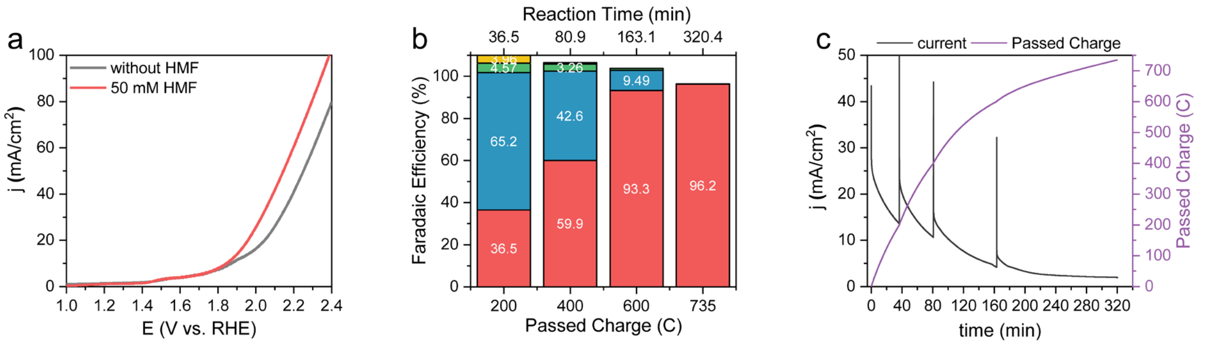

Figure S15 (a) LSV curves of CuO@NiOOH catalyst using MEA electrolyzer at pH 12 without/with 50 mM HMF at a scan rate of 10 mV sec^{-1}. (b) Plot of Faradaic efficiency versus passed charge. (c) CA curve during HMFOR with 50 mM HMF using CuO@NiOOH. During HMFOR electrolysis, injection of KOH was performed to maintain a pH 12. Each organic compound is indicated by a specific color: HMF (gray), FDCA (red), FFCA (blue), HMFCA (green), and DFF (yellow).

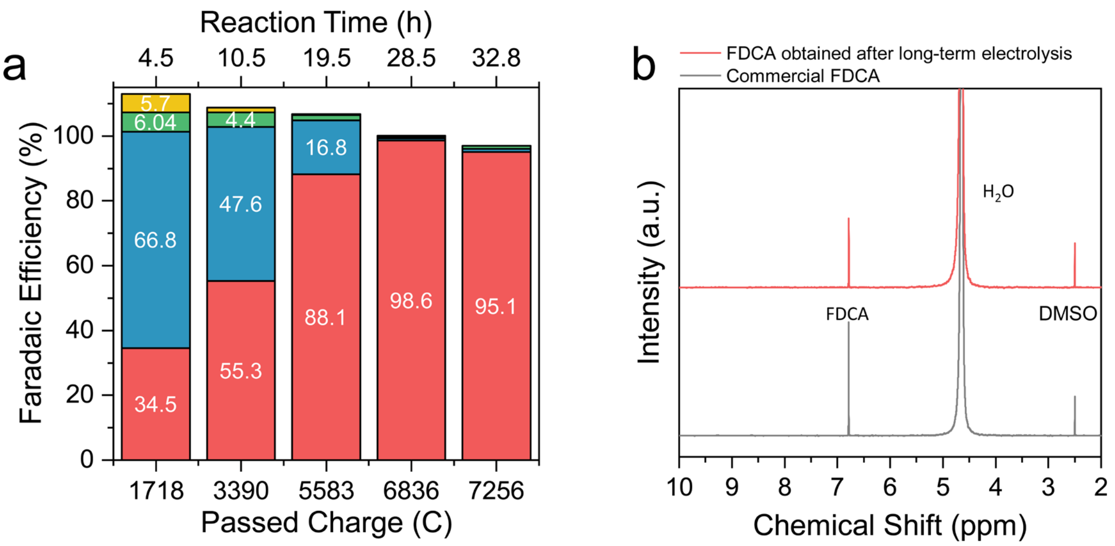

Figure S16 (a) Plot of Faradaic efficiency versus passed charge during HMFOR elctrolysis under 50 mM HMF in 0.5 M PB of 250 mL via CA at 2.0 V using CuO@NiOOH. (b) NMR spectra of commercial FDCA and FDCA obtained after long-term electrolysis. Each organic compound is indicated by a specific color: HMF (gray), FDCA (red), FFCA (blue), HMFCA (green), and DFF (yellow).

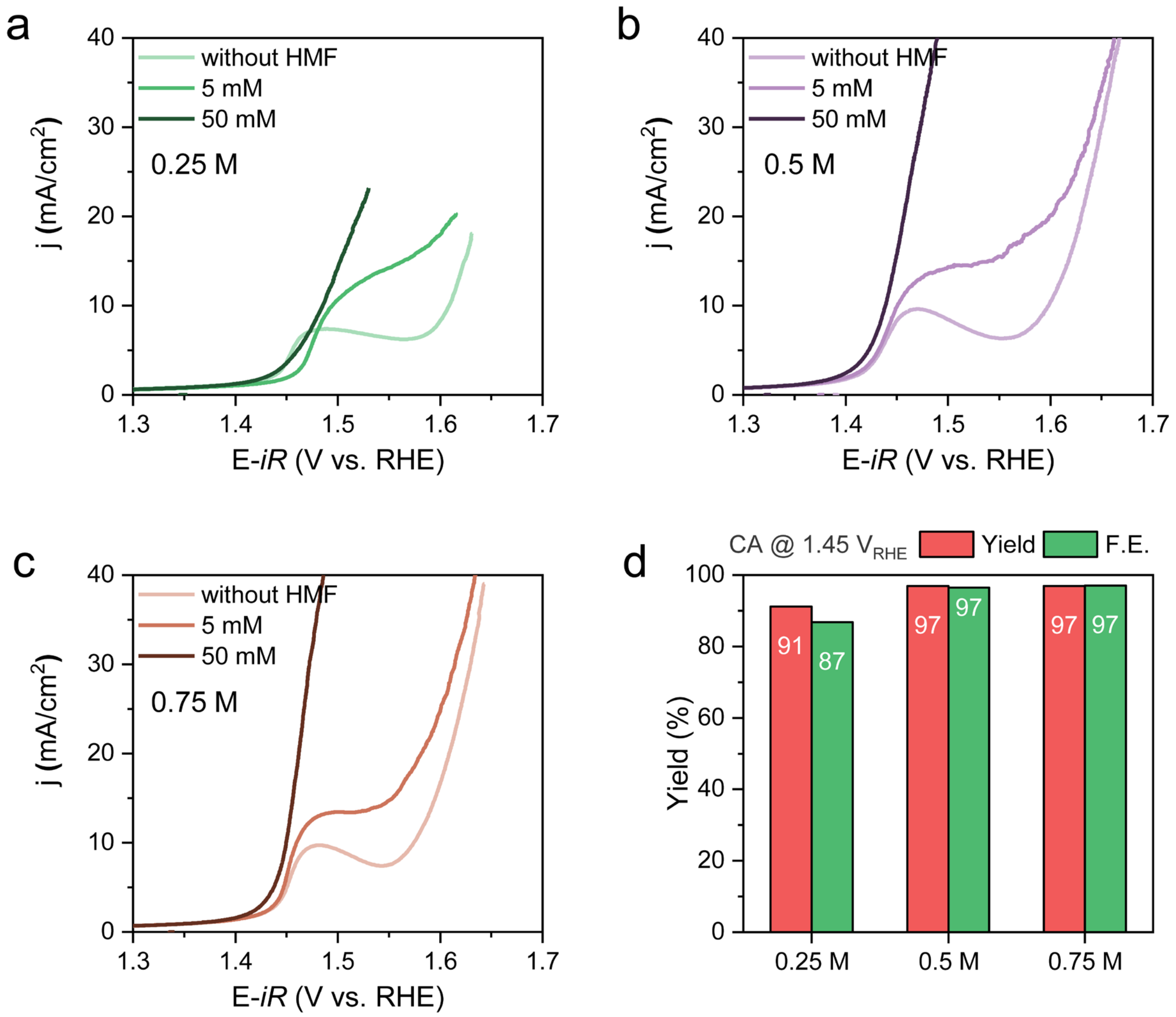

Figure S17 Phosphate buffer (PB) concentration dependence. LSV curves of CuO@NiOOH for HMFOR in (a) 0.25 M, (b) 0.5 M, (c) 0.75 M PB at a scan rate of 5 mV sec^{-1}. Due to R_s difference related to ionic strength, the iR compensation was conducted. (d) FDCA yield and FE depending on the PB concentration using CuO@NiOOH with 5 mM HMF at 1.45 V vs. RHE.

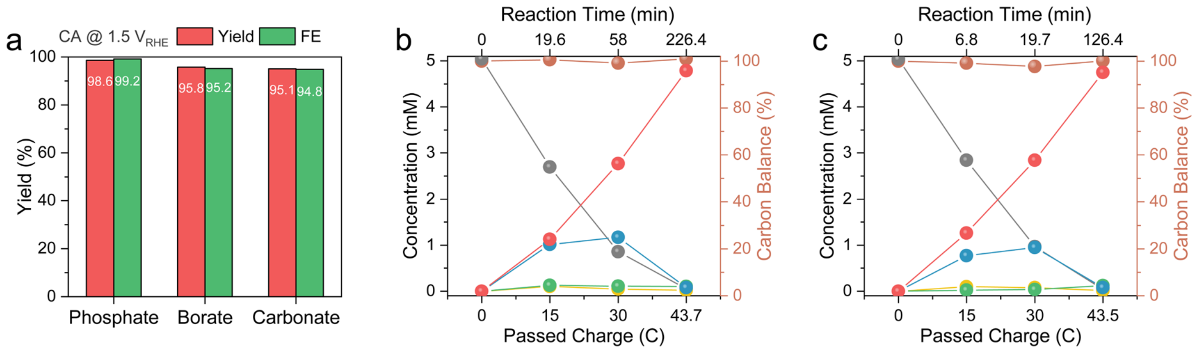

Figure S18 (a) FDCA yield and FE results depending on the electrolyte near pH 12. Phosphate is the 0.5 M phosphate buffer, borate is the 0.75 M borate buffer, and carbonate is the 1 M K_2CO_3. Concentration changes of HMF and its products versus passed charge during HMFOR with 5 mM HMF at 1.5 V vs. RHE in (b) 0.75 M borate buffer (pH 12) and (c) 1 M K_2CO_3 (pH 12). Each organic compound is indicated by a specific color: HMF (gray), FDCA (red), FFCA (blue), HMFCA (green), and DFF (yellow).

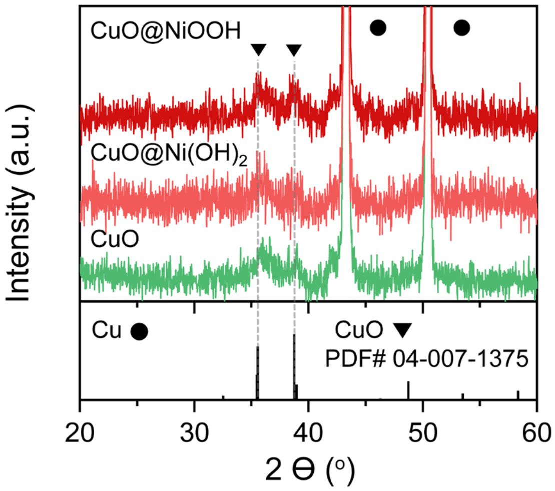

Figure S19 XRD patterns of CuO, $CuO@Ni(OH)_2$, and CuO@NiOOH.

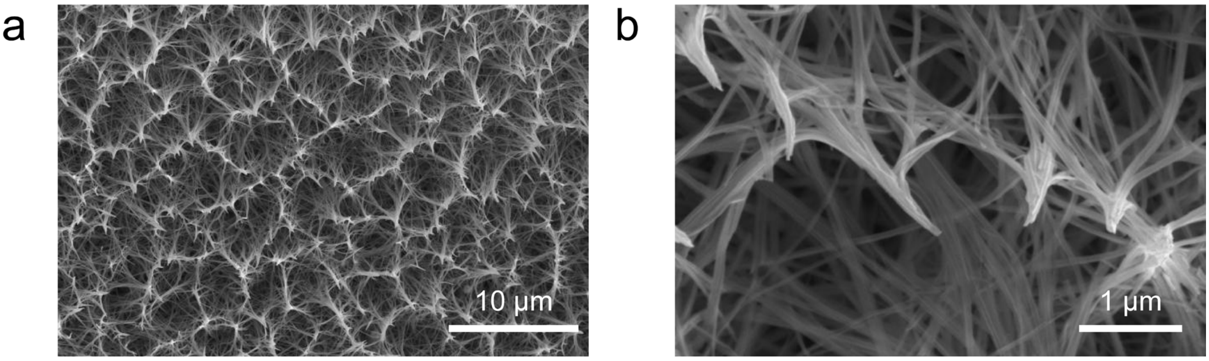

Figure S20 SEM images of CuO.

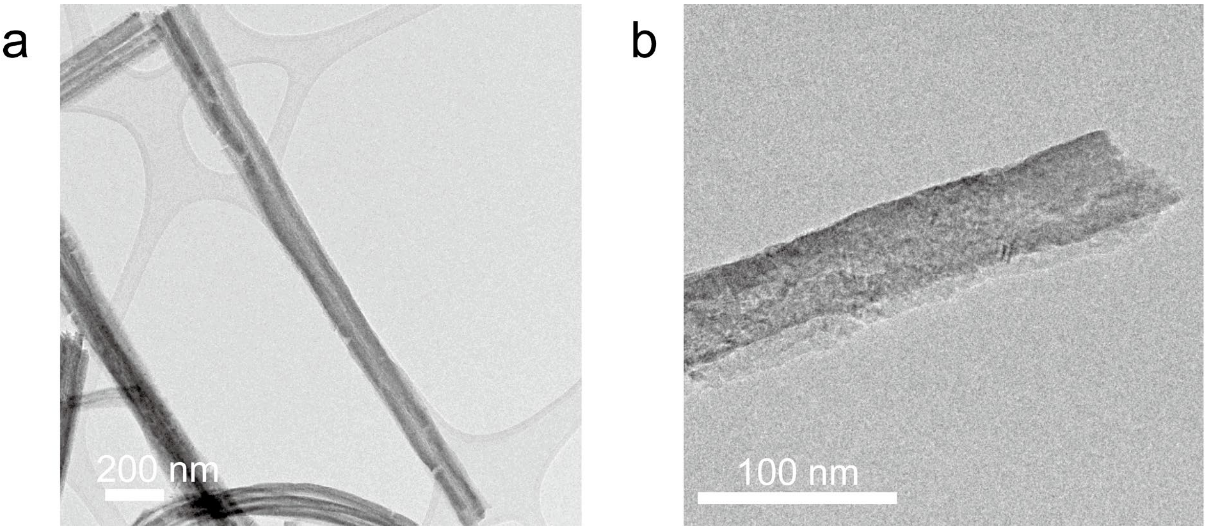

Figure S21 TEM images of CuO.

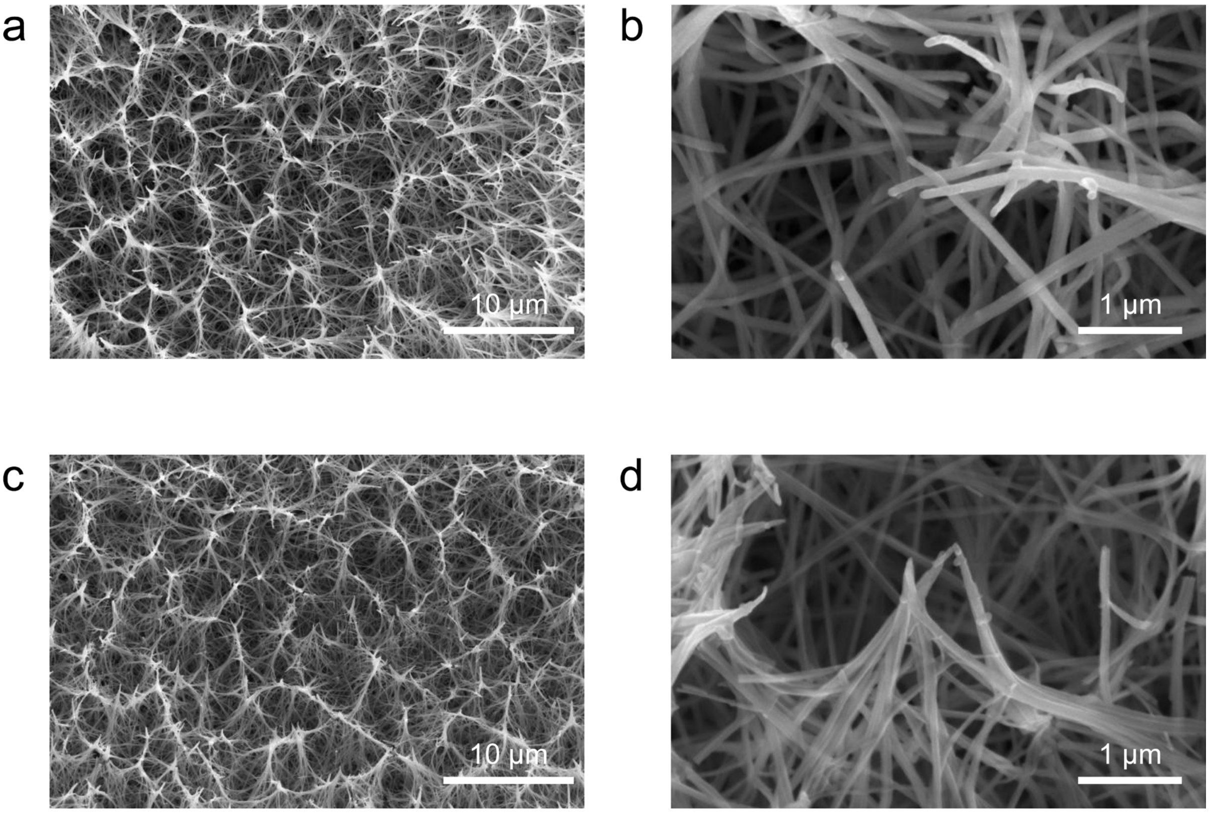

Figure S22 SEM images of (a), (b) $CuO@Ni(OH)_2$, and (c), (d) CuO@NiOOH.

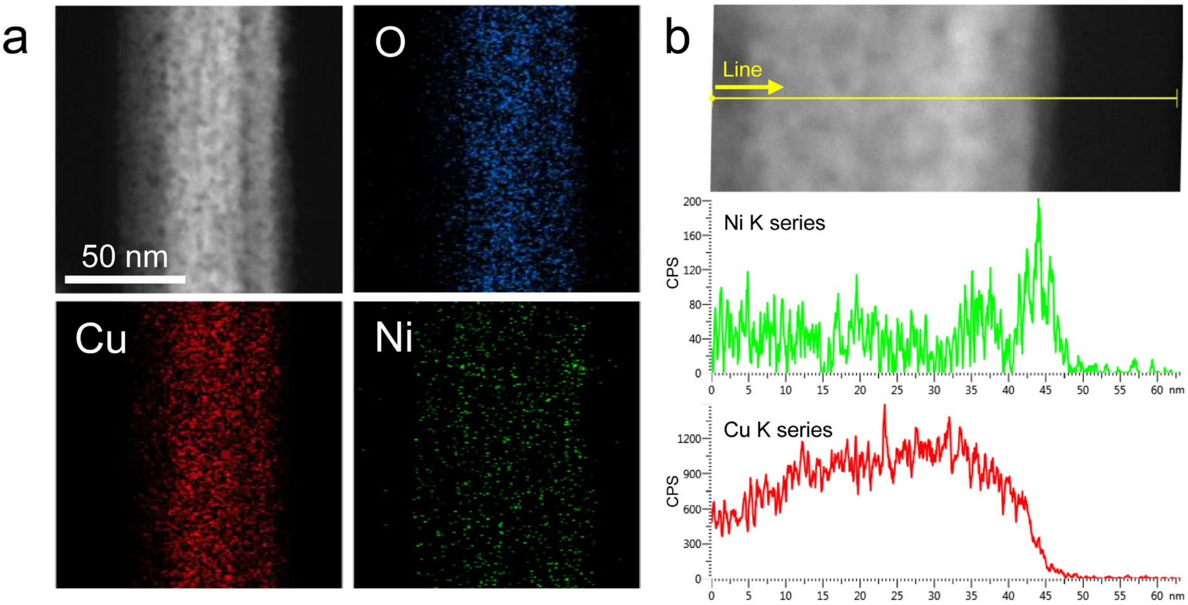

Figure S23 (a) EDS mapping of CuO@Ni$(OH)_2$. (b) Line profile of EDS for CuO@Ni$(OH)_2$.

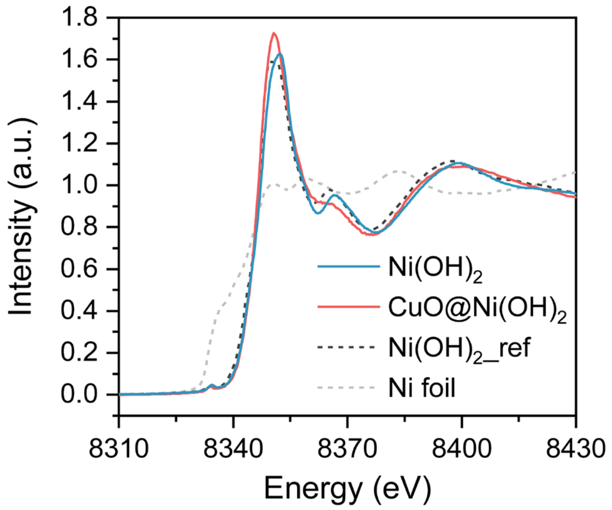

Figure S24 *ex-situ* XANES of Ni K-edge for $Ni(OH)_2$, CuO@$Ni(OH)_2$ catalysts.

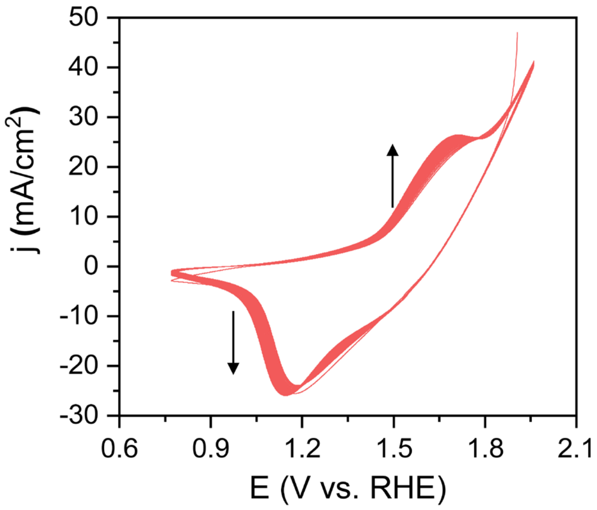

Figure S25 50 cycles of CV activation curves of CuO@Ni$(OH)_2$ to CuO@NiOOH at a scan rate of 30 mV sec^{-1}.

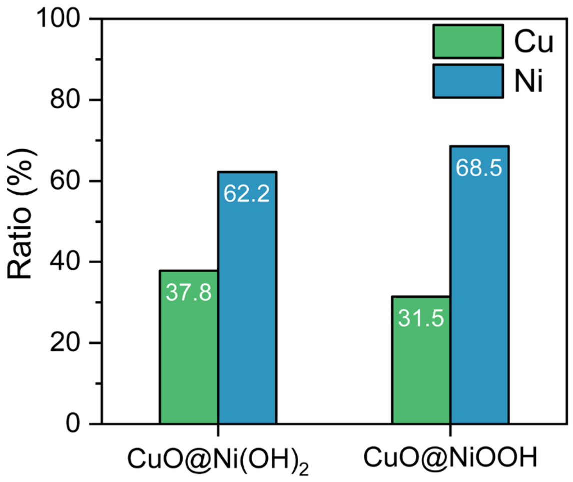

Figure S26 Cu and Ni element ratio comparison of CuO@Ni(OH)$_2$ and CuO@NiOOH based on the XPS spectra.

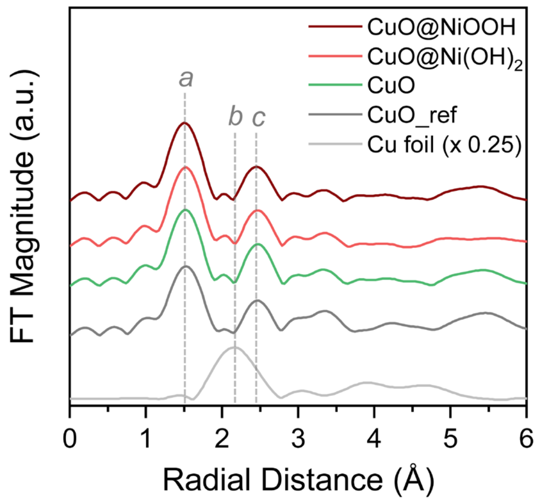

Figure S27 *ex-situ* EXAFS of Cu K-edge for CuO, CuO@Ni$(OH)_2$, CuO@NiOOH. *a, b,* and *c* peaks were derived from Cu-O in the first shell of CuO, Cu-Cu from the metallic Cu, and the convoluted peaks of Cu-O and Cu-Cu from the second shell of CuO, respectively.

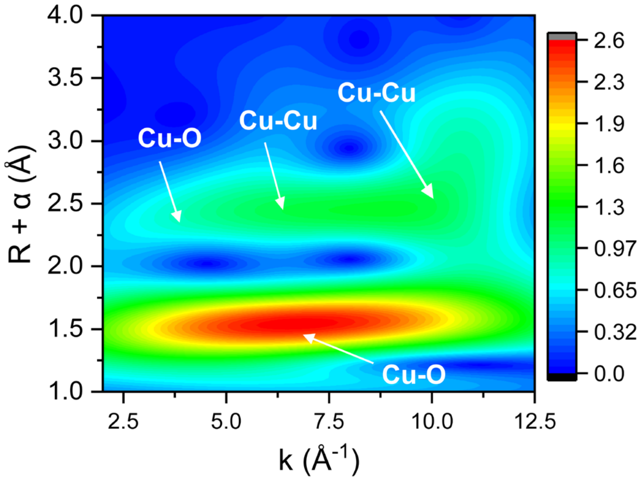

Figure S28 *ex-situ* WT-EXAFS of Cu K-edge for CuO.

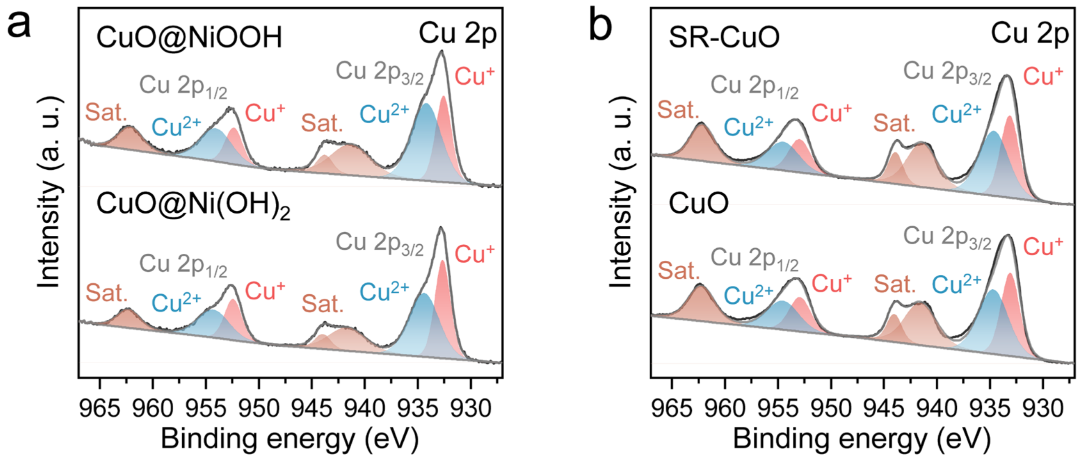

Figure S29 Cu 2p XPS of (a) CuO@Ni$(OH)_2$ and CuO@NiOOH, (b) CuO and surface reconstructed-CuO (SR-CuO). After the CV activation, the Cu^{2+}/Cu^{+} increased from 1.24 of CuO@Ni$(OH)_2$ to 1.68 of CuO@NiOOH. In contrast, the Cu^{2+}/Cu^{+} increased from 1.07 of CuO to 1.26 of SR-CuO.

To investigate the CV activation effect, we applied the same treatment of CV to the CuO electrode (SR-CuO). A more enhanced ratio was observed for CuO@NiOOH, indicating that the electronic modulation is derived from the increase of CuO/NiOOH interfaces.

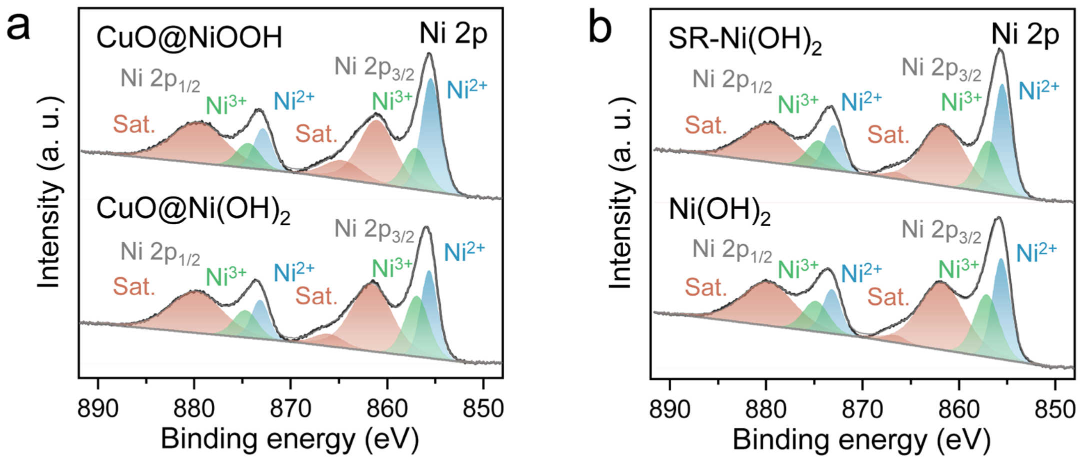

Figure S30 Ni 2p XPS of (a) CuO@Ni(OH)$_2$ and CuO@NiOOH, (b) Ni(OH)$_2$ and surface SR-Ni(OH)$_2$. After the CV activation, the Ni^{3+}/Ni^{2+} decreased from 0.99 of CuO@Ni(OH)$_2$ to 0.45 of CuO@NiOOH. In contrast, the Ni^{3+}/Ni^{2+} decreased from 0.86 of Ni(OH)$_2$ to 0.63 of SR-Ni(OH)$_2$.

Similar to the Cu 2p XPS, we also performed the CV activation using Ni(OH)$_2$. The oxidation states of Ni decreased for all electrodes due to the interaction with the phosphate anion, influencing the electronic modulation via adsorption. [22-24] The overall decrease of Ni^{3+}/Ni^{2+} for CuO@Ni(OH)$_2$/CuO@NiOOH was more featured compared to that of Ni(OH)$_2$/SR-Ni(OH)$_2$.

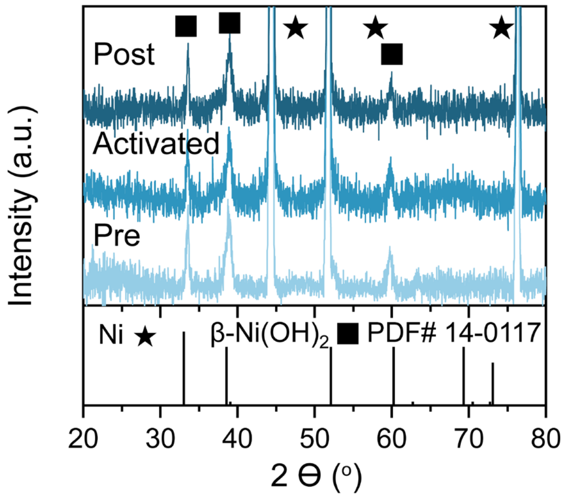

Figure S31 XRD patterns of $Ni(OH)_2$.

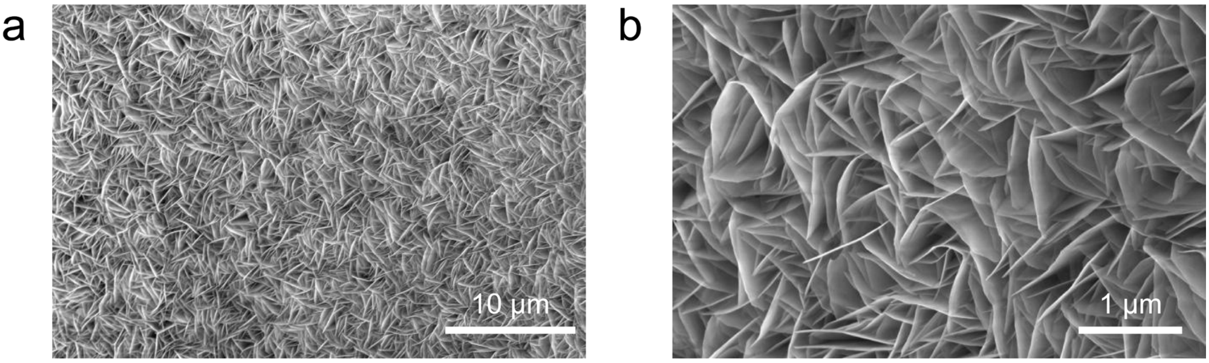

Figure S32 SEM images of $Ni(OH)_2$.

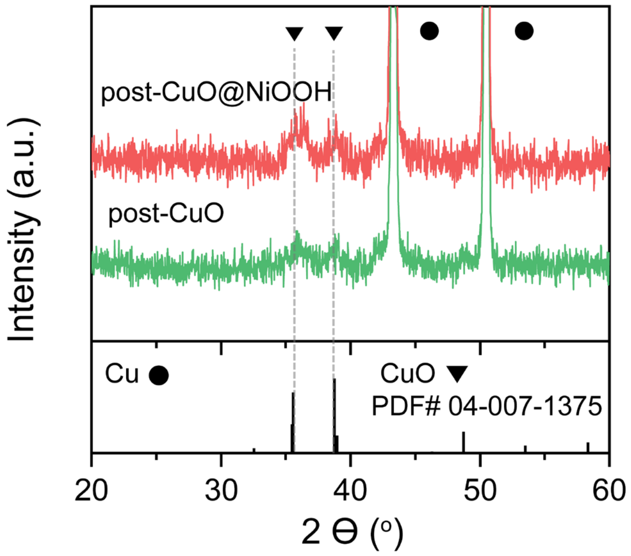

Figure S33 XRD pattern for post-catalysts of CuO@NiOOH and CuO (denoted to post-CuO@NiOOH and post-CuO).

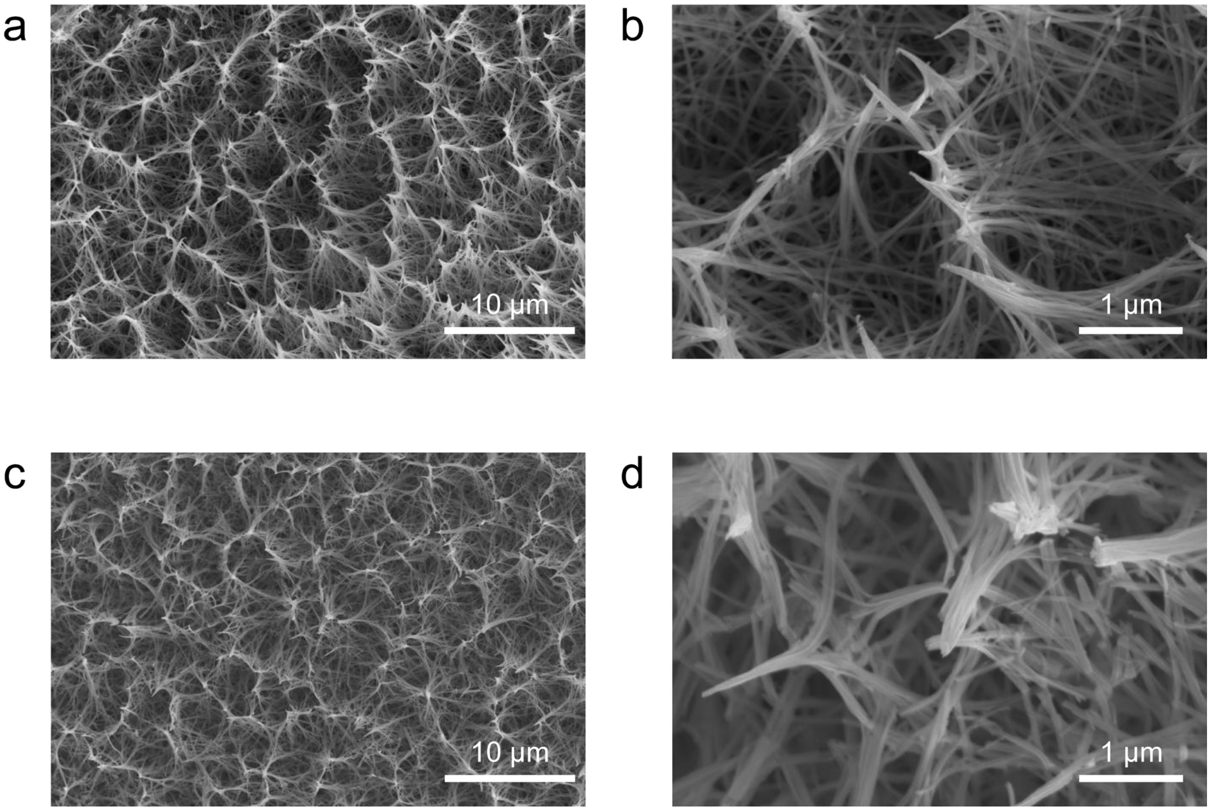

Figure S34 SEM images of (a), (b) post-CuO@NiOOH, and (c), (d) post-CuO.

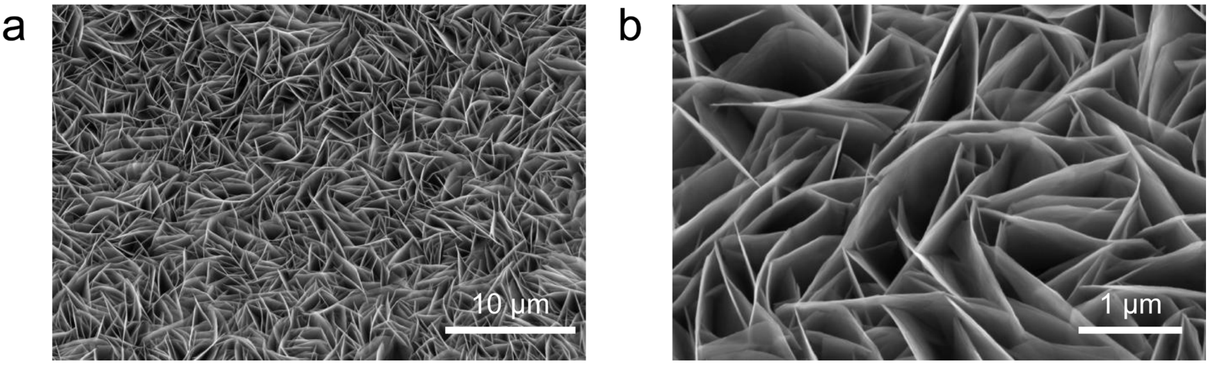

Figure S35 SEM images of post-$Ni(OH)_2$.

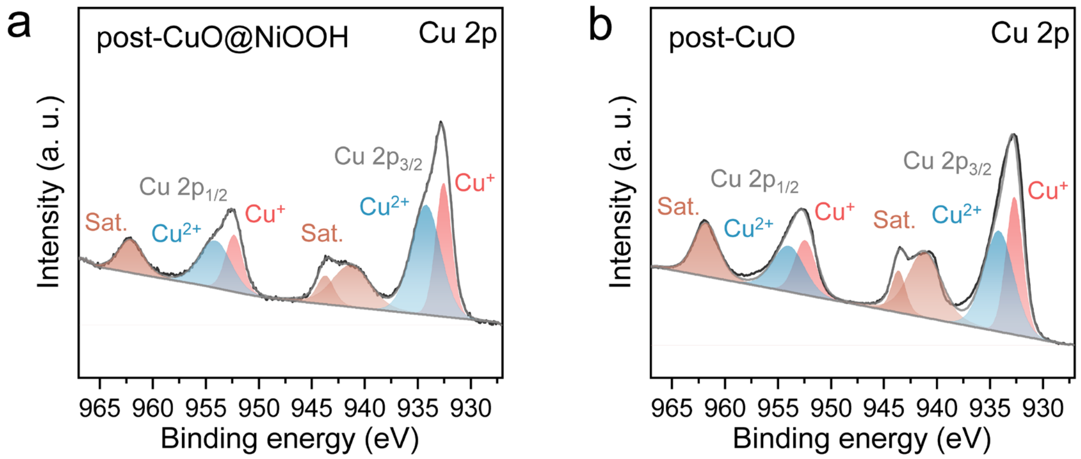

Figure S36 Cu 2p XPS of (a) post-CuO@NiOOH, and (b) post-CuO. The Cu^{2+}/Cu^{+} ratio of post-CuO@NiOOH was 1.52, and that of post-CuO was 1.29. These values were similar to CuO@NiOOH and SR-CuO, indicating that electronic structures were preserved after HMFOR.

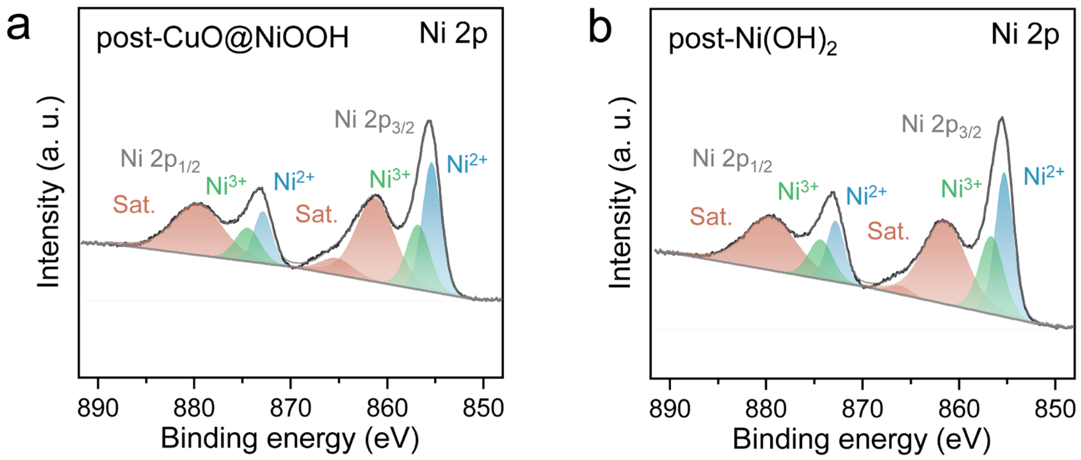

Figure S37 Ni 2p XPS of (a) post-CuO@$Ni(OH)_2$, and (b) post-$Ni(OH)_2$. The Ni^{3+}/Ni^{2+} ratio of post-CuO@NiOOH was 0.63, and that of post-$Ni(OH)_2$ was 0.74.

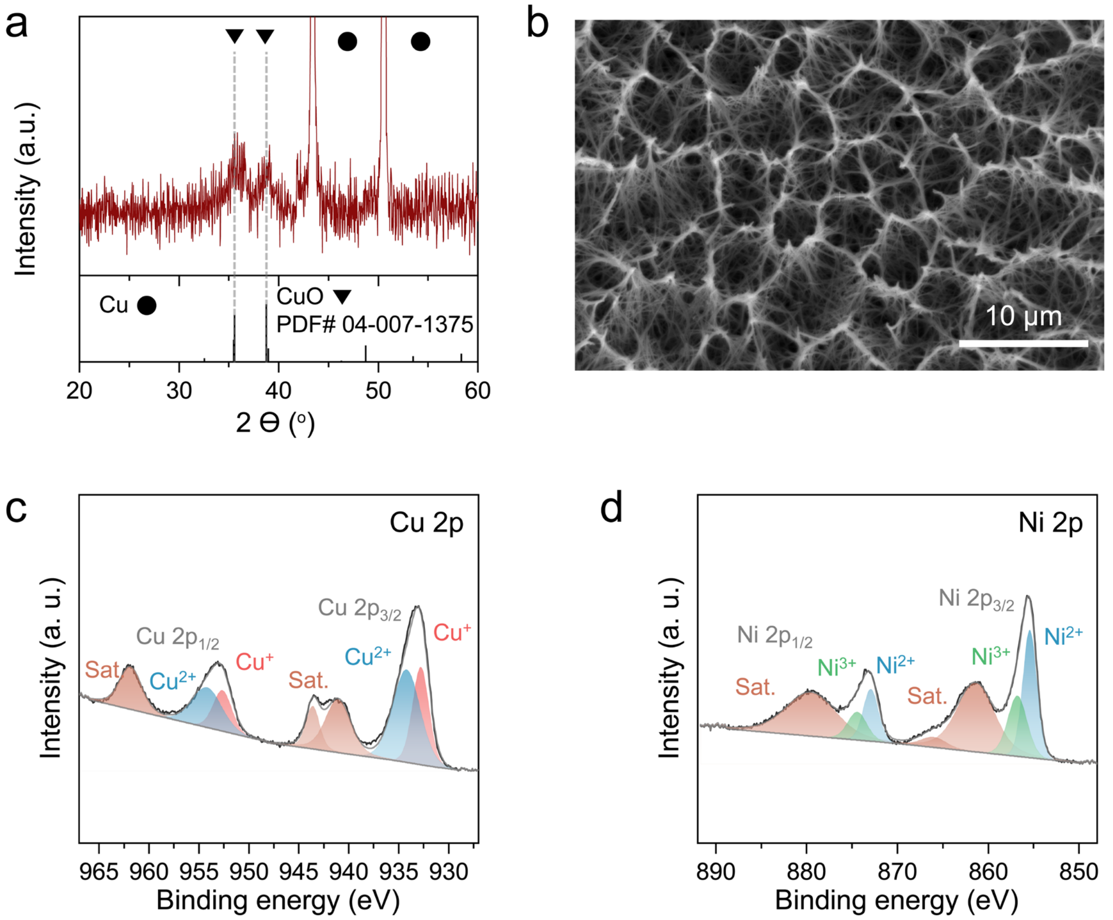

Figure S38 Characterization of CuO@NiOOH after long-term stability test. (a) XRD pattern of post-CuO@NiOOH. (b) SEM image of post-CuO@NiOOH. XPS of CuO@NiOOH for (c) Cu 2p and (d) Ni 2p. The Cu^{2+}/Cu^{+} ratio of CuO@NiOOH after long-term stability test was 1.57, and the Ni^{3+}/Ni^{2+} ratio was 0.65.

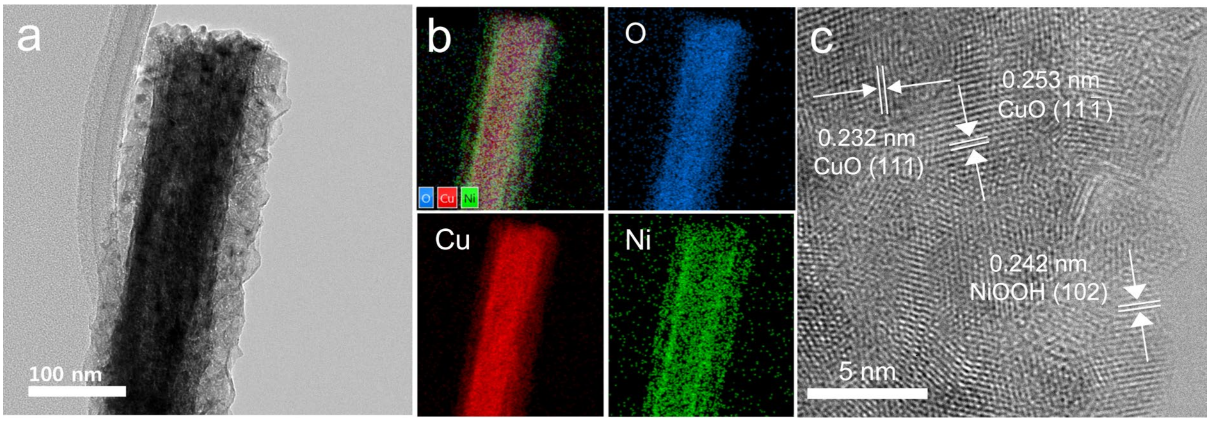

Figure S39. Characterization of CuO@NiOOH after long-term stability test. (a) TEM image, (b) EDS mapping, (c) HR-TEM image of post-CuO@NiOOH.

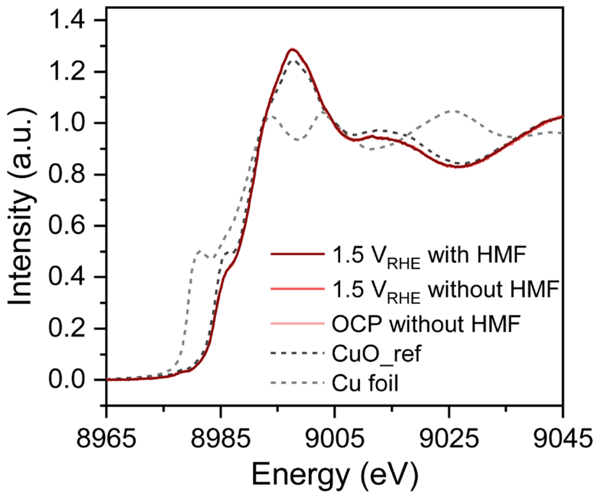

Figure S40 *in situ/operando* Cu K-edge XANES of CuO@NiOOH without/with 5 mM HMF at pH 12 under OCP and applied potential at 1.5 V vs. RHE.

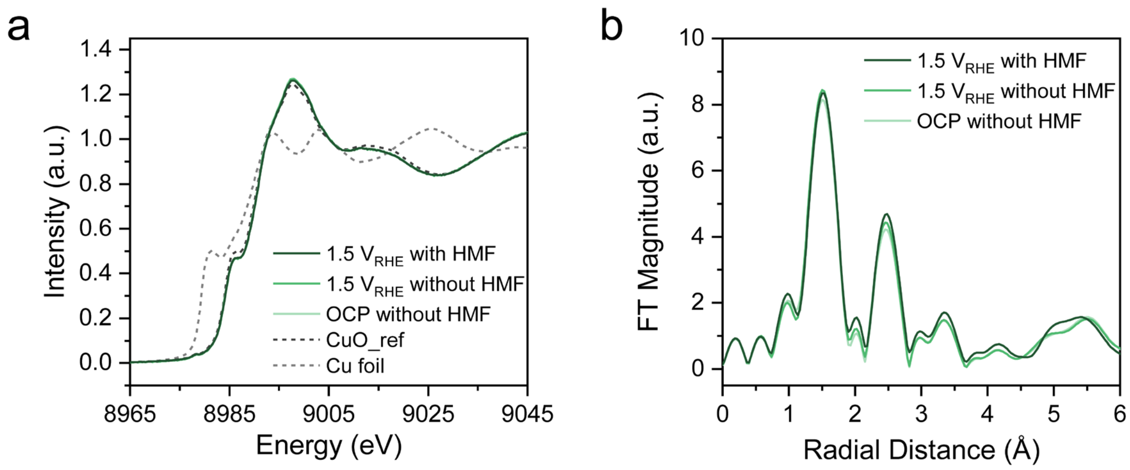

Figure S41 *in situ/operando* Cu-K edge XANES and EXAFS of CuO without/with 5 mM HMF at pH 12 under OCP and applied potential at 1.5 V vs. RHE.

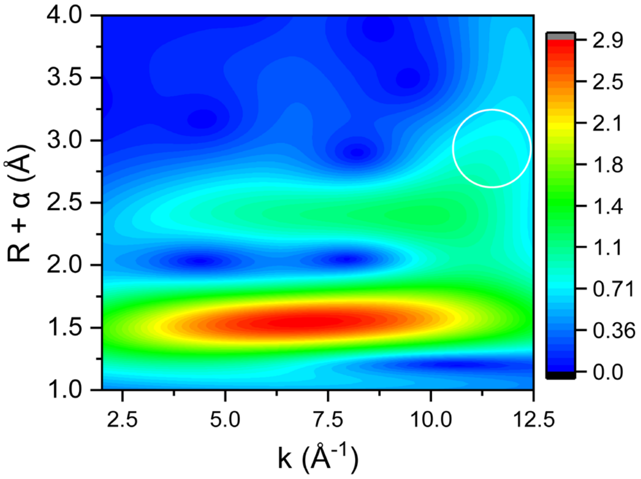

Figure S42 *in situ* WT-EXAFS of Cu K-edge using CuO@NiOOH at OCP condition.

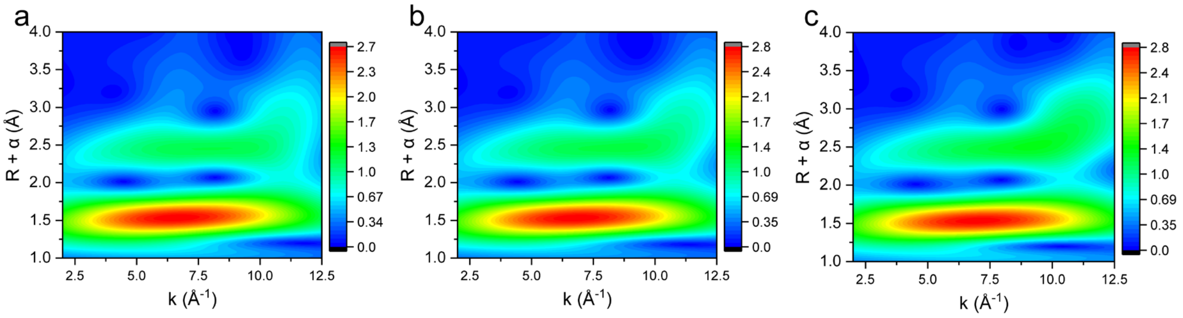

Figure S43 *in situ/operando* WT-EXAFS of CuO under (a) OCP, (b) during CA without HMF at 1.5 V vs. RHE, (c) during CA with 5 mM HMF at 1.5 V vs. RHE

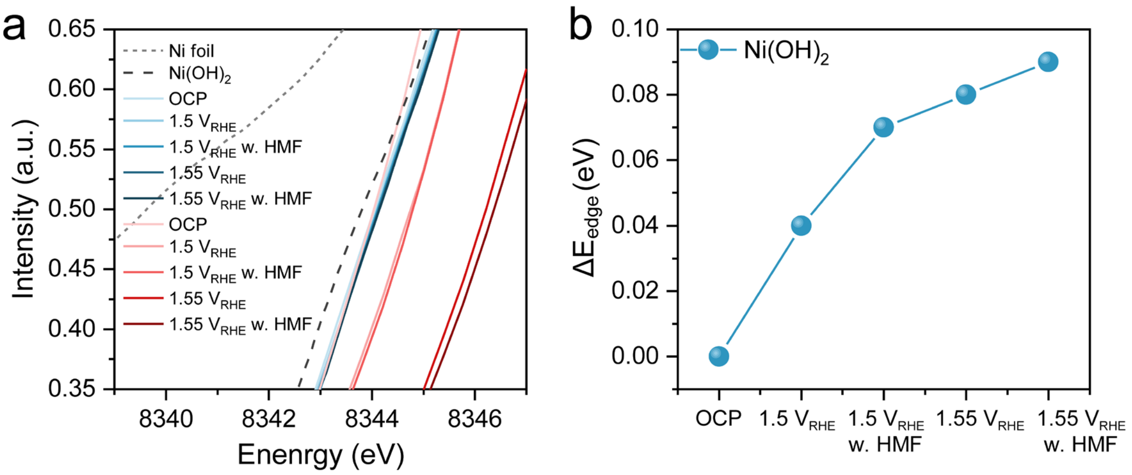

Figure S44 (a) Enlarged plot of *in situ/operando* XANES for Ni K-edge based on the CuO@NiOOH, and $Ni(OH)_2$ catalyst at OCP, 1.5 V vs. RHE without/with 5 mM HMF, and 1.55 V vs. RHE without/with 5 mM HMF. Plot of ΔE_{edge} versus *in situ/operando* condition of OCP, 1.5 V vs. RHE without HMF, with 5 mM HMF (w. HMF), and 1.55 V vs. RHE without/with 5 mM HMF using $Ni(OH)_2$ catalyst.

Linear Combination Fitting (LCF) Results

To investigate the phase transition behavior in response to the applied potential, we performed linear combination fitting (LCF) based on the *in situ/operando* Ni K-edge XAS. The XANES of Ni K-edge exhibited the increased absorption energy at the higher potential in the case of CuO@NiOOH (**Figure S44**). The energy shift would be derived from the phase conversion of $Ni(OH)_2$ to NiOOH, in which Ni^{3+} is abundant. However, the NiOOH is not commercially available, and it is unstable under ambient conditions, resulting in the $Ni(OH)_2$/NiOOH mixture. To clarify the relationship between XANES results and phase transition, EXAFS fitting was conducted using CuO@NiOOH results under applied potential at 1.55 V vs. RHE without HMF (**Figure S45, Table S2**). EXAFS of CuO@NiOOH at 1.55 V vs. RHE without HMF closely matched the theoretical crystallography of NiOOH. Therefore, we considered CuO@NiOOH under that condition as the NiOOH reference. Using commercially purchased $Ni(OH)_2$, and CuO@NiOOH results at 1.55 V vs. RHE without HMF as $Ni(OH)_2$, and NiOOH reference, respectively, LCF was conducted (**Figures S46-S47**). In the case of CuO@NiOOH, the OCP condition showed that the majority phase was $Ni(OH)_2$. At 1.5 V vs. RHE without/with 5 mM HMF, a mixture of $Ni(OH)_2$ and NiOOH was observed based on the LCF, indicating the phase transition from $Ni(OH)_2$ to NiOOH in response to the applied potential. At a higher potential of 1.55 V vs. RHE with 5 mM HMF, the CuO@NiOOH exhibited that the NiOOH phase was predominantly featured. However, for the $Ni(OH)_2$ catalyst, the ratio between $Ni(OH)_2$ and NiOOH was 88.3 and 11.7, and this ratio remained unchanged even under applied potential at 1.5 V vs. RHE, and 1.55 V vs. RHE (**Figure S48**). These results indicate that the $Ni(OH)_2$ has poor phase transition ability between $Ni(OH)_2$ and NiOOH.

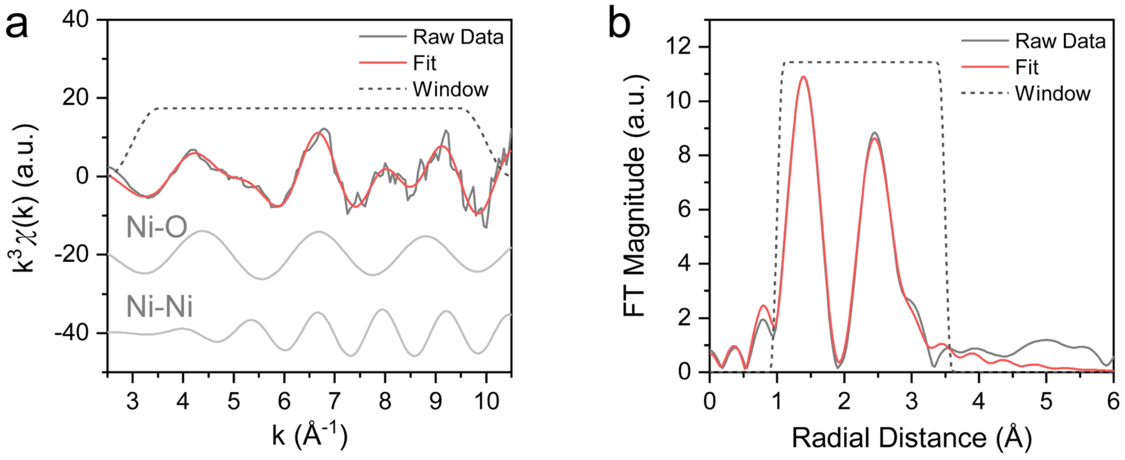

Figure S45 EXAFS fitting results of CuO@NiOOH at 1.55 V vs. RHE without HMF based on (a) k-space, (b) R-space.

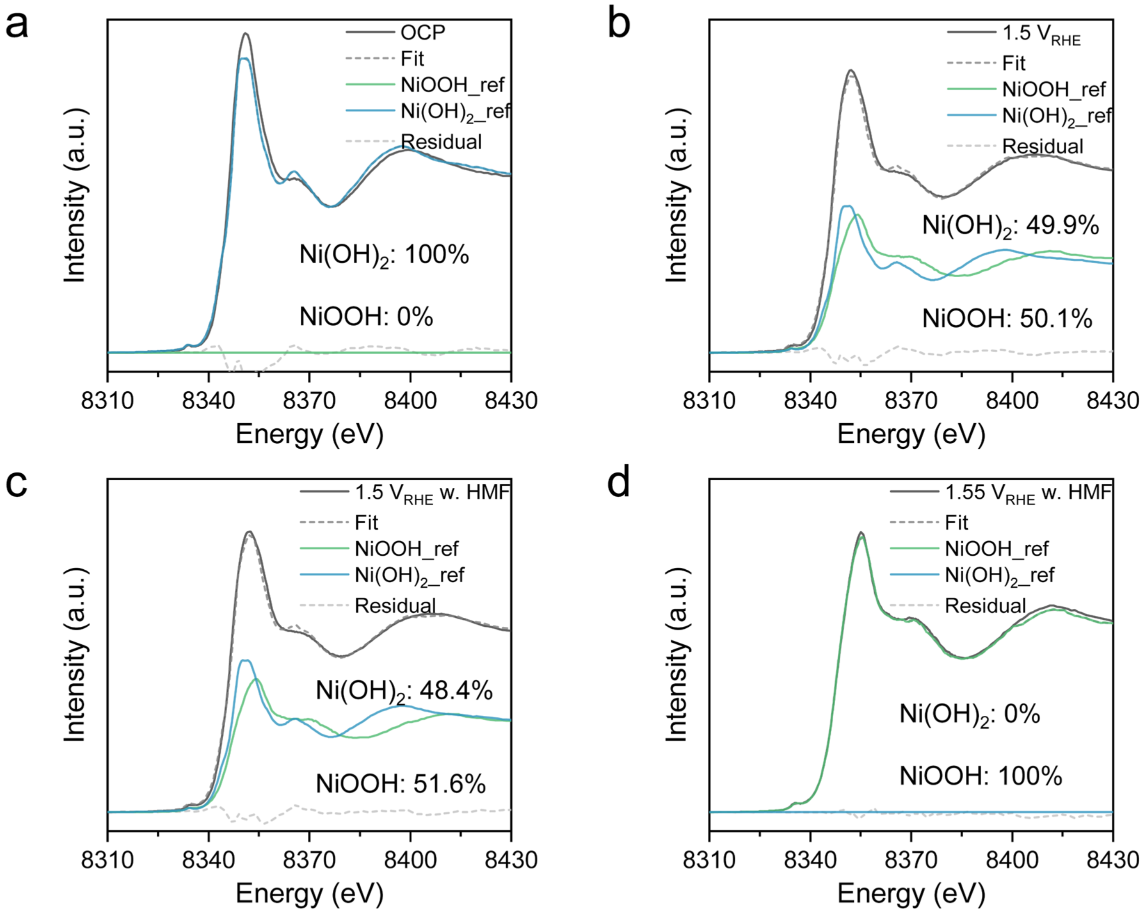

Figure S46 LCF results of CuO@NiOOH catalyst (a) OCP, (b) 1.5 V vs. RHE without HMF, (c) 1.5 V vs. RHE with 5 mM HMF, and (d) 1.55 V vs. RHE with 5 mM HMF condition. Commercially purchased $Ni(OH)_2$, and CuO@NiOOH results at 1.55 V vs. RHE without HMF were utilized as $Ni(OH)_2$, and NiOOH reference ($Ni(OH)_2$_ref, and NiOOH_ref, respectively).

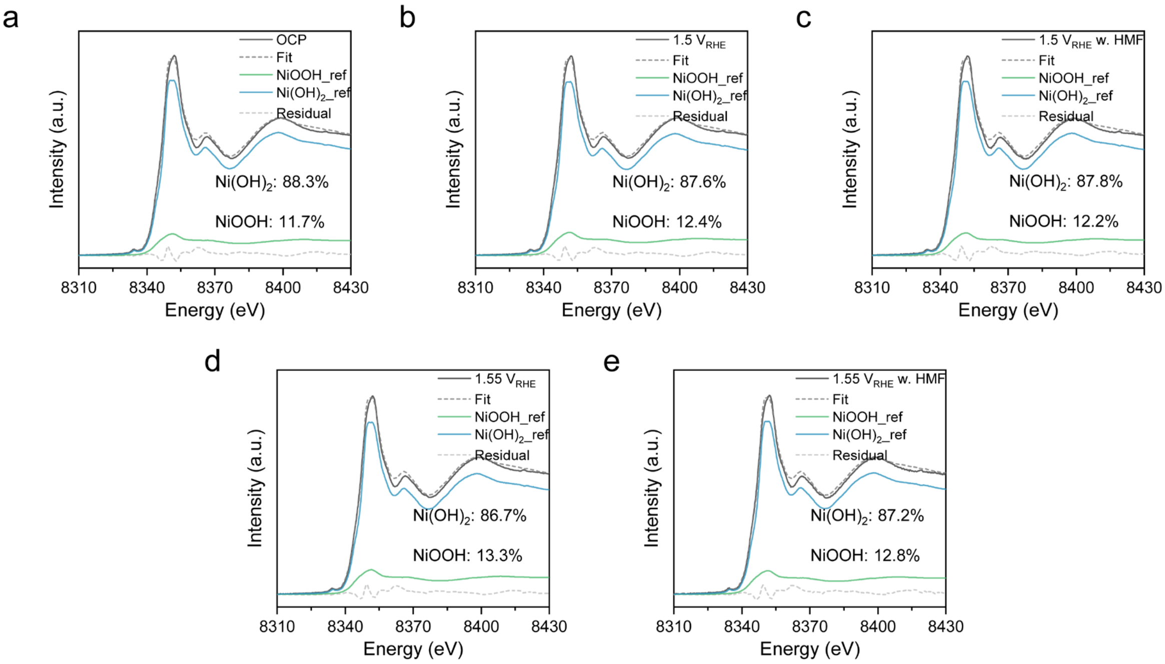

Figure S47 LCF results of $Ni(OH)_2$ catalyst (a) OCP, (b) 1.5 V vs. RHE without HMF, (c) 1.5 V vs. RHE with 5 mM HMF, (d) 1.55 V vs. RHE without HMF, and (e) 1.55 V vs. RHE with 5 mM HMF condition. Commercially purchased $Ni(OH)_2$, and CuO@NiOOH results at 1.55 V vs. RHE without HMF were utilized as $Ni(OH)_2$, and NiOOH reference ($Ni(OH)_2$_ref, and NiOOH_ref, respectively).

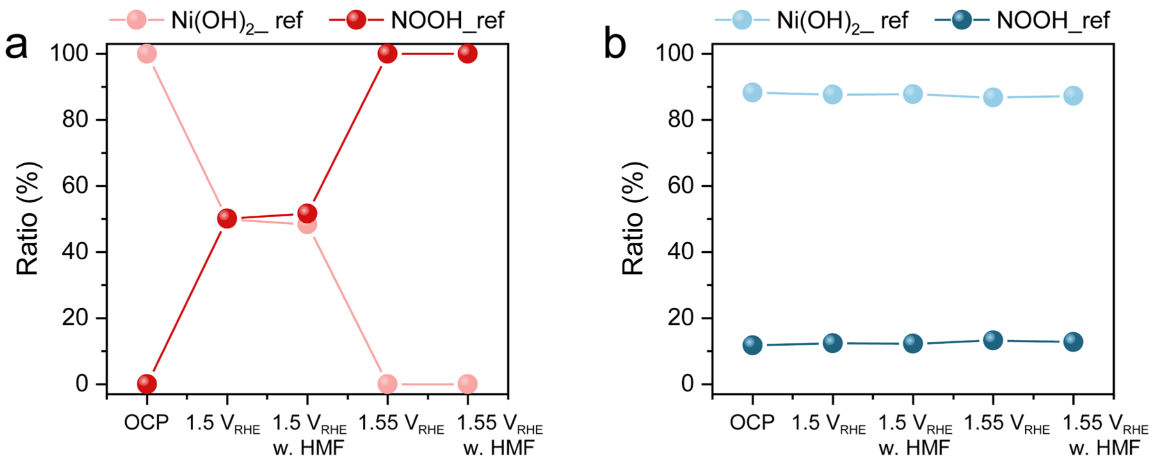

Figure S48 Plot of $Ni(OH)_2$ and NiOOH ratio based on the LCF results versus *in situ/operando* condition of (a) CuO@NiOOH, (b) $Ni(OH)_2$ catalyst.

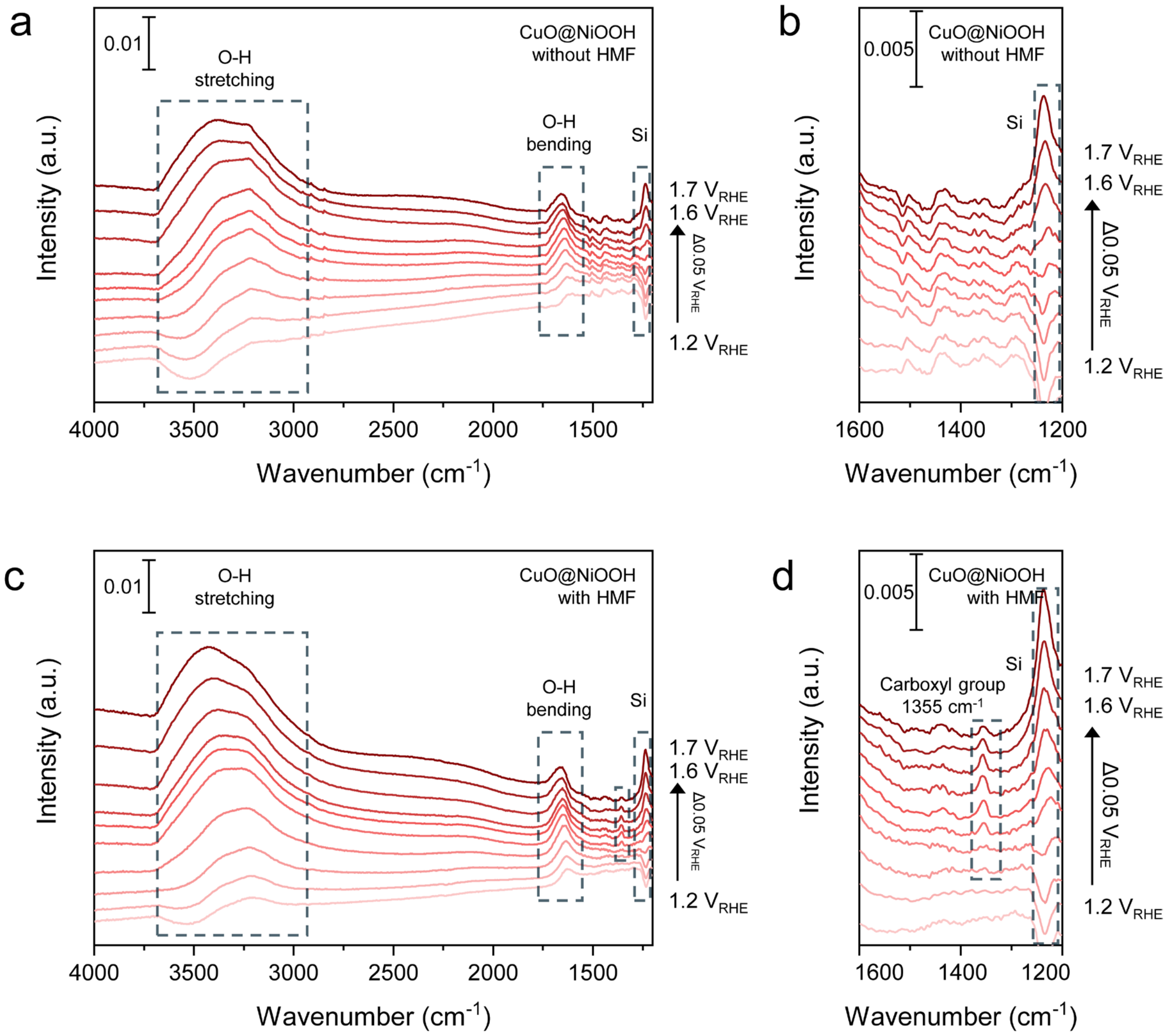

Figure S49 *Operando* ATR-SEIRAS of CuO@NiOOH. (a) Full spectral region without HMF, (b) low wavenumber region without HMF, (c) Full spectral region with 5 mM HMF, and (d) low wavenumber region with 5 mM HMF.

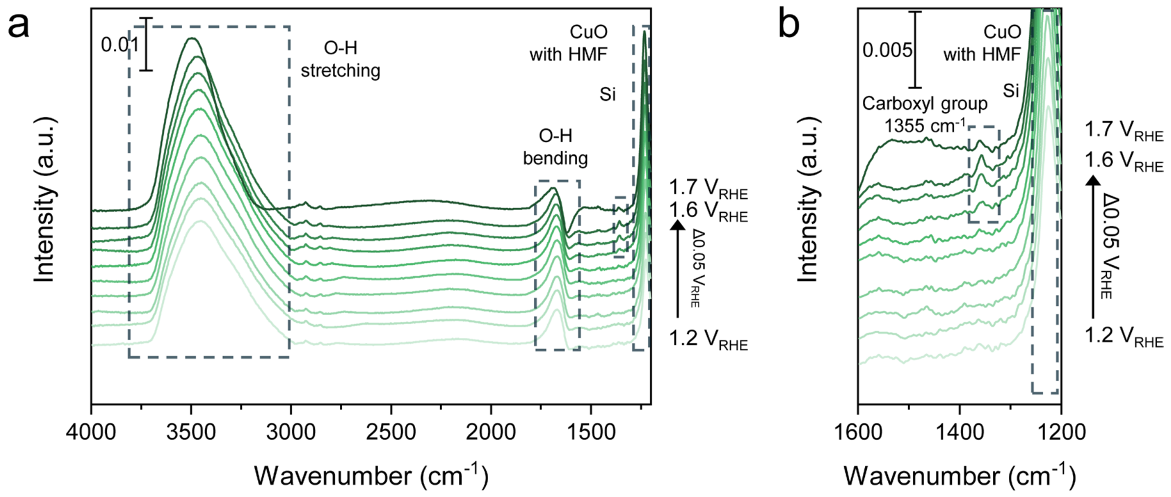

Figure S50 *Operando* ATR-SEIRAS of CuO. (a) Full spectral region with 5 mM HMF, and (b) low wavenumber region with 5 mM HMF.

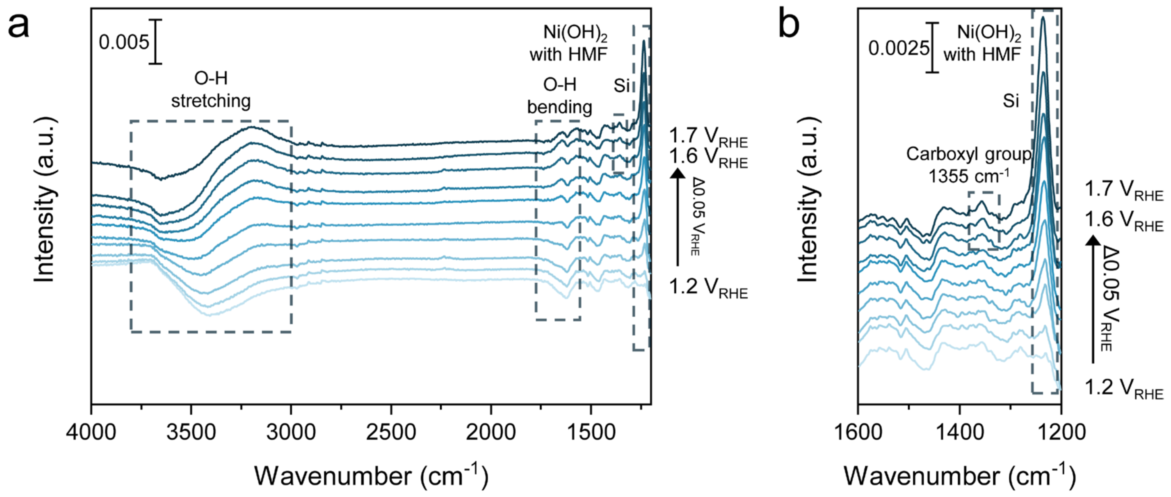

Figure S51 *Operando* ATR-SEIRAS of $Ni(OH)_2$. (a) Full spectral region with 5 mM HMF, and (b) low wavenumber region with 5 mM HMF.

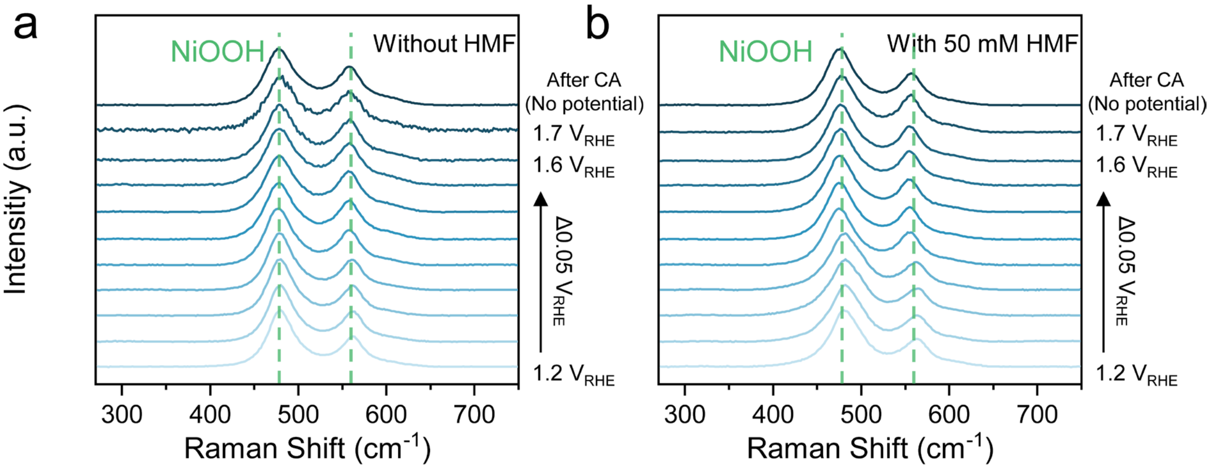

Figure S52 *Operando* Raman spectra of $Ni(OH)_2$ depending on the applied potential from 1.2 V vs. RHE to 1.7 V vs. RHE (a) without HMF and (b) 50 mM HMF conditions at pH 12.

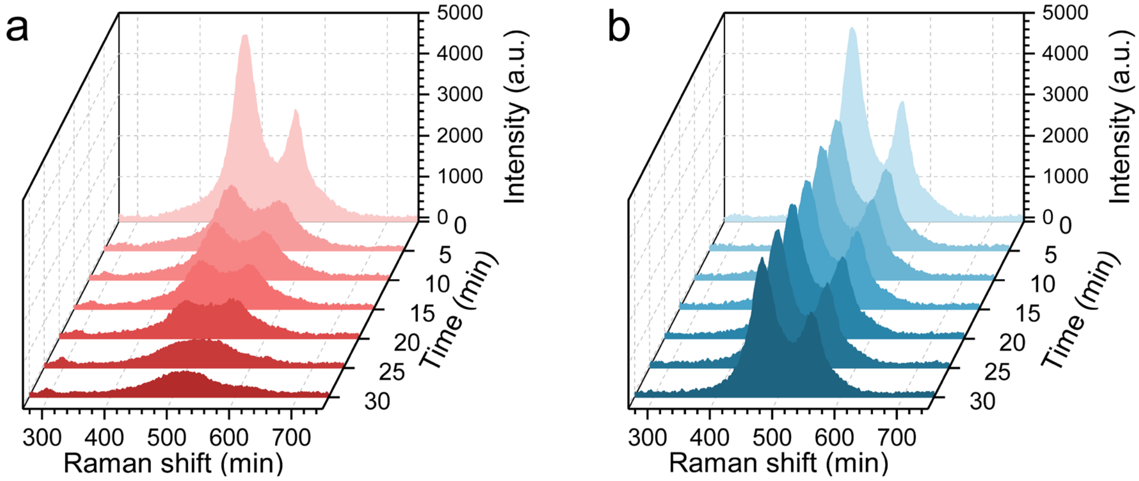

Figure S53 Compilation of TRS each 5 min tracking the Raman at OCP after stopping the applied potential at 1.45 V vs. RHE for 30 min with 50 mM HMF at pH 12 using (a) CuO@NiOOH, (b) $Ni(OH)_2$

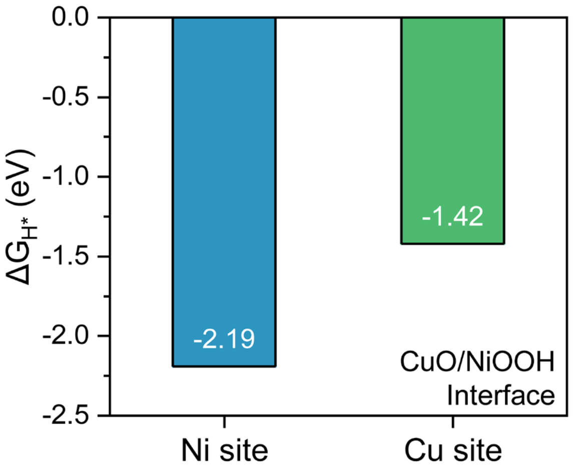

Figure S54 H adsorption energy depending on the Cu, Ni site of CuO/NiOOH interface model.

Table S1 Comparison of HMFOR performance of CuO@NiOOH catalyst with other HMFOR results.

Electrode	HMF concentration	Electrolyte	Applied potential (vs. RHE)	HMF conversion	FDCA yield	F.E.	Ref
CuO@NiOOH	5 mM	0.5 M PB[a] (pH 12)	1.45 V	>99.9%	96.9%	96.5%	This Work
CuO@NiOOH	5 mM	0.5 M PB (pH 12)	1.5 V	>99.9%	98.6%	99.2%	This Work
CuO@NiOOH	5 mM	1 M KOH	1.39 V	>99.9%	98.8%	98%	This Work
CuO@NiOOH	50 mM	0.5 M PB (pH 12)	2.0 V (2 electrode)	>99.9%	96.5%	96.2%	This Work
Co_3O_4/CuPc	5 mM	0.5 M $KHCO_3$ (pH 7.2)	1.5 V	80%	18%	NA	[25]
pCoHA-Ru	100 mM	1 M $KHCO_3$ (pH 8.3)	1.4 V	~100	92.1%[b]	NA	[26]
CoO_x-CrO_x	100 mM	1 M CB[c] (pH 11)	1.5 V	100%	89%	90%	[27]
Ni/NiOOH foam	0.65 M	0.1 M Na_2SO_4 (pH 12)	1.55 V	100%	89%	80%	[28]
NiO/CF	40 mM	0.1 M KOH	1.55 V	>99%	97.99%	93.6%	[29]
Treated NiNPs/GO-Ni-foam	5 mM	0.1 M KOH	500 mV (vs. Ag/AgCl)	96.8%	86.9%	94.8%	[30]
β-NiNS/NF	5 mM	0.1 M KOH	1.485 V	98.1%	76.42%	72.27%	[31]
Cu NPs	4.2 mM	0.1 M KOH	1.47 V	98.5%	94.0%	94.1%	[32]
Co_2Fe_1-1.8@NiF	5 mM	0.1 M KOH	1.535 V	100%	94.0%	91.%	[33]
Ni/CP-1.5	5 mM	0.1 M KOH	1.36	~100%	99.1%	99.1%	[34]
NF@Co_3O_4/CeO_2	10 mM	1 M KOH	1.4 V	98.0%	94.5%	97.5%	[35]
SC-MHEO	10 mM	1 M KOH	1.435 V	99.7%	97.9%	97.7%	[36]
CuO-PdO	10 mM	1 M KOH	1.35 V	99.5%	96.2%	93.7%	[37]
Ni-Cu/NF	50 mM	1 M KOH	1.45 V	~100%	99.67%	99.51%	[38]

[a]Phosphate buffer

[b]Two-step process, 1) HMF to FFCA at room temperature, 2) FFCA to FDCA at 60 °C

[c]Carbonate buffer

Table S2. EXAFS fitting results of CuO@NiOOH

**Condition**	***k* range ($Å^{-1}$)**	***R* range (Å)**	**Scattering**	**CN[a]**	**R (Å)[b]**	**σ^2 (X 10^3 $Å^{-2}$)[c]**	**ΔE0 (eV)**	***R* factor**
CuO@NiOOH 1.55 V without HMF	3-10	1-3.4	Ni-O	6.0 (± 0.6)	1.870 (± 0.006)	3.2 (± 0.9)	-5.0 (± 1.1)	0.3%
			Ni-Ni	5.6 (± 0.9)	2.821 (± 0.006)	5.0 (± 1.2)		

S_0^2: 0.71 (based on the reference materials)

Table S3 Calculated Gibbs free energy for each elementary step of HMF oxidation in the DAOM and GDM pathways.

Reaction Coordinate		CuO/NiOOH		CuO		NiOOH	
		**G (eV)**	**ΔG (eV)**	**G (eV)**	**ΔG (eV)**	**G (eV)**	**ΔG (eV)**
HMF → DFF	HMF $_{adsorption}$ ($C_6H_6O_3^*$)	-1.65	-1.65	-1.05	-1.05	-1.15	-1.15
	($C_6H_6O_3^*$+OH^- → $C_6H_5O_3^*$+H_2O+1e^-)	-1.75	-0.10	-1.30	-0.25	-1.73	-0.58
	DFF$_{formation}$ ($C_6H_5O_3^*$+OH^- → $C_6H_4O_3^*$+2H_2O+2e^-)	-0.62	1.13	-0.26	1.04	-0.51	1.22
HMF → HMFCA	HMF $_{adsorption}$ ($C_6H_6O_3^*$)	-1.65	-1.65	-1.05	-1.05	-1.15	-1.15
	GDM. ($C_6H_6O_3^*$+OH^- → $C_6H_7O_4^*$+1e^-)	1.16	2.82	-0.19	0.86	0.73	1.88
	DAOM. ($C_6H_6O_3^*$+OH^- → $C_6H_5O_3^*$+H_2O+1e^-)	-2.88	-1.23	-3.49	-2.44	-1.98	-0.83
	GDM. HMFCA $_{formation}$ ($C_6H_7O_4^*$+OH^- → $C_6H_6O_4^*$+H_2O+2e^-)	-1.34	-2.50	-1.20	-1.01	-1.57	-2.30
	DAOM. HMFCA $_{formation}$ ($C_6H_5O_3^*$+OH^- → $C_6H_6O_4^*$+H_2O+2e^-)	-1.34	1.54	-1.20	2.29	-1.57	0.41
DFF → FFCA	DFF$_{formation}$ ($C_6H_4O_3^*$+2H_2O+2e^-)	-0.62	-	-0.26	-	-0.51	-
	GDM. ($C_6H_4O_3^*$+OH^- → $C_6H_5O_4^*$+2H_2O+3e^-)	0.44	1.06	0.53	0.79	1.21	1.72
	DAOM. ($C_6H_4O_3^*$+OH^- → $C_6H_3O_3^*$+3H_2O+3e^-)	-2.94	-2.32	-2.85	-2.59	-1.39	-0.88
	GDM. FFCA $_{formation}$ ($C_6H_5O_4^*$+OH^- → $C_6H_4O_4^*$+3H_2O+4e^-)	-1.21	-1.65	-0.67	-1.20	-0.61	-1.82
	DAOM. FFCA $_{formation}$ ($C_6H_3O_3^*$+OH^- → $C_6H_4O_4^*$+3H_2O+4e^-)	-1.21	1.73	-0.67	2.18	-0.61	0.78
HMFCA → FFCA	HMFCA $_{formation}$ ($C_6H_6O_4^*$+H_2O+2e^-)	-1.34	-	-1.20	-	-1.57	-
	($C_6H_6O_4^*$+OH^- → $C_6H_5O_4^*$+2H_2O+3e^-)	-2.15	-0.81	-1.89	-0.69	-1.92	-0.35
	FFCA $_{formation}$ ($C_6H_5O_4^*$+OH^- → $C_6H_4O_4^*$+3H_2O+4e^-)	-1.21	0.94	-0.67	1.22	-0.61	1.31
FFCA → FDCA	FFCA $_{formation}$ ($C_6H_4O_4^*$+3H_2O+4e^-)	-1.21	-	-0.67	-	-0.61	-
	GDM. ($C_6H_4O_4^*$+OH^- → $C_6H_5O_5^*$+3H_2O+5e^-)	0.16	1.37	0.56	1.23	1.25	1.86
	DAOM. ($C_6H_4O_4^*$+OH^- → $C_6H_3O_4^*$+4H_2O+5e^-)	-2.67	-1.46	-3.15	-2.48	-1.36	-0.75
	GDM. FDCA $_{formation}$ ($C_6H_5O_5^*$+OH^- → $C_6H_4O_5^*$+4H_2O+6e^-)	-1.75	-1.59	-0.14	-0.70	-0.45	-1.70
	DAOM. FDCA $_{formation}$ ($C_6H_3O_4^*$+OH^- → $C_6H_4O_5^*$+4H_2O+6e^-)	-1.75	0.92	-0.14	3.01	-0.45	0.91

Reference

[1] X. Wen, W. Zhang, S. Yang, *Langmuir* **2003**, *19*, 5898-5903.

[2] Z. Li, M. Shao, L. Zhou, R. Zhang, C. Zhang, J. Han, M. Wei, D. G. Evans, X. Duan, *Nano Energy* **2016**, *20*, 294-304.

[3] X. Xia, J. Tu, Y. Zhang, X. Wang, C. Gu, X.-b. Zhao, H. J. Fan, *ACS Nano* **2012**, *6*, 5531-5538.

[4] K. Qiu, M. Lu, Y. Luo, X. Du, *J. Mater. Chem. A* **2017**, *5*, 5820-5828.

[5] X. Yi, H. Sun, N. Robertson, C. Kirk, *Sustainable Energy Fuels* **2021**, *5*, 5236-5246.

[6] D. W. Barnum, *J. Chem. Educ.* **1999**, *76*, 938.

[7] M. K. Goetz, M. T. Bender, K.-S. Choi, *Nat. Commun.* **2022**, *13*, 5848.

[8] J. Choi, S. Yoo, P. M. Nguyen, E. Lee, H. Shin, Y. J. Hwang, *ACS Catal.* **2025**, *15*, 6906-6917.

[9] E. Lee, J. H. Kim, J. Choi, Y. Hong, D. Shin, H. Yun, J. Kim, G. Bak, S. Hong, Y. J. Hwang, *J. Mater. Chem. A* **2023**, *11*, 16559-16569.

[10] J. Woo, J. Choi, J. Choi, M. Y. Lee, E. Kim, S. Yun, S. Yoo, E. Lee, U. Lee, D. H. Won, *Adv. Funct. Mater.* **2025**, *35*, 2413951.

[11] D. S. Sholl, J. A. Steckel, *Density functional theory: a practical introduction*, John Wiley & Sons, **2011**.

[12] G. Kresse, J. Furthmüller, *Phys. Rev. B* **1996**, *54*, 11169.

[13] J. P. Perdew, K. Burke, M. Ernzerhof, *Phys. Rev. Lett.* **1996**, *77*, 3865.

[14] S. Grimme, S. Ehrlich, L. Goerigk, *J. Comput. Chem.* **2011**, *32*, 1456-1465.

[15] S. Grimme, J. Antony, S. Ehrlich, H. Krieg, *J. Chem. Phys.* **2010**, *132*.

[16] A. Živković, A. Roldan, N. H. De Leeuw, *Phys. Rev. B* **2019**, *99*, 035154.

[17] Z. He, J. Hwang, Z. Gong, M. Zhou, N. Zhang, X. Kang, J. W. Han, Y. Chen, *Nat. Commun.* **2022**, *13*, 3777.

[18] H. J. Monkhorst, J. D. Pack, *Phys. Rev. B* **1976**, *13*, 5188.

[19] J. K. Nørskov, J. Rossmeisl, A. Logadottir, L. Lindqvist, J. R. Kitchin, T. Bligaard, H. Jonsson, *J. Phys. Chem. B* **2004**, *108*, 17886-17892.

[20] H. Liu, N. Agrawal, A. Ganguly, Y. Chen, J. Lee, J. Yu, W. Huang, M. M. Wright, M. J. Janik, W. Li, *Energy Environ. Sci.* **2022**, *15*, 4175-4189.

[21] J. Carro, P. Ferreira, L. Rodríguez, A. Prieto, A. Serrano, B. Balcells, A. Ardá, J. Jiménez-Barbero, A. Gutiérrez, R. Ullrich, *The FEBS journal* **2015**, *282*, 3218-3229.

[22] M. Yu, J. Li, F. Liu, J. Liu, W. Xu, H. Hu, X. Chen, W. Wang, F. Cheng, *J. Energy. Chem.* **2022**, *72*, 361-369.

[23] D. Zhou, Z. Cai, Y. Bi, W. Tian, M. Luo, Q. Zhang, Q. Zhang, Q. Xie, J. Wang, Y. Li, *Nano Research*

2018, *11*, 1358-1368.

[24] H. Komiya, K. Obata, T. Honma, K. Takanabe, *J. Mater. Chem. A* **2024**, *12*, 3513-3522.

[25] Y. Tao, S. Fan, X. Li, J. Yang, J. Wang, G. Chen, *J. Colloid Interface Sci.* **2024**, *654*, 731-739.

[26] C. Lei, Z. Chen, T. Jiang, S. Wang, W. Du, S. Cha, Y. Hao, R. Wang, X. Cao, M. Gong, *Angew. Chem. Int. Ed.* **2024**, *63*, e202319642.

[27] P. Zhou, X. Liu, Z. Chen, C. Tang, X. Zhao, J. Zheng, R. Ge, H. Duan, *Adv. Funct. Mater.* **2025**, 2502081.

[28] R. Latsuzbaia, R. Bisselink, A. Anastasopol, H. Van der Meer, R. Van Heck, M. S. Yagüe, M. Zijlstra, M. Roelands, M. Crockatt, E. Goetheer, *J. Appl. Electrochem.* **2018**, *48*, 611-626.

[29] Y.-L. Fan, H.-F. Zhao, J.-X. Wang, W.-H. Li, F. Wei, M. Liu, Y.-H. Yu, F. Yu, W.-T. Lu, G. Zhang, *ACS Sustainable Chem. Eng.* **2024**, *12*, 3256-3264.

[30] S. Klinyod, N. Yodsin, M. T. Nguyen, Z. Pasom, S. Assavapanumat, M. Ketkaew, P. Kidkhunthod, T. Yonezawa, S. Namuangruk, C. Wattanakit, *Small* **2024**, *20*, 2400779.

[31] F. B. Liu, N. Lin, L. C. Cong, X. X. Li, F. Y. Han, D. Y. Xin, H. B. Lin, *ChemCatChem* **2023**, *15*, e202300765.

[32] Y. Zhou, Y. Shen, H. Li, *Appl. Catal. B Environ. Energy* **2022**, *317*, 121776.

[33] Y. Ramli, V. Chaerusani, Z. Yang, Z. Feng, S. Karnjanakom, Q. Zhao, S. Li, Y. Li, A. Abudula, G. Guan, *J. Environ. Chem. Eng.* **2024**, *12*, 113666.

[34] J. Wang, W. Zhao, H. Yu, W. Wang, Y. Xu, L.-L. Shen, G.-R. Zhang, D. Mei, *Appl. Catal. B Environ. Energy* **2024**, *353*, 124086.

[35] G. Zhao, G. Hai, P. Zhou, Z. Liu, Y. Zhang, B. Peng, W. Xia, X. Huang, G. Wang, *Adv. Funct. Mater.* **2023**, *33*, 2213170.

[36] Y. Wang, H. He, H. Lv, F. Jia, B. Liu, *Nat. Commun.* **2024**, *15*, 6761.

[37] P. Zhou, X. Lv, S. Tao, J. Wu, H. Wang, X. Wei, T. Wang, B. Zhou, Y. Lu, T. Frauenheim, X. Fu, S. Wang, Y. Zou, *Adv. Mater.* **2022**, *34*, e2204089.

[38] D. Chen, Y. Ding, X. Cao, L. Wang, H. Lee, G. Lin, W. Li, G. Ding, L. Sun, *Angew. Chem. Int. Ed.* **2023**, *62*, e202309478.
